# The plant microbiome: From ecological foundations to precision microbial engineering for sustainable agriculture

**DOI:** 10.1002/imt2.70152

**Published:** 2026-08-02

**Authors:** Mi Wei, Tengxiang Lian, Liying Chen, Lanxiang Wang, Junjie Ye, Xiaofang Yao, Shilong Duan, Ziyu Lu, Jinji Tu, Hongfu Li, Xin‐Yue Xu, Jie Zhou, Junliang He, Feiying Zhu, Paola Bonfante, Lam‐Son Phan Tran, Zhiqiang Pang, Xin Zhou, Zhihui Xu, Lin Zhang, Mengcen Wang, Xiaolin Wang, Chang‐Fu Tian, Yong‐Xin Liu, Kai Sun, Ertao Wang, Xianan Xie

**Affiliations:** ^1^ Guangdong Basic Research Center of Excellence for Precise Breeding of Future Crops, College of Forestry and Landscape Architecture South China Agricultural University Guangzhou China; ^2^ School of Agriculture and Biotechnology Sun Yat‐sen University Shenzhen China; ^3^ Institute of Plant Protection, Beijing Key Laboratory of Environment Friendly Management on Fruit Diseases and Pests in North China, Key Laboratory of Environment Friendly Management On Fruit and Vegetable Pests in North China (Co‐Construction By Ministry and Province) Beijing Academy of Agriculture and Forestry Sciences Beijing China; ^4^ State Key Laboratory for Development and Utilization of Forest Food Resources, State Key Laboratory of Tree Genetics and Breeding, The Southern Modern Forestry Collaborative Innovation Center College of Life Sciences, Nanjing Forestry University Nanjing China; ^5^ New Cornerstone Science Laboratory, Laboratory of Plant Carbon Capture, CAS Center for Excellence in Molecular Plant Sciences, Institute of Plant Physiology and Ecology Chinese Academy of Sciences Shanghai China; ^6^ State Key Laboratory of Tropical Crop Breeding, Genome Analysis Laboratory of the Ministry of Agriculture and Rural Affairs, Agricultural Genomics Institute at Shenzhen Chinese Academy of Agricultural Sciences Shenzhen China; ^7^ State Key Laboratory of Nutrient Use and Management; College of Resources and Environmental Sciences; Key Laboratory of Plant‐Soil Interactions, Ministry of Education China Agricultural University Beijing China; ^8^ Earth and Life Institute Université catholique de Louvain, Applied Microbiology, Mycology Louvain‐la‐Neuve Belgium; ^9^ College of Agriculture and Biotechnology Zhejiang University Hangzhou China; ^10^ Jiangsu Key Laboratory for Pathogens and Ecosystems, Jiangsu Engineering and Technology Research Center for Industrialization of Microbial Resources College of Life Sciences, Nanjing Normal University Nanjing China; ^11^ Yuelushan Laboratory Hunan Academy of Agricultural Sciences Changsha China; ^12^ Department of Life Sciences and Systems Biology University of Turin Turin Italy; ^13^ Institute of Genomics for Crop Abiotic Stress Tolerance, Department of Plant and Soil Science Texas Tech University Lubbock Texas USA; ^14^ CAS Key Laboratory of Tropical Plant Resources and Sustainable Use, Xishuangbanna Tropical Botanical Garden Chinese Academy of Sciences Mengla China; ^15^ Plant Genetics, TUM School of Life Sciences Technical University of Munich Freising Germany; ^16^ State Key Laboratory of Microbial Diversity and Innovative Utilization, Institute of Microbiology Chinese Academy of Sciences Beijing China; ^17^ Jiangsu Provincial Key lab for Organic‐based Fertilizer Creation and Soil Health Manipulation, Jiangsu Provincial Key Laboratory of Coastal Saline Soil Resources Utilization and Ecological Conservation, Educational Ministry Engineering Center of Resource‐Saving Fertilizers Nanjing Agricultural University Nanjing China; ^18^ State Key Laboratory of Plant Environmental Resilience, MOA Key Laboratory of Soil Microbiology, Rhizobium Research Center, College of Biological Sciences China Agricultural University Beijing China

**Keywords:** microbiome assembly, mycorrhizal symbiosis, pathobiome, plant microbiome, phyllosphere, rhizosphere, synthetic community

## Abstract

Plants are best understood as evolutionary holobionts, in which the host and its associated microbiomes operate as an integrated unit to influence growth, health, and stress resilience. This comprehensive review synthesizes the most current knowledge of plant‐associated microbiomes across key ecological compartments, including the rhizosphere, endosphere, phyllosphere, and seeds, highlighting their assembly drivers, functional mechanisms, and translational potential. We dissect the molecular foundations of rhizobial and arbuscular mycorrhizal (AM) symbioses, the plant‐AM fungus‐bacterium continuum, alongside emerging concepts including the aerial root mucilagesphere, phyllosphere homeostasis, and the pathobiome. We further explore host genetic, metabolic, and environmental determinants of microbiome assembly, and present cutting‐edge methodologies ranging from quantitative profiling to artificial intelligence‐driven synthetic community design. Finally, we outline a strategic blueprint for harnessing standardized synthetic microbiomes and precision microbiome engineering to advance sustainable agriculture. This integrative framework bridges fundamental ecology with practical applications, delineating a path toward climate‐resilient crop production.

## Highlights

- The plant microbiome is shaped by host genetic loci termed “*M* genes” (microbiome genes), which direct the synthesis of specialized metabolites to selectively recruit beneficial microbes and maintain microbiome homeostasis.
- The plant establishes a tripartite continuum with arbuscular mycorrhizal fungi and bacteria, engages in cross‐kingdom RNA interference, and employs effector‐driven communication during mycorrhizal and rhizobial symbioses.
- The plant microbiome extends to the aerial root mucilagephere as a unique microbial habitat and reframes disease through the pathobiome lens, emphasizing cooperative virulence networks.
- The plant microbiome can be dissected using cutting‐edge methodologies, including quantitative microbiome profiling, precision agricultural microbiome engineering, and artificial intelligence (AI)‐enabled synthetic community design.
- The plant microbiome offers translational potential through standardized synthetic microbiome products, synbiotic strategies, and microbiome‐informed breeding to overcome field deployment bottlenecks and advance sustainable agriculture.

## Graphical abstract

From ecological principles to precision engineering of plant microbiomes for sustainable agriculture. The plant holobiont integrates diverse microbial niches (including rhizosphere, endosphere, phyllosphere, seed, and aerial‐root mucilagesphere), host genetics (namely *M* genes), and host metabolites. Furthermore, molecular dialogues, including rhizobial and mycorrhizal symbioses, cross‐kingdom RNA interference, and effector‐mediated communication, drive beneficial associations, while the pathobiome concept reframes disease as a community‐level process. Importantly, cutting‐edge methodologies (e.g., quantitative microbiome profiling and artificial intelligence‐driven synthetic microbial community design) enable predictive manipulation. Finally, the translational pathway encompasses standardized synthetic microbiome products, synbiotic strategies, and microbiome‐guided breeding, aiming to enhance stress resilience, reduce fertilizer and pesticide inputs, and stabilize field performance. Taken together, this framework bridges fundamental ecological principles with practical applications for climate‐resilient crop production.

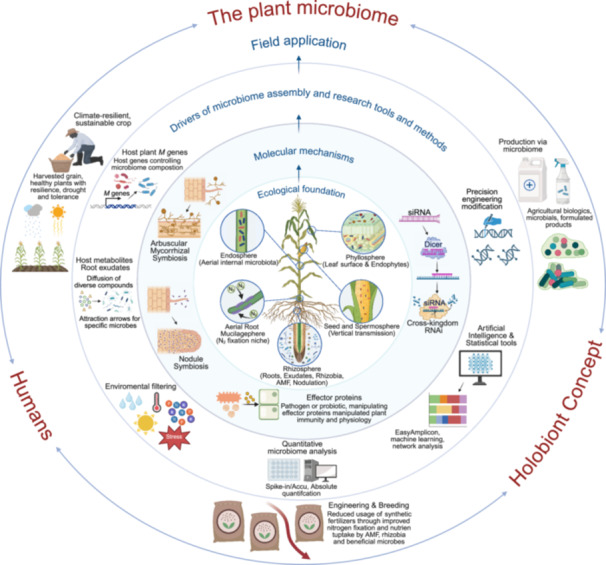

## INTRODUCTION

**FIGURE 1:**
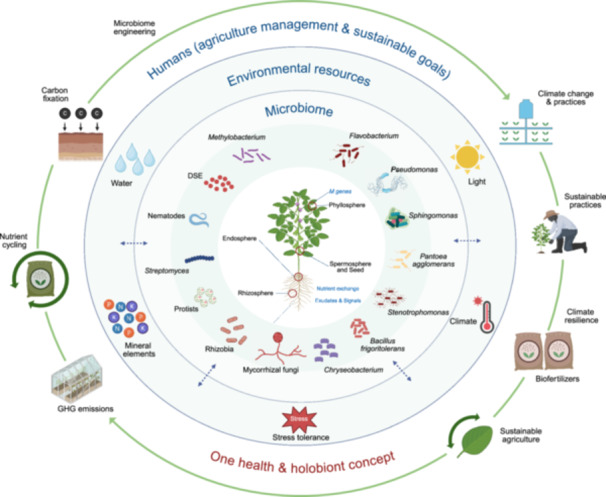
The plant holobiont: A multiple life community integrating plants, microbiomes, environmental resources, and human governance. The plant holobiont is depicted as a nested system where the host plant (center) intimately associates with diverse microbial communities across different niches, including rhizosphere, endosphere, phyllosphere, and seed. These microbes engage in bidirectional chemical interactions and nutrient exchanges, shaped by host genetic loci (“*M* genes”). Environmental resources (e.g., soil, water, light, nutrients, and climate) provide inputs and constraints, while microbial activities feed back to ecosystem processes (e.g., carbon cycling, nitrogen cycling, and stress alleviation). Human interventions (e.g., microbiome engineering and sustainable agricultural practices) aim to harness these interactions for climate resilience and food security. This framework emphasizes the co‐evolutionary and interdependent relationships that define the plant holobiont as a unified evolutionary and functional unit. *M* genes, microbiome genes; DSE, dark septate endophytes; GHG, greenhouse gases.

Plants are not autonomous entities but complex holobionts (Figure 1), thriving in intimate association with a vast and diverse consortium of microorganisms, collectively known as the plant microbiome [1, 2]. This microbial consortium, including bacteria, fungi, archaea, protists, and viruses, colonizes every accessible plant tissue, from the below‐ground rhizosphere to the above‐ground phyllosphere, forming a “second genome” that significantly extends the host plant's metabolic and functional capabilities [1, 3, 4]. Over the past two decades, advances in high‐throughput sequencing, metagenomics, and other multi‐omics approaches have revolutionized our understanding of plant‐associated microbiomes, revealing their profound influence on plant development, nutrient acquisition, and resilience to diverse biotic and abiotic stresses [2, 5, 6, 7]. Therefore, recognizing that plants and their microbiome as a single evolutionary unit, the holobiont (Figure 1), has fundamentally reshaped our understanding of plant biology, ecology, and evolution [1, 8].

The co‐evolutionary history between plants and their microbial partners dates back more than 400 million years, to the very origin of land colonization [9]. This ancient association gave rise to a sophisticated chemical dialogue that orchestrates microbiome assembly and function [10]. Plants actively shape their microbiota through root and foliar exudates, including sugars, amino acids, organic acids, and other specialized metabolites, that selectively recruit beneficial microbes while repelling pathogens [10, 11, 12, 13, 14]. The plant microbiome is also a highly structured and dynamic ecosystem shaped by the interplay of host genetics, microbial interactions, and environmental factors [15, 16, 17, 18]. Key host genetic loci, known as “*M* genes” (microbiome genes) (Figure 1), govern the assembly of specific microbial taxa under stressed conditions, directly connecting plant genotype to microbiome composition and function [19, 20, 21]. For example, *OsC4H2* and *OsPAL06* in rice (*Oryza sativa* L.) regulate the synthesis of ferulic acid that selectively enriches beneficial Burkholderiales in the phyllosphere, thereby suppressing pathogens and preventing dysbiosis [22]. This host‐mediated selection underscores a fundamental principle of plant‐microbiome ecology: plants and their microbiomes have co‐evolved to maximize fitness across diverse environments [23, 24].

Recent decades have witnessed an explosion in plant microbiome research [5, 25, 26]. Early studies, spanning approximately from 2010 to 2018, were largely dedicated to providing a taxonomic catalog of the microbial communities inhabiting distinct plant niches, such as the rhizosphere, phyllosphere, and endosphere [16, 27, 28]. Subsequently, researches effort shifted towards elucidating the assembly mechanisms of these microbial communities and the host genetic principles governing them [2, 29]. A general observation from these studies is that some microbial components, such as nitrogen‐fixing rhizobia and mycorrhizal fungi, maintain stable and specific colonization patterns within or on plant roots. In contrast, many other microorganisms do not exhibit such defined patterns; they may either reside on the surface of plant organs (epiphytes) or colonize internal tissues (endophytes) in a more variable manner [30, 31]. These studies have unveiled the immense taxonomic and functional diversity of plant microbiota and have illuminated their critical roles in key ecosystem processes, from global carbon and nitrogen cycling to the suppression of plant diseases, as well as enhancing plant nutrient acquisition, growth, health, and yield [31, 32, 33, 34]. While several highly cited reviews published before 2020 have comprehensively summarized the taxonomic composition and environmental drivers of plant microbiomes [2, 5, 16], many of these earlier works primarily focused on descriptive community profiling and lacked in‐depth mechanistic dissection of host‐microbiome interactions at the molecular level. Moreover, the translational potential of emerging tools such as synthetic communities, machine learning, and precision genome editing has largely been unexplored in those summaries. Over the past 5 years, the field has witnessed transformative advances, including the discovery of host “*M* genes” that actively shape the microbiome [19, 21], the application of artificial intelligence for predictive community design [35, 36], and the conceptual shift from a single‐pathogen paradigm to the community‐level pathobiome framework [37]. These breakthroughs have fundamentally shifted plant microbiome research from a descriptive cataloging exercise toward a predictive and manipulative science.

Despite these significant advances, translating fundamental microbiome knowledge into sustainable agricultural and forestry practices remains a major challenge [38, 39]. A key bottleneck is the inconsistent performance of microbial inoculants under field conditions, often resulting from poor colonization, competition with native microbiota, and prevailing environmental factors [40, 41]. Furthermore, a major knowledge gap is our limited understanding of the intricate microbiome interactions and the plant genetic mechanisms that govern host‐driven microbial recruitment [5, 42]. This challenge is compounded by the unculturability of the vast majority of microorganisms and a predominant focus on high‐level taxonomy rather than strain‐level resolution, despite evidence from microbiome research that functional relevance is strain‐dependent [43], thereby hindering the link between microbial activities and actual plant outcomes. Additionally, the lack of standardized and compatible data frameworks limits the integration of microbiome information into precision agriculture and predictive models [5]. Therefore, a comprehensive understanding that integrates the ecology, assembly, mechanism, and functions of microbiomes across plant niches is essential to harness the plant microbiome for sustainable agriculture.

**FIGURE 2:**
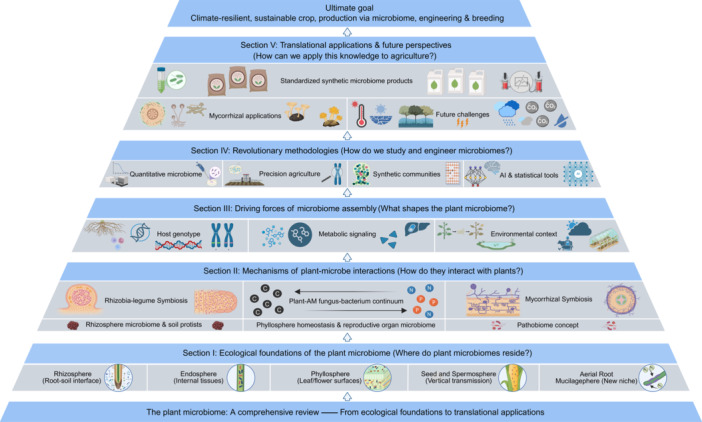
A diagram of the conceptual framework to illustrate the contents of this review. The framework of this comprehensive review is organized into five hierarchical sections that collectively build from foundational knowledge toward translational goals. Section I maps the ecological habitats where plant microbiomes reside, which include the rhizosphere, endosphere, phyllosphere, seed, and aerial root mucilagesphere, defining the spatial boundaries of plant microbiomes. Section II examines the molecular mechanisms underlying core plant–microbe interactions, spanning symbiotic signaling, nutrient exchange, and pathobiome dynamics. Section III identifies the principal forces, including host genotype, metabolic signaling, and environmental context, that drive the composition and dynamics of plant microbiomes. Section IV presents the revolutionary methodologies that are transforming microbiome research, spanning quantitative microbiome profiling with absolute quantification, precision agriculture through cultivar‐specific approaches, synthetic communities targeting keystone taxa, and artificial intelligence (AI) and statistical tools. Section V translates these insights into applications and future perspectives, highlighting standardized synthetic microbiome products, mycorrhizal applications, and associated challenges including asymbiotic AMF cultivation, regulatory frameworks, mycorrhiza‐friendly practices, strain compatibility, tripartite consortia, and field performance stability. Collectively, this framework converges on the ultimate goal of achieving climate‐resilient and sustainable crop production through microbiome engineering and breeding. Accu, absolute quantification; AI, artificial intelligence; AMF, arbuscular mycorrhizal fungus; AM, arbuscular mycorrhiza; BXs, benzoxazinoids; DBTL, design‐build‐test‐learn cycle; ECM, ectomycorrhiza; ERM, ericoid mycorrhiza; *M* genes, microbiome genes; mGWAS, microbiome genome‐wide association study; MWAS, metabolome‐wide association study; ORM, orchid mycorrhiza; Spike in, Spike‐in method; SynCom, synthetic microbial community.

In this review, we present the most current knowledge to provide a comprehensive framework for understanding the plant microbiome (Figure 2). We begin by detailing the ecological foundations of key plant compartments, highlighting their unique community structures and functions. We then explore the major drivers of microbial community assembly, including host genetics and environmental factors. Subsequently, we discuss cutting‐edge methodologies, from quantitative microbiome profiling to artificial intelligence (AI)‐driven synthetic community design, that are revolutionizing the field. Finally, we highlight the translational applications of plant microbiome research and outline a roadmap for harnessing microbial functions to promote sustainable agriculture under climate change.

## ECOLOGICAL FOUNDATIONS OF THE PLANT MICROBIOME

**FIGURE 3:**
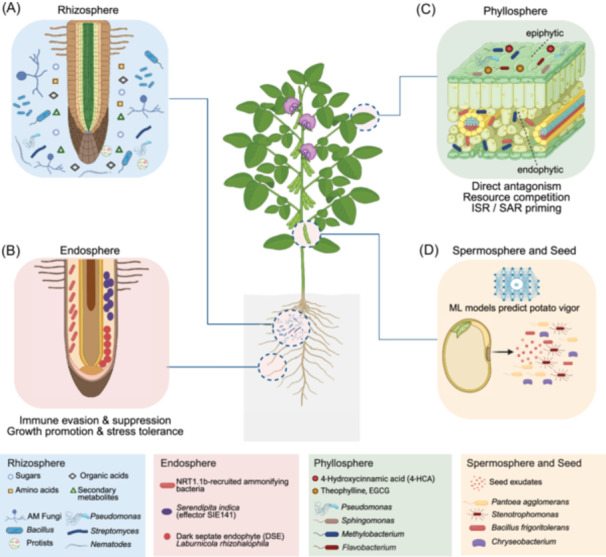
Ecological foundations of the plant microbiome: Compartment‐specific community structures and functions. The plant microbiome is ecologically structured into distinct compartments: rhizosphere, endosphere, phyllosphere, and spermosphere/seed. (A) Rhizosphere: Root exudates fuel a microbial hotspot enriched with *Pseudomonas*, *Bacillus*, *Streptomyces*, AM fungi, and soil fauna (e.g., protists and nematodes). (B) Endosphere: Internal plant tissues harbor specialized endophytes, including *Serendipita indica* and dark septate endophytes (e.g., *Laburnicola rhizohalophila*). (C) Phyllosphere: Aerial surfaces host functionally significant bacteria (e.g., *Pseudomonas*, *Sphingomonas*, and *Flavobacterium*) and fungi, shaped by host metabolites (e.g., 4‐hydroxycinnamic acid and theophylline) and environmental extremes. Epiphytic communities are more dynamic than endophytic ones, contributing to pathogen suppression and systemic resistance. (D) Spermosphere/Seed: Seed exudates recruit beneficial microbes (e.g., *Pantoea* and *Bacillus*) that enhance germination, suppress pathogens, and vertically transmit beneficial traits across generations. Therefore, these compartment‐specific ecological foundations provide a roadmap for targeted microbiome manipulation toward sustainable agriculture. EGCG, epigallocatechin gallate; ISR, induced systemic resistance; ML, machine learning; NRT1.1b, nitrate transporter 1.1b; SAR, systemic acquired resistance; SIE141, *Serendipita indica* effector 141; EGCG, epigallocatechin gallate.

The plant microbiome is ecologically structured into distinct compartments, such as the rhizosphere, endosphere, phyllosphere, and seed, each offering a unique habitat defined by specific physicochemical conditions, nutrient profiles, and host‐driven selective pressures (Figure 3A–D). These specific compartments act as ecological filters that govern microbial recruitment and shape the composition, diversity, and functional potential of resident microbial communities. Elucidating these ecological foundations is essential for understanding microbiome assembly and for enabling targeted manipulation for sustainable agriculture.

### Rhizosphere

The rhizosphere, the narrow zone of soil influenced by plant roots (Figure 3A), is considered a hot spot of microbial diversity and activity [44, 45]. This region is characterized by a high flux of root exudates, a complex mixture of sugars, amino acids, organic acids, secondary metabolites, and volatile organic compounds, which serve as a primary carbon source for microbial growth and act as key signals for microbial recruitment [11, 46, 47]. Plant genotype and developmental stage shape a distinct rhizosphere community enriched in growth‐promoting and nutrient‐solubilizing genera, for example, *Pseudomonas*, *Bacillus*, *Rhizobium*, *Streptomyces*, *Massilia*, and *Flavobacterium*, compared to the bulk soil [48, 49, 50, 51]. A stable, host‐heritable core microbiome, including *Pseudomonas*, *Bacillus*, and *Streptomyces*, consistently inhabits the rhizosphere of many plant species and is critical to plant health [52, 53]. Furthermore, the rhizosphere is a nexus for complex interactions, including those with arbuscular mycorrhizal (AM) fungi, which form symbioses with 72% of land plants to enhance nutrient (particularly phosphorus and nitrogen) and water uptake [54, 55]. The hyphae of AM fungi extend the rhizosphere into a “hyphosphere,” forming a physical and functional continuum that harbors a distinct bacterial community and facilitates bidirectional nutrient exchange, thereby linking plants, AM fungi, and bacteria in a tripartite interaction—a concept defined as the plant‐AM fungus‐bacterium continuum [56, 57, 58, 59]. Beyond bacteria and fungi, the rhizosphere is also a habitat for soil fauna such as protists and nematodes, as well as viruses, all of which can influence plant health by modulating microbial communities through grazing, lysis, and gene transfer, thereby shaping their composition and function [60, 61, 62, 63].

In addition to the rhizosphere, microorganisms can colonize the rhizoplane (the root surface), which represents a distinct microbial habitat compared to the surrounding rhizosphere soil, functioning as a transitional stage for microorganisms moving from the rhizosphere into the root endosphere [1, 64]. Microbial cells (e.g., rhizobia and ectomycorrhizal fungi) are often irregularly distributed, with root tips, elongation zones, and lateral root cracks serving as preferential colonization sites [65, 66]. This compartment is of particular importance because the rhizoplane constitutes the portal of entry for microorganisms into the root endosphere [1].

### Endosphere

Endophytic microbes reside within plant tissues, colonizing the intercellular spaces and even the xylem (Figure 3B), creating a more stable and protected environment than the surface [31]. The endosphere represents a bottleneck that selects for specific microbial taxa that have evolved mechanisms to evade or suppress plant immune responses [67, 68]. Fungi and bacteria are the primary colonizers in plant endosphere. Among them, the core bacterial endophytes belong to the phyla Proteobacteria, Actinobacteria, and Firmicutes, and are known to produce bioactive compounds and exhibit growth‐promoting properties, thereby promoting plant growth and enhancing plant stress tolerance [69, 70]. For example, the rice nitrate transporter NRT1.1B functions as a master regulator of root endophytic microbiome by recruiting a community enriched in ammonifying bacteria (bacteria that convert organic nitrogen to ammonia), thereby improving nitrogen use efficiency of rice [71]. Additionally, diverse fungal endophytes extensively establish interactions with their plant hosts and provide benefits to them. For example, the root endophyte *Colletotrichum tofieldiae*, can confer phosphate status‐dependent fitness benefits to its host [72]. Beyond *C. tofieldiae*, *Serendipita indica* (formerly *Piriformospora indica*) has emerged as a prominent model endophyte, distinguished by its broad host range and dual functions in growth promotion and stress tolerance across diverse crops [73]. For example, *S. indica* secretes effector proteins such as SIE141 that translocate to the plant nucleus to modulate host immunity and salt stress tolerance [74]. Furthermore, dark septate endophytes (DSEs), a group of melanized fungi, frequently colonize plant roots in stress‐prone environments [75, 76]. Recent studies have revealed that DSE strains such as *Laburnicola rhizohalophila* possess a rare hybrid heterozygosity that confers superior adaptability to saline‐alkali stress and promotes host growth [77]. Collectively, these findings highlight the functional diversity of fungal endophytes and their substantial potential for sustainable agriculture [78].

### Phyllosphere

The phyllosphere, comprising the aerial surfaces of stems, leaves, flowers, and fruits (Figure 3C), represents the largest biological interface on earth [79, 80]. This habitat is exposed to harsh environmental conditions, including fluctuating temperature, humidity, and ultraviolet radiation, which exert strong selective pressure on microbial colonizers [81, 82]. The phyllosphere hosts less diverse microbial communities than the rhizosphere, dominated by a few key bacterial phyla such as Proteobacteria (particularly *Pseudomonas*, *Sphingomonas*, and *Methylobacterium*), Actinobacteria, and Firmicutes, as well as fungal Ascomycota [83, 84]. Notably, epiphytic communities residing on leaf surfaces exhibit greater richness, diversity, and greater temporal instability across plant developmental stages compared to endophytic communities residing inside leaf tissues, with divergence between these two niches emerging primarily at the flowering stage [85]. Host genetics and leaf chemistry play a crucial role in shaping these microbial communities [19, 86]; for example, the phenylpropanoid pathway in rice produces 4‐hydroxycinnamic acid, a metabolite that selectively enriches beneficial *Pseudomonas* while inhibiting pathogens to maintain microbiome homeostasis [19, 22]. Emerging evidence from tea plants reveals that leaf secondary metabolites such as theophylline and epigallocatechin gallate temporally orchestrate the assembly of phyllosphere microbiota by recruiting *Flavobacterium*, *Myriangium*, *Parabacteroides*, and *Mortierella* to suppress fungal pathogens, highlighting the functional relevance of metabolite‐driven microbial assembly [87].

The phyllosphere microbiome defends against foliar pathogens through direct antagonism, resource competition, and induced systemic resistance [88, 89, 90]. Beyond direct antagonism, specific phyllosphere bacteria such as *Flavobacterium* sp. are recruited to *Arabidopsis* leaves via a salicylic acid (SA)‐dependent pathway, where they activate systemic acquired resistance and thereby connect local microbial colonization with whole‐plant immune priming [91]. Similarly, the floral and fruit microbiomes are critical for plant reproduction and seed health, with specific microbes vertically transmitted to the next generation [92, 93]. Collectively, the phyllosphere microbiome functions as a dynamic and environmentally responsive interface that integrates host genetic control, metabolite signaling, and microbial functional traits to sustain plant health and productivity.

### Seed

The seed, serving as a carrier of the initial plant‐associated microbiomes (Figure 3D), harbors a diverse range of microbial communities, comprising both internal endophytes and surface‐dwelling epiphytes [94, 95]. Whereas the spermosphere, defined as the narrow zone of soil surrounding a seed, serves as a dynamic interface for early seed‐microbe interactions [94]. Moreover, the seed microorganisms constitute important sources for the future plant microbiome, including rhizosphere, endosphere, and phyllosphere microbiome [94, 96]. A recent study has demonstrated that seed endophytes are vertically transmitted across three wheat generations, as evidenced by the recovery of GUS‑labelled *Pantoea agglomerans* from seeds of each generation [97]. In rice, seed bacteria can be acquired from the external environment during panicle heading and flowering, colonizing the interspace between caryopsis and glumes rather than the caryopsis itself [98]. A large‐scale analysis of tomato genotypes identified host genotype as the primary determinant of seed microbiome structure [99]. In another example, genetically distinct fonio millet populations share diverse seed endophytic microbiomes, including core *Pseudomonas* and *Bacillus* taxa that are vertically transmitted from parent to progeny [100]. Moreover, seed microbiota can exhibit heterosis, with hybrid seeds containing more diverse and abundant microbial communities than their parents [99, 101]. Seed microbial communities are also altered by domestication and breeding, as evidenced by the higher bacterial diversity in low‐domestication *Cannabis* genotypes than modern inbred lines [102].

Generally, the core seed microbiome can influence seed dormancy and germination, seedling vigor, and early disease resistance [100, 103, 104], by means of mechanisms including phytohormone crosstalk [105]. Upon seed germination, specific microbes undergo stress‐responsive “resuscitation” from the seed symbiotic toolbox, while specialists target specific stresses and recruit adapted partners to enhance seedling fitness [106]. Moreover, the seed microbiome can predict agronomic traits; machine learning models based on seed tuber microbiome accurately forecast next‐season potato vigor, with *Streptomyces*, *Acinetobacter*, and *Cellvibrio* amplicon sequence variants as key indicators [107]. Together, these findings highlight the potential of harnessing seed‐associated microbiomes for sustainable crop production through microbiome‐informed breeding and precision agriculture [96].

## GLOBAL ATLAS OF PLANT MULTI‐HABITAT MICROBIOME: MECHANISMS OF PLANT–MICROBE INTERACTIONS

### Rhizosphere microbiome

Conventional rhizosphere microbiome research has predominantly focused on rhizoplane‐colonizing microbial communities and their growth‐promoting effects. However, with the rapid development of metagenomics, spatial metabolomics, and synthetic microbial communities (SynComs) approaches, the field is undergoing a conceptual shift from a simple root–soil binary interface model toward a multidimensional network of interactions [108]. Contemporary rhizosphere biology has transcended classical boundaries to encompass the endorhiza (root endosphere), mycorrhizosphere, hyphosphere, and such as those associated with aerial roots, with increasing emphasis on soil fauna‐mediated multitrophic regulations and cross‐kingdom interaction mechanisms [109]. In particular, advances in mycorrhizal symbiosis research have illuminated the co‐evolutionary relationships within a biological continuum formed by plant root, AM fungi, and their associated bacteria, elucidating their coordinated roles in nutrient mobilization, immune modulation, and systemic induced resistance [59, 110]. This section integrates current theoretical advances of representative rhizosphere microorganisms, and constructs a systematic framework spanning molecular mechanisms to ecosystem functions, with the aim of providing a scientific foundation for the precision manipulation of rhizosphere communities to enhance crop nutrient‐use efficiency, stress resilience, and ecological adaptability.

### Rhizobia: The facultative endosymbionts of legumes

#### Overview

**FIGURE 4:**
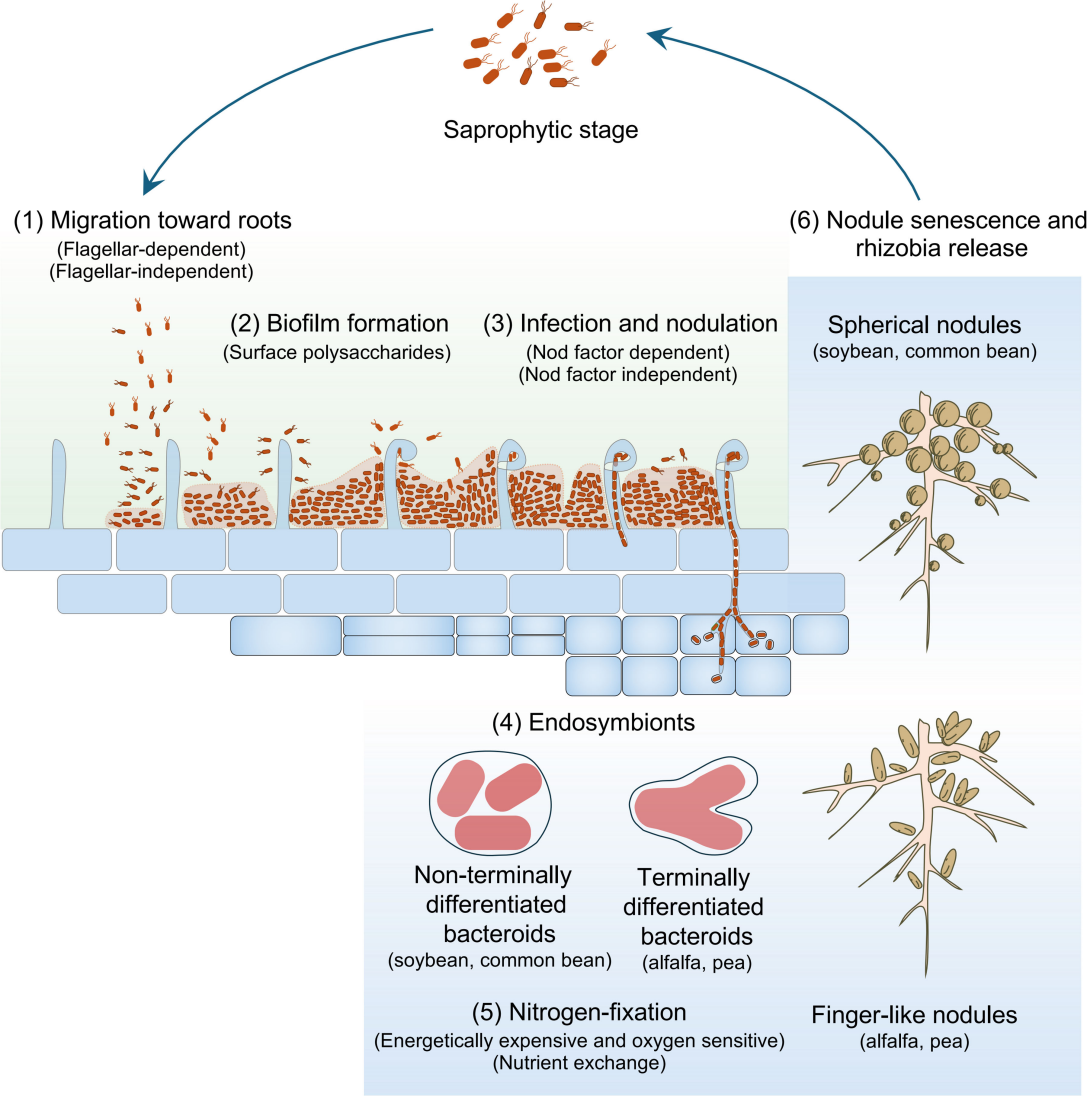
Rhizobia: The facultative endosymbionts of legumes. In the presence of compatible legume hosts, the life cycle of rhizobia can be briefly divided into the following successive stages: (1) Saprotrophic free‐living rhizobia migrate toward roots via flagellar‐dependent and/or flagellar independent motility; (2) Rhizobia switch from a motile to a sessile stage on the rhizoplane by forming biofilms with surface polysaccharides as the major matrix; (3) Rhizobia induce nodule organogenesis and infect nodule cells intracellularly via either Nod factor‐dependent or ‐independent mechanisms; (4) Under the control of the host legume species (as indicated in brackets), rhizobia undergo either non‐terminal or terminal differentiation into nitrogen‐fixing bacteroids; (5) Nitrogen fixation is energetically costly and oxygen‐sensitive, requiring carbon and other nutrients from host cells; (6) Upon senescence of both spherical and finger‐like nodules on different legume species, rhizobia are released into the soil and resume a saprotrophic lifestyle.

Rhizobia are a polyphyletic group of soil bacteria that form mutually beneficial symbiotic relationships with leguminous plants, triggering nodule formation on roots or stems and converting atmospheric nitrogen into ammonia for host utilization. This symbiosis is the most efficient biological nitrogen fixation system in natural ecosystems, contributing roughly 65% of agricultural biological nitrogen input and supporting sustainable crop production [111]. Unlike obligate symbionts, rhizobia lead a facultative lifestyle: they survive saprotrophically in bulk soil and switch to a symbiotic lifestyle under compatible host signals. The symbiosis between rhizobia and legumes is a sophisticated multi‐step interaction (Figure 4), spanning rhizosphere chemotaxis, root surface colonization, infection thread formation, nodule organogenesis, and intracellular nitrogen fixation [112]. This process relies on precise molecular communication between bacteria and host plants, as well as coordinated expression of hundreds of functional gene clusters [112, 113]. Different rhizobial strains exhibit huge variations in symbiotic efficiency even when associating with the same host plant, which is shaped by strain diversity, environmental adaptability, and coordinated functions of multiple gene modules during the transition from saprotrophs to endosymbionts of legumes. This review focuses on the fundamental biological processes of rhizobial symbiosis, dissecting the genetic basis and regulatory mechanisms, to clarify the molecular logic of rhizobial adaptation and symbiotic efficiency. We further discuss the coupling of horizontal gene transfer, genome innovation, and regulatory rewiring that underlies successful symbiosis, providing a theoretical basis for developing high‐efficiency rhizobial inoculants via synthetic biology.

#### Diversity of rhizobia

Rhizobia refer to a collection of Gram‐negative bacteria belonging to α‐ and β‐proteobacteria, with over 200 validly published species across 20 genera [114]. These genera belong to Burkholderiaceae (*Paraburkholderia*, *Cupriavidus*, *Trinickia*), Methylobacteriaceae (*Methylobacterium*, *Microvirga*), Hyphomicrobiaceae (*Devosia*), Xanthobacteraceae (*Azorhizobium*), Brucellaceae (*Ochrobactrum*), Bradyrhizobiaceae (*Bradyrhizobium*), Phyllobacteriaceae (*Mesorhizobium*, *Allmesorhizobium*, *Neomesorhizobium*, *Aminobacter*, *Phyllobacterium*), and Rhizobiaceae (*Rhizobium*, *Sinorhizobium*, *Allorhizobium*, *Pararhizobium*, *Neorhizobium*, *Shinella*). Rhizobia are a polyphyletic group and symbiotic traits emerged via repeated horizontal gene transfer (HGT) events across soil bacteria [115]. Beyond phylogenetic divergence, rhizobial diversity is reflected in symbiotic types, defined by the concept of ‘symbiovar’ [116]. Symbiovars are classified by host specificity and variations in key symbiosis genes, such as nodulation genes (*nod*), regardless of genomic background [116]. Rhizobia associated with the same legume host can have *nod* genes of different phylogenies. For example, *nod* genes from *Bradyrhizobium* and *Sinorhizobium* strains nodulating soybeans exhibit genus‐dependent phylogenies [117], though Nod factors of similar features are produced by these soybean rhizobia [118], implying convergent evolution.

Molecular phylogenetic analyses consistently demonstrate that the evolutionary history of key symbiosis genes often differs markedly from that of core chromosomal genes [119, 120]. Early studies regarded HGT of key symbiotic genes as the sole driver of rhizobial symbiotic ability, but accumulating evidence proves that acquiring *nod* and nitrogen fixation (*nif*) genes alone is insufficient for effective symbiosis [121]. The recipient strain's genomic background, accessory gene functions, and dynamic regulatory networks are equally critical [117, 118, 122, 123]. For example, symbiotic performance on soybeans varies drastically among closely related *Sinorhizobium fredii* strains producing conserved structures of Nod factors [118]. Some rhizobia exhibit narrow host ranges, only infecting one or a few legume species, while broad‐host‐range strains (e.g., *S. fredii* NGR234) can colonize hundreds of legume hosts [124]. In contrast to canonical nod‐dependent symbiosis, a small number of photosynthetic and non‐photosynthetic *Bradyrhizobium* strains lack canonical *nod* genes and establish symbiosis in a Nod factor‐independent manner [125, 126]. Some Nod factor‐independent symbiotic processes require a functional Type III Secretion System (T3SS) and its effector proteins [126, 127].

#### Distribution and abundance of Rhizobia

The distribution and population size of rhizobia are not random, as revealed in global investigation of soil microbiome [128] and biogeographical studies of rhizobial community in rhizosphere soils and nodules from soybean, faba bean, and alfalfa across different ecoregions [129, 130, 131]. As expected from the niche theory, rhizobial biogeography is jointly determined by abiotic and biotic factors. Representative in situ biogeographical studies of rhizobial species have identified correlating abiotic factors, including soil chemistry (e.g., pH, iron, nitrogen, phosphorus, sulfate, boron, potassium, magnesium, calcium, electrical conductivity, salt, and organic matters) and climate (e.g., temperature of wettest and warmest month, and precipitation of driest month) [129, 130, 131, 132]. Among these abiotic conditions and resources, soil pH is the most robust acting factor across studies. This correlation has been further tested using a common garden approach, where pH adjustment was applied to soils of contrasting pH origins (e.g., acidic, neutral, and alkaline soils) and host plants (e.g., alfalfa, faba bean, and soybean) [133]. The pH neutralization treatments increase α diversity of rhizobial community in soils and enhance nodulation. Moreover, acid‐tolerant and alkaline‐adapted species in both soils and nodules can be enriched via corresponding pH adjustment treatments, though a strong selection of microsymbionts among diverse compatible rhizobia in soils by different legume hosts can be observed [133]. It is also established that pH affects the availability of diverse nutrients; for example, soluble Fe^2+^ is rare at neutral and alkaline conditions, and the pH‐dependent rhizobial biogeography may involve the integration of multiple adaptation events. This view is supported by a very recent study on *Bradyrhizobium* and *Sinorhizobium* associated with soybeans [134]. The alkaline‐adapted *Sinorhizobium* overrepresents siderophore biosynthetic functions and exhibits rapid adaptive evolution of machinery for utilizing self‐produced siderophores under iron‐limiting conditions. By contrast, the acid‐tolerant *Bradyrhizobium* rarely encodes siderophore biosynthetic genes, likely due to the abundant soluble Fe^2+^ under acidic conditions, while recruits more outer membrane receptors of different siderophores to exploit public iron‐siderophore complexes from *Sinorhizobium* and other bacteria at neutral pH conditions. The iron‐siderophore complex exploiters (e.g., *Bradyrhizobium* and the *Sinorhizobium* mutant losing siderophore biosynthetic function) show higher nodule occupancy than the siderophore producer *Sinorhizobium* under neutral pH conditions. This is in line with the biogeography of *Bradyrhizobium* and *Sinorhizobium* species associated with soybeans, which dominate in acid/neutral and alkaline soil, respectively [117, 130, 132]. The robust association of other microbes and nodulating rhizobial species across different ecoregions has not been reported, while local match has been elegantly demonstrated in the case of *Sinorhizobium* and *Bacillus* from soybeans grown in alkaline soils [135, 136]. *Bacillus cereus* specifically promotes *Sinorhizobium* growth and suppresses *Bradyrhizobium* growth; under saline‐alkali conditions, this group alleviates the stress impacts on *Sinorhizobium* nodulation and enhances its colonization inside nodules [136].

More generally speaking, α‐ and β‐proteobacteria are enriched in the core root microbiome of terrestrial plants [137, 138], suggesting that members of these lineages possess inherent traits that facilitate root colonization even in the absence of symbiosis genes. This ecological predisposition likely contributes to the repeated evolution of rhizobial symbiosis within these bacterial groups through HGT and integration of key symbiosis genes in different genomic backgrounds.

#### Rhizobial colonization

Root colonization is the prerequisite for rhizobial nodulation, consisting of the following consecutive stages: chemotaxis toward rhizosphere and host roots, adhesion to root surfaces, and biofilm formation. Chemotaxis allows rhizobia to respond to a complex cocktail of root exudates (e.g., flavonoids, amino acids, and organic acids) and move toward nutrient‐rich rhizosphere soil and rhizoplane. Flavonoids were once regarded as rhizobial chemoeffectors according to earlier studies [12], yet this notion has been questioned by recent evidence concerning the cosolvents used to solubilize flavonoids [139]. Nevertheless, there is evidence showing flavonoid‐induced expression of certain flagellar gene and surface motility in *Sinorhizobium fredii* [140]. Chemotaxis is mediated by the *che* and *mcp* gene clusters, and mutations in these loci, as well as in flagellar genes, differentially compromise rhizosphere and rhizoplane colonization efficiency under laboratory conditions in a rhizobium‐host dependent manner [141, 142, 143, 144]. Mycelial network of biotrophic fungi, for example, *Phomopsis liquidambaris* and *Rhizophagus irregularis*, can facilitate rhizobial migration toward legume roots from far distances (>10 cm), and rhizobial chemosensory system and flagellum‐related genes can be upregulated by fungi [145]. In addition to flagellar‐dependent swimming and swarming, other surface motility processes can be involved during the migration from rhizosphere to rhizoplane, for example, pilus‐dependent twitching, adhesin‐dependent gliding, and sliding driven by growing cells and other factors (e.g., surfactant). For example, exogenous or endogenous long‐chain quorum‐sensing AHLs (N‐acylated‐l‐homoserine lactones) can serve as biosurfactants to improve *Sinorhizobium fredii* migration toward soybean roots in the absence of flagellar filament [143, 146].

In contrast to the directional migration from bulk soil to rhizosphere and roots, the set of gene families determining rhizobial fitness in rhizosphere and on rhizoplane is supposed to be more flexible due to ever‐fluctuating conditions and resources in these microhabitats during the development of host plants. Available transcriptomic and Tn‐seq studies, followed by scattered reverse genetics evidence, have identified various functional genes modulating rhizobial persistence in rhizosphere and on rhizoplane in a rhizobium‐host dependent manner [141, 143, 144, 147]. These involve biosynthesis (e.g., amino acids, ribonucleotides, vitamins, and cytochrome *c*), transport (e.g., thiamine, cyclic β−1−2‐glucan, copper, phosphate, ferric iron, zinc, potassium, oligopeptide, and long‐chain N‐acylated‐l‐homoserine lactones), metabolism (e.g., gluconeogenesis, pentose phosphate pathway, and catabolism of phosphonate, erythritol, rhamnose, glycerol and arabinose), cell surface (e.g., lipoprotein, lipopolysaccharides, capsular polysaccharides, succinoglycan, galactoglucan, and mixed‐linkage (1 → 3)(1 → 4)‐β‐d‐glucan), oxidative stress response, repair of DNA and protein, and related signaling factors including the global regulator MucR/RosR and various pathway‐specific regulators, sigma factors, anti‐sigma factors, and two‐component systems (e.g., quorum sensing systems and c‐di‐GMP signaling modules) [134, 141, 143, 144, 145, 148, 149, 150]. Notably, these functional genes are identified under simplified laboratory conditions. Moreover, most identified functional genes have not been characterized in detail regarding their contribution to the fitness in these microhabitats.

Among a few well‐characterized examples, c‐di‐GMP inhibits motility and promotes the synthesis of various surface polysaccharides (e.g., succinoglycan EPS I, cryptic polysaccharide APS, and mixed‐linkage β‐glucan MLG), and drives the lifestyle switch of rhizobia from motile to sessile [149, 151, 152, 153, 154, 155]. Quorum‐sensing systems negatively regulate motility and exert either positive or negative regulation on different surface polysaccharides, leading to either enhanced or decreased biofilm formation under different conditions [156, 157, 158, 159, 160, 161, 162]. FadL‐ExoFQP is a novel module regulating the level of extracellular long‐chain AHLs, and the long‐chain AHLs over‐producing *fadL* mutant showed enhanced rhizosphere persistence, migration toward roots, rhizoplane colonization rate, and nodule occupancy on soybeans [143]. The better nodule occupancy of the *fadL* mutant is also verified in the *Sinorhizobium meliloti*‐alfalfa pair [143]. More detailed characterization and engineering of candidate gene modules is required to enhance the competitive ability of rhizobial inoculants in the presence of diverse indigenous low efficient rhizobia and other microorganisms under field conditions.

#### Rhizobial nodulation and nitrogen fixation

In most rhizobium‐legume pairs, canonical *nod* genes are the foundation of host recognition and infection initiation [113]. The core NodABC proteins synthesize the backbone of Nod factors (NFs), N‐acetylglucosamine oligosaccharide signals that trigger host nodule organogenesis. NodC controls NF backbone length, and NodA transfers fatty acid chain onto the deacetylated NF backbone generated by the deacetylase NodB [163, 164]. NodIJ mediate NF secretion, while other accessory Nod proteins modify NF structures, determining host specificity [165, 166]. *nodD* is the master regulatory gene of the *nod* regulon, encoding a transcription factor that senses host flavonoids and activates downstream *nod* gene expression [167]. Most rhizobia carry multiple *nodD* copies. NodD of certain broad‐host‐range rhizobium can sense a broader range of flavonoids than NodDs from rhizobia of narrow host range [167]. For example, *Rhizobium tropici* CIAT899, a broad‐host‐range strain, utilizes different NodDs for nodulation on different hosts [168]. In *Mesorhizobium loti*, NodD1 primarily functions in infection threads while NodD2 acts in the rhizosphere and within nodules [169]. In *S. meliloti*, over‐expressed NodD3 can function in the absence of flavonoids. In *S. fredii* and *Bradyrhizobium diazoefficiens*, NodD2 negatively regulates the expression of *nodD1* that encodes the activator of *nod* genes [170, 171, 172]. For those single‐NodD rhizobia, such as *Rhizobium leguminosarum*, NodD autoregulates its own expression [173].

NodD can also activate the transcription of *ttsI*, which encodes the local activator of T3SS and effector genes. The phyletic distribution of T3SS is restricted compared to *nod* genes among rhizobia, the essentiality of T3SS to nodulation can be NF‐dependent or NF‐independent in different rhizobium‐host pairs [126, 127, 174, 175]. Different effectors or polymorphism of the same effector can shape the symbiotic compatibility by either promoting infection in compatible hosts or triggering immune rejection in incompatible hosts [176, 177, 178]. The symbiotic role of T4SS and T6SS and their regulation has been understudied. Nodulation of *Mesorhizobium loti* strains on *Leucaena leucocephala* can be blocked due to the presence of either T3SS or T4SS, while the T4SS mutant shows delayed nodulation on *Lotus corniculatus* [179]. Similarly, T6SS can play either a positive or negative role in nodulation in a rhizobium‐host dependent manner [180, 181, 182, 183, 184]. In *M. loti* harboring T4SS and its effector proteins, the flavonoid‐NodD module is required for activating their expression [185]. In certain *S. fredii* strains harboring T6SS, high levels of c‐di‐GMP and AHLs activate the expression of T6SS [185]. Independent studies on these strains show that the flavonoid‐NodD1 module can enhance certain quorum‐sensing systems and biofilm formation, implying a potential link between symbiotic signal and T6SS expression via AHLs and c‐di‐GMP signaling pathways [161]. In *S. fredii* HH103, the transcription of flagellar gene *flgJ* can be activated by the flavonoid‐NodD1‐TtsI module [140], and its flagellin flg22‐II acts as an immunity elicitor and impairs nodulation efficiency on soybean plants [155]. Notably, over expression of *NodD3* in *S. meliloti*, EPS biosynthesis genes are upregulated [186]. In the *S. meliloti*‐alfalfa symbiosis, succinoglycan (EPS I) is essential for infection thread initiation and elongation, while in *S. fredii*‐soybean interactions, EPS is largely dispensable [187, 188]. This variation reflects the diversity of infection mechanisms and host requirements across the legume family. Rhizobia can produce phytohormones such as indole‐3‐acetic acid (IAA) in response to flavonoids through NodD‐dependent pathways [189, 190]. IAA production facilitates nodule organogenesis and influences infection thread development [191, 192].

After entering nodule cells, rhizobia differentiate into bacteroids, enclosed by plant‐derived membranes to form symbiosomes. In the IRLC legumes and *Aeschynomene*, nodule‐specific cysteine‐rich (NCR) peptides induce terminal bacteroid differentiation [193, 194, 195, 196]. The BacA protein, a peptide transporter, is essential for bacteroid development in galegoid but not phaseoloid legumes [197, 198, 199]. In some *Sinorhizobium* strains, the *hrrP* gene, located on accessory plasmids, encodes a peptidase that breaks down host‐derived NCR peptides, improving symbiont adaptation at the cost of nitrogen fixation efficiency and host growth [200].

Nitrogen fixation is the core function of rhizobial symbiosis, catalyzed by nitrogenase, a highly oxygen‐sensitive enzyme complex. Effective nitrogen fixation requires stable microaerobic conditions, intact nitrogenase structure, and sufficient energy and electron supply, all governed by a conserved set of *nif* and respiratory fixation (*fix*) genes [201]. The *nif* gene cluster encodes the structural and functional components of nitrogenase. *nifHDK* is the core structural gene: *nifH* encodes the iron protein, and *nifDK* encodes the molybdenum‐iron protein, the catalytic core of nitrogenase [202]. *nifENB* genes mediate the synthesis of the iron‐molybdenum cofactor (FeMo‐co), indispensable for nitrogenase activity [203]. Notably, many rhizobia lack homocitrate synthase NifV and get homocitrate from legume hosts to make the functional FeMo‐co [204]. In rhizobia, NifA is the central transcriptional activator of the *nif* regulon under microaerobic conditions [202]. Moreover, *nif* genes are usually clustered with *nod* genes on symbiosis islands or plasmids, facilitating co‐transfer via HGT. Nitrogenase is irreversibly inactivated by oxygen, so rhizobia rely on the *cbb3*‐type high‐affinity terminal oxidase and leghemoglobins to maintain low‐oxygen environments in nodules [205, 206, 207, 208, 209, 210, 211]. The *fix* gene cluster directing the assembly and function of *cbb3* terminal oxidase is transcriptionally activated by the FixL‐FixJ‐FixK module in *S. meliloti*, *Azorhizobium caulinodans*, and *B. diazoefficiens*, and by the hFixL‐FxkR‐FixKf‐FnrN module in *Rhizobium etli* and *R. leguminosarum* [206, 212, 213, 214, 215]. This regulatory diversity highlights lineage‐specific adaptations while maintaining the core function of oxygen‐responsive gene expression.

Nitrogen fixation is an energetically expensive reaction. Dicarboxylate transporters (*DctA/DctB/DctD*) import host‐derived organic acids, the primary carbon source for bacteroids [216, 217]. This is consistent with heavy reliance on dicarboxylates by bacteroids as revealed in a global metabolic flux analysis of rhizobia [218, 219]. The electron bifurcating complex FixABCX provides low‐potential electrons to reduce ferredoxin (FdxN) that, in turn, maintains electron supply to nitrogenase [201, 220, 221, 222]. Ion transporter genes (zinc, iron, molybdenum, sulfate, phosphorus transporters) uptake essential metal cofactors and nutrients, imbalance of which directly reduces nitrogenase activity [223, 224, 225, 226, 227, 228, 229]. Although the accumulation of polyhydroxybutyrate (PHB) granules is rarely observed in terminally differentiated bacteroids (such as those in alfalfa and pea nodule cells) versus in undifferentiated bacteroids (those in soybean and common bean nodule cells), the PHB synthesis and degradation genes are actively expressed in both bacteroids types, indicating dynamic modulation of reducing power and carbon metabolism [230, 231]. Bacteroids export fixed nitrogen primarily as ammonia, which is assimilated by the plant into amino acids. Some of these amino acids, particularly branched‐chain amino acids, are supplied back to bacteroids, which repress their own branched‐chain amino acid biosynthesis [232, 233].

#### Evolutionary and regulatory backdrop of rhizobial niche adaptation

The wide distribution of rhizobia and their distinct facultative endosymbiotic life cycle stem from their plastic and open pan‐genomes [117, 234]. Core genes maintain basic cellular functions, while accessory genes (acquired via HGT, gene duplication, or mutation) confer niche adaptability [235, 236]. Symbiotic islands or plasmids, carrying core symbiosis genes, are mobile genetic elements that facilitate rapid spread of symbiotic traits [237, 238, 239, 240]. Many rhizobia carry accessory plasmids encoding stress resistance, nutrient uptake, and antagonistic functions, enabling survival in fluctuating soil conditions and during symbiotic interactions with diverse legume hosts [115]. Transposable elements (TEs) and insertion sequences (ISs) are enriched in symbiosis regions, driving genome rearrangement, gene loss, and mutation [241, 242]. These genetic variations allow rhizobia to quickly adapt to new soil environments and host plants [241, 242, 243]. Moreover, transient hypermutability mediated by the mutagenesis *imuABC* cassette accelerates adaptive evolution, helping rhizobia overcome environmental stresses and establish stable populations in diverse habitats [244].

The entire symbiotic process, from soil survival and root colonization through infection to nitrogen fixation, is governed by a multilayered, dynamic regulatory network. This network integrates environmental signals, host cues, and intracellular metabolic status, coordinating the expression of core symbiosis genes, accessory genes, and core metabolic genes. In addition to the abovementioned pathway‐specific regulators, an emerging integrated global and local regulation network involving the zinc‐finger protein MucR has been recently uncovered. MucR/RosR is a conserved global regulator in α‐proteobacteria and preferentially binds AT‐rich symbiosis islands and plasmids, generally repressing AT‐rich genes and facilitating the transposition of insertion sequences into conditionally deleterious genes [243, 245, 246]. It regulates hundreds of target genes encoding NodD, TtsI, T3SS, and its effector proteins, ion transporters, and those proteins involved in chemotaxis, motility, biofilm formation, and stress response [224, 245, 246]. Among a few well‐characterized targets, MucR globally down‐regulates the transcription of genes encoding diguanylate cyclases that produce c‐di‐GMP [240]. On the other hand, c‐di‐GMP can act as an anti‐silencing factor for certain target genes (e.g., those involved in the biosynthesis of cryptic surface polysaccharides) repressed by MucR [247]. However, the anti‐silencing mechanisms for most MucR targets that play important roles in niche adaptation remain unknown.

#### Section summary

Rhizobial symbiosis is a complex trait shaped by long‐term adaptive evolution, relying on strain diversity, functional gene modules, and dynamic regulatory networks. HGT of core *nod* and *nif* genes initiates symbiotic potential, while genome innovation (e.g., gene gain, loss, rearrangement) and regulatory rewiring determine symbiotic efficiency. Rhizosphere colonization, host infection, and nitrogen fixation are sequentially controlled by dedicated gene sets, coordinated by global and specific regulators. Current research has clarified most core symbiosis genes and key regulatory pathways, but gaps remain: the anti‐silencing mechanisms of the global regulator MucR, the full spectrum of lineage‐specific regulators, and the molecular basis of strain‐host specificity are not fully understood. Future studies should combine pan‐genomics, experimental evolution, and systems biology to dissect the entire symbiotic regulatory network. This progress provides a solid foundation for agricultural applications. By modifying core symbiosis genes, optimizing regulatory circuits, and enhancing stress resistance via synthetic biology, we can develop elite rhizobial inoculants with high competitiveness, broad adaptability, and superior nitrogen fixation efficiency. These engineered inoculants will reduce chemical fertilizer use, promote soil health, and advance sustainable agricultural production globally.

### Mycorrhizas: The ancient symbioses of terrestrial plants

While rhizobia represent a paradigm of bacterial endosymbiosis largely restricted to legumes, a much more ancient and widespread form of plant‐fungal mutualism is the mycorrhizal symbiosis. Here, we explore the evolutionary history, diversity, and molecular underpinnings of this critical interkingdom interaction.

#### Mycorrhizal fungi and mycorrhizal symbioses

Mycorrhizas, derived from the Greek for “fungus‐root,” represent one of the ancient, ubiquitous, and ecologically significant biological interkingdom interactions on earth [248]. This symbiosis involves an estimated 340,000 land plant species and approximately 50,000 taxa of soil fungi, forming a partnership that has shaped the evolution of terrestrial ecosystems for over 400 million years [248, 249]. This association is defined by reciprocal nutrient exchange: host plants supply fungal partners with carbon (C) derived from photosynthesis (including lipids and sugars), in return, fungi deliver essential mineral nutrients, particularly phosphorus (P) and nitrogen (N), and water from the soil to the plant [249, 250, 251]. Beyond nutrient exchange, mycorrhizas also enhance tolerance to biotic and abiotic stresses, improve soil structure, and shape the composition of plant and microbial communities [248, 252].

Over more than 150 years of research, four principal mycorrhizal types have been characterized based on their distinct morphology, fungal and plant taxonomy, and ecological niches: arbuscular mycorrhizas (AM), ectomycorrhizas (ECM), orchid mycorrhizas (ORM), and ericoid mycorrhizas (ERM) [248, 249]. AM symbiosis, formed by fungi of the subphylum Glomeromycotina, is ancient and widespread, associating with approximately 72% of land plants [248, 253]. In contrast, ECM symbiosis involves a diverse array of Basidiomycota and Ascomycota fungi that associate primarily with woody trees and shrubs, dominating boreal and temperate forest ecosystems [249, 253]. ORM and ERM are more specialized, forming with members of the Orchidaceae and Ericaceae families, respectively, and often involve fungi with significant saprotrophic capabilities [248, 254]. More recently, Mucoromycotina “fine root endophytes” (MFRE) have been established as a distinct mycorrhizal association [255]. Elucidating the evolution, molecular cell biology, and adaptations of mycorrhizal symbioses is crucial for translating this fundamental knowledge into sustainable agriculture.

#### Evolution of mycorrhizal symbioses

The evolutionary history of mycorrhizal symbioses reflects ancient origins, convergent evolution, and genomic adaptation. The earliest direct fossil evidence comes from the 407‐million‐year‐old Rhynie chert in Scotland, a hot‐spring deposit that preserved early terrestrial ecosystems with exceptional cellular detail [55]. Within these fossils, plants such as *Aglaophyton majus* and *Horneophyton lignieri* show clear evidence of intracellular fungal colonization [256, 257]. In *Aglaophyton*, arbuscule‐like structures in cortical cells provide the first unequivocal evidence of AM‐like associations in early land plants [252, 256]. These early associations were likely crucial for plant terrestrialization, as the fungi would have aided water and nutrient acquisition from harsh, nutrient‐poor primordial soils [55, 258].

Phylogenomic evidence dates the origin of both Glomeromycotina (the AM fungi) and Mucoromycotina (which form coil‐forming mycorrhizas) to the mid‐to‐late Silurian period, around 420 million years ago [55, 259]. Intriguingly, the Rhynie chert also reveals evidence of dual colonization, with *Horneophyton* harboring both Glomeromycotina and Mucoromycotina fungi in different parts of its plant body, indicating that early plants may have engaged with multiple fungal partners concurrently [256, 257]. This supports the view that ancestral land plants were predisposed to form symbiotic associations with a broader range of fungi than previously thought [258, 260]. The genomes of AM fungi reflect this ancient, obligate biotrophic lifestyle: they are large, rich in transposable elements, and lack genes for de novo fatty acid biosynthesis, rendering them fatty acid auxotrophs entirely dependent on their plant hosts for these essential carbon compounds [59, 253]. Moreover, they have lost many genes encoding plant cell wall‐degrading enzymes (CAZymes), limiting their saprotrophic capacity and potentially helping to avoid triggering host immune responses [55, 261].

ECM symbiosis evolved much later, likely coinciding with the rise of woody plants such as Pinaceae (gymnosperms) between 170 and 270 million years ago [55, 256]. However, its fossil record remains sparse, with the oldest well‐documented specimens dating to the early Eocene (52 million years ago) [252, 256]. Unlike AM fungi, ECM symbiosis has evolved independently multiple times, an estimated 78 to 82 times, from saprotrophic ancestors within the Basidiomycota and Ascomycota [55, 262, 263]. This convergent evolution has left a striking genomic signature: the repeated loss of genes encoding plant cell wall‐degrading enzymes, particularly those involved in lignin and cellulose degradation [261, 262]. For example, ECM fungi derived from white‐rot ancestors have lost many class II peroxidases and cellulases while retaining laccases and other enzymes that may enable nitrogen acquisition from soil organic matter [262, 264]. This transition from a free‐living saprotrophic to a mutualistic biotrophic lifestyle is a key evolutionary event that enabled ECM fungi to dominate in nutrient‐limited temperate and boreal forests [54].

In contrast to AM and ECM fungi, the genomes of ORM and ERM fungi tell a different evolutionary story. These fungi, which include members of the Basidiomycota (for ORM) and Ascomycota (for ERM), have retained a large and diverse set of CAZymes, along with many proteases and lipases, much like their saprotrophic ancestors [55, 254, 265]. This genomic feature reflects their dual ecological lifestyle. During the early and heterotrophic stages of orchid development, ORM fungi obtain carbohydrates by decomposing soil organic matter, effectively acting as saprotrophs to provide carbon to the developing seedling [55, 266]. Similarly, ERM fungi inhabit nutrient‐poor, acidic soils and use their enzymatic toolkit to mobilize organic nutrients, a function essential for their ericaceous hosts [254, 265]. Therefore, while the shift to a mycorrhizal lifestyle in AM and ECM fungi involved a major reduction in saprotrophic capability, ORM and ERM fungi represent a more recent evolutionary transition in which ancestral saprotrophic abilities have been retained and co‑opted for symbiosis [55, 254].

#### Unique and common traits across mycorrhizal types

Despite their shared mutualistic nature, the four mycorrhizal types exhibit a compelling synthesis of common features and unique adaptations that reflect their distinct evolutionary histories and ecological strategies [55, 254, 262].

AM and ECM fungi share several common traits. One is the convergent loss of many plant cell wall‑degrading enzymes (CAZymes), which limits their saprotrophic capacity and helps prevent the release of cell wall fragments that would otherwise trigger plant defense responses [55, 261, 262]. Another is the expansion of gene families encoding small secreted proteins (SSPs) and effectors [55, 262, 267]. Both AM fungi (e.g., *R. irregularis*) and ECM fungi (e.g., *Laccaria bicolor*) harbor large repertoires of these proteins, thought to be essential for manipulating host cellular processes, suppressing immunity, and establishing functional symbiosis [267, 268, 269, 270]. For example, the AM effector SP7 localizes to the plant nucleus and interacts with a pathogenesis‐related transcription factor ethylene responsive factor 19 (ERF19) to dampen defense responses, whereas the ECM effector MiSSP7 enters plant cells and interferes with jasmonic acid (JA) signaling [268, 269].

AM fungi possess unique adaptations in their nutritional strategies and genomic repertoires. As obligate biotrophs with a narrow ecological niche, their genomes have undergone extreme specialization, including the loss of key metabolic pathways such as thiamine and fatty acid biosynthesis, making them entirely dependent on their plant hosts [55, 253, 271]. Consequently, they are largely incapable of decomposing complex organic matter and instead scavenge nutrients that have been mineralized by other microbes [59, 272].

In contrast, many ECM fungi, and particularly ORM and ERM fungi, have retained a broader ecological niche and significant saprotrophic capabilities [55, 254, 262]. Their genomes encode diverse CAZymes, proteases, and lipases, enabling decomposition of complex soil organic polymers [254, 262, 265]. For ECM fungi, this capacity is crucial for mobilizing nitrogen from recalcitrant organic matter, the primary growth‑limiting nutrient in these ecosystems [264, 273]. For ORM fungi, this saprotrophic capacity is essential for supporting the early, heterotrophic stages of orchid development [55, 266]. Therefore, unlike the highly specialized AM fungi, ECM, ORM, and ERM fungi exhibit the metabolic versatility of the mycorrhizal lifestyle, often switching between saprotrophic and mutualistic modes [55, 254, 262].

#### Mycorrhizal symbiosis in plant growth and fitness

**FIGURE 5:**
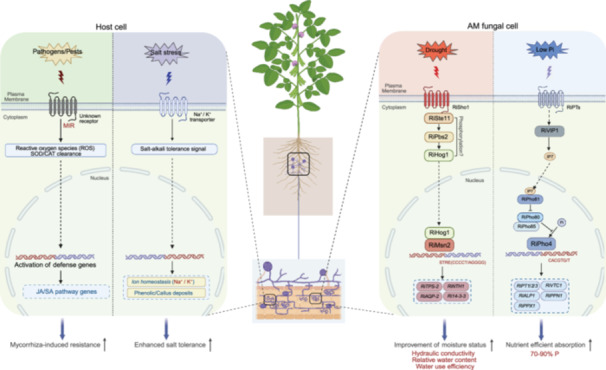
Arbuscular mycorrhizal (AM) symbiosis enhances plant nutrition and stress tolerance. Under phosphorus limitation, the AM fungal transcription factor RiPho4 activates Pi transporters (RiPTs), phosphatases, and polyphosphate metabolism genes to maintain arbuscule development and phosphate homeostasis. Under drought stress, the RiSho1‐HOG1‐MAPK cascade (including RiSte11, RiPbs2, and RiHog1) leads to RiMsn2‐mediated upregulation of stress‐responsive genes (e.g., aquaporins and trehalose synthase), improving water balance and reducing oxidative damage. Under salt stress, AM fungi regulate Na^+^/K^+^ transporters to maintain ion homeostasis. Additionally, mycorrhiza‐induced resistance (MIR) primes plant immune responses via jasmonic acid (JA) and salicylic acid (SA) signaling pathways, protecting against various pathogens and pests. Therefore, these AM‐mediated mechanisms promote plant growth, nutrient acquisition, and resilience to abiotic and biotic stresses. Abbreviations: MIR, mycorrhiza‐induced resistance; JA, jasmonic acid; SA, salicylic acid; Sho1, synthetic high osmolarity sensitive 1; Ste11, protein kinase sterile 11; Pbs2, MAP kinase kinase PBS2; Hog1, high osmolarity glycerol response 1; Msn2, multicopy suppressor of SNF1 (sucrose non‐fermenting 1 protein kinase) protein 2; STRE, stress‐responsive element; TPS‐2, trehalose‐6‐phosphate synthase 2; NTH1, neutral trehalase 1; AQP‐2, aquaporin 2; 14‐3‐3, 14‑3‑3 protein (derived from its elution fraction numbers in DEAE‑cellulose chromatography, fraction 14, segment 3.3); Pi, inorganic phosphate; PTs, phosphate transporters (e.g., RiPT1/2/3); VIP1, inositol polyphosphate kinase 1; IP7, inositol heptakisphosphate; Pho81, cyclin‐dependent kinase inhibitor; Pho80, phosphate system cyclin; Pho4, phosphate‐sensing transcription factor; VTC1, vacuolar transporter chaperone 1; ALP1, alkaline phosphatase 1; PPN1, Endopolyphosphatase; PPX1, Exopolyphosphatase.

The primary function of mycorrhizal symbiosis is to enhance plant nutrition, with the mycorrhizal pathway accounting for 70%−90% of a plant's phosphate and 21%−75% of its nitrogen [54, 274]. This capacity dramatically improves plant growth and productivity, particularly in nutrient‑poor soils [2, 29, 54, 275]. From a mechanistic perspective, under phosphorus starvation, the transcriptional activator RiPho4 in AM fungi is significantly upregulated and translocates to the nucleus, where it binds to CACGTG/T *cis*‐elements to activate downstream phosphate signaling (PHO) pathway genes, including phosphate transporter‐encoding genes (e.g., *RiPT1/2/3*), phosphatase‐encoding genes (e.g., *RiALP1* and *RiACP1*), and poly‐P metabolism genes (e.g., *RiPPX1* and *RiPPN1*) [123, 276]. This regulatory hub maintains arbuscule development and phosphate homeostasis during symbiosis (Figure 5). Genome‐wide analysis further revealed that at least 72 conserved components of four nutrient‐sensing pathways, cAMP‐PKA (cAMP‐Dependent Protein Kinase A), SNF1 (Sucrose Non‐Fermenting 1), TOR (Target of Rapamycin), and PHO, coordinate AM fungal responses to external and internal nutrient status, optimizing Pi and nitrogen acquisition [277]. Meanwhile, AM fungi acquire ammonium via GintAMT1/3 and nitrate via putative transporters, with nitrogen assimilated into arginine via GS‐GOGAT (Glutamine Synthetase‐Glutamate Oxoglutarate Aminotransferase) and transferred to host roots [278]. This integrated signaling network ensures efficient phosphate and nitrogen delivery to the host, even under limiting conditions.

In addition to this nutritional benefit, mycorrhizal associations confer a wide range of stress tolerance traits, reinforcing their role as key mediators of plant adaptation to challenging environments [279, 280]. Under drought stress, *R. irregularis* activates the plasma membrane receptor RiSho1, initiating a conserved HOG1‐MAPK (High Osmolarity Glycerol 1‐ Mitogen‑Activated Protein Kinase) cascade (e.g., RiSte11, RiPbs2, RiHog1) [281, 282]. Activated RiHog1 interacts with and phosphorylates the transcription factor RiMsn2, enabling its nuclear translocation [283]. RiMsn2 then binds to stress response elements (STREs) in the promoters of downstream genes, including *signaling regulator* (*14‐3‐3*), *aquaporins* (*AQPs*), *trehalose synthase* (*TPS*), and *neutral trehalase* (*NTH1*) [284, 285]. Upregulation of these genes enhances osmotic adjustment, water transport, and arbuscule formation (Figure 5). Consequently, AM fungal colonization improves host plant water status by increasing hydraulic conductivity, relative water content, and water‐use efficiency [283, 286], accompanied by elevated accumulation of soluble sugars and proline that protect cells from dehydration [279, 286]. AM fungi also mitigate oxidative damage under drought and salinity by boosting antioxidant enzyme activities (e.g., superoxide dismutase and catalase), thereby reducing reactive oxygen species (ROS) accumulation [280, 287]. Together, these molecular and physiological responses collectively enhance host plant drought tolerance (Figure 5). Under salt stress, AM fungal colonization contributes to ion homeostasis by restricting root‑to‑shoot translocation of sodium (Na^+^) while enhancing the uptake and transport of potassium (K^+^), a key determinant of the Na^+^/K^+^ ratio [286, 288]. In addition, AM symbiosis confers tolerance to heavy metal toxicity, primarily through immobilization of metals within the soil or fungal structures, thereby limiting their translocation to plant shoots [279, 289].

Mycorrhizal fungi also play a significant role in protecting their host plants against a range of biotic threats (Figure 5), a phenomenon known as mycorrhiza‐induced resistance (MIR) [290]. MIR is a systemic response that primes the plant's immune system, allowing for a faster and stronger defense activation upon pathogen attack [290]. This enhanced resistance is mediated by the activation of JA and SA signaling pathways and the priming of defense‐related genes [290, 291, 292]. For example, AM fungal colonization in tomato leads to increased synthesis of phenols and callose deposition, which can restrict the growth of fungal pathogens [290, 292]. The induced resistance is effective against a variety of attackers, including fungal and bacterial pathogens, nematodes, and even herbivorous insects [279, 290, 293]. This dual role of mycorrhizas as both biofertilizers and bioprotectants establishes them as powerful tools for sustainable agriculture.

#### Mycorrhizosphere and beyond

Mycorrhizal fungi exert ecological effects far beyond the root‐fungus interface. Their extraradical hyphae form extensive soil networks that connect multiple plants, often across species, into a “common mycorrhizal network” (CMN), which shuttles nutrients, water, and signals, thereby influencing plant interactions, community dynamics, and ecosystem resilience [54, 294, 295].

The hyphae themselves create a specialized niche, the hyphosphere, where AM fungal exudates, including sugars, amino acids, and carboxylates, attract distinct bacterial communities from bulk soil [57, 59, 296]. These bacteria form a functional tripartite symbiosis with the plant and fungus [59, 297]. Stable isotope labeling shows that AM fungi rapidly transfer plant‐derived carbon to these bacteria, fueling their growth [59, 298]. In return, the bacteria mineralize complex organic nutrients that AM fungi, with their limited saprotrophic capacity, cannot access. For example, phosphate‐solubilizing *Rahnella aquatilis* uses hyphal fructose to upregulate phosphatase genes, converting organic phosphorus into inorganic phosphate for fungal uptake and transfer to the plant [59, 299]. This mutualistic carbon‐for‐nutrient exchange drives terrestrial nutrient cycling [59, 297]. Different AM fungi recruit distinct bacterial communities, but taxa such as Myxococcales and Betaproteobacteriales are consistently enriched across species and soil types, indicating a stable core microbiome with key functional roles [58, 296]. Therefore, mycorrhizal symbiosis is conceptualized as a plant‐AM fungus‐bacterium continuum that drives essential ecosystem processes [59, 297].

#### Establishment of mycorrhizal symbiosis

A successful mycorrhizal association begins long before physical contact, through a complex molecular dialogue in the rhizosphere [248, 260]. This pre‑symbiotic phase is triggered by the host plant in response to nutrient deficiency, particularly low phosphorus (P) and nitrogen (N) availability [260, 279, 300].

For AM fungi, the key plant‐derived signals are strigolactones (SLs). Under nutrient limitation, plants upregulate SL biosynthesis and exude SLs via the ATP‐binding cassette subfamily G (ABCG) transporters [52, 53, 54, 301, 302, 303]. SLs stimulate fungal spore germination, hyphal branching, and mitochondrial activity, enhancing host root location [303, 304]. In response, AM fungi produce Myc factors (e.g., short‐chain chitooligosaccharides CO4/5 and lipochitooligosaccharides LCOs), which are perceived by the host to activate the common symbiosis signaling pathway (CSSP) [248, 253, 304, 305, 306, 307].

ECM symbiosis involves a parallel molecular dialogue. Although SLs are not the primary plant signal for most ECM fungi, the model ECM fungus *L. bicolor* produces diverse sulfated and non‐sulfated LCOs, similar to AM Myc‐LCOs and rhizobial Nod factors [267]. These LCOs trigger root hair branching in legumes and, crucially, induce nuclear calcium spiking in *Populus* roots in a CSSP‐dependent manner, indicating that the CSSP has been co‐opted for ECM symbiosis in some plant lineages [267, 308]. For ORM and ERM, the exchanged signals remain less characterized, but CSSP conservation in orchids and ericaceous plants suggests similar mechanisms [260, 309].

#### Mycorrhizal symbiotic interface: A hub for nutrient exchange

Upon successful signaling and contact, the fungus colonizes root tissue to establish the nutrient exchange interface, involving extensive cellular remodeling [54, 310, 311]. In AM, the fungus forms a hyphopodium on the epidermis, penetrates via a host‐derived pre‐penetration apparatus, and branches into tree‐like arbuscules in cortical cells [310, 312]. In ECM, hyphae form a mantle on the root surface and grow intercellularly to produce the Hartig net, which maximizes apoplastic contact without cell penetration [54, 267]. In ERM and ORM, dense intracellular hyphal coils form in epidermal or cortical cells, surrounded by the host membrane [54, 55].

A common feature of mycorrhizal associations is the apoplastic nature of fungal hyphae, which are separated from host cytoplasm by specialized plant‑derived membranes: the periarbuscular membrane (PAM) in AM, the perifungal membrane in ERM and ORM, and the plant membrane adjacent to the Hartig net in ECM [54, 55, 310, 313]. As the primary site for bidirectional nutrient exchange, this interface orchestrates a tightly regulated flow of carbon, phosphorus, and nitrogen between the two symbiotic partners [54, 310].

#### Cross‐kingdom communications regulate mycorrhizal symbiosis

**FIGURE 6:**
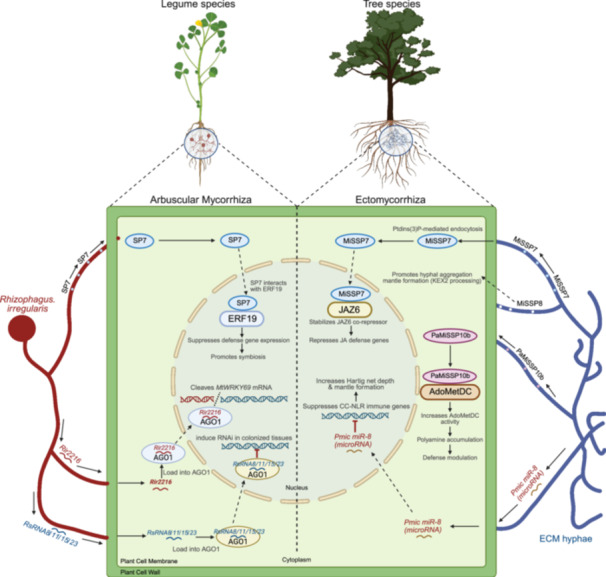
Cross‐kingdom communications in mycorrhizal symbiosis: Effector proteins and small RNAs. Mycorrhizal fungi (AM fungus: *Rhizophagus irregularis*; ECM fungi: *Laccaria bicolor*, *Pisolithus microcarpus*) employ a dual strategy of secreted effectors and small RNAs (sRNAs) to modulate host plant cellular programs. Upper panel (Effectors): The AM fungal effector SP7 translocates to the plant nucleus and interacts with ERF19 to suppress defense gene expression. ECM effectors include MiSSP7, which enters the nucleus and stabilizes JAZ6 to repress jasmonic acid (JA) signaling; MiSSP8, which promotes hyphal aggregation; and PaMiSSP10b, which interacts with AdoMetDC in the cytoplasm to increase polyamine levels, modulating defense. Lower panel (Cross‐kingdom RNAi): AM fungus‐derived sRNAs (e.g., Rir2216, RsRNA8, RsRNA11, RsRNA15, and RsRNA23) are transferred into host cells, load into AGO1, and cleave target mRNAs such as MtWRKY69, suppressing colonization resistance. ECM fungus‐derived Pmic_miR‐8 silences host CC‐NLR immune genes, enhancing Hartig net formation. Collectively, these molecular tools enable mycorrhizal fungi to establish mutualistic symbiosis by precisely suppressing plant immunity at multiple levels (e.g., transcriptional, post‐transcriptional, and metabolic). AGO1, argonaute 1; AdoMetDC, S‐adenosyl methionine decarboxylase; AM, arbuscular mycorrhiza; CC‐NLR, coil‐coil nucleotide‐binding leucine‐rich repeat receptor; ECM, ectomycorrhiza; ERF19, ethylene response factor 19; JAZ6, jasmonate ZIM‐domain protein 6; MiSSP7, mycorrhiza‐induced small secreted protein 7; SP7, small secreted protein 7.

The successful colonization of plant roots by mycorrhizal fungi depends on their ability to finely manipulate host cellular programs, particularly through the suppression of plant immune responses. In addition to well‑characterized signaling pathways, fungal‑secreted effector proteins and cross‑kingdom small RNAs have emerged as pivotal molecular toolkits in this dialogue (Figure 6).

#### Effector proteins

Mycorrhizal fungi employ diverse small secreted proteins (SSPs) as effectors that act within or between plant cells to facilitate symbiosis (Figure 6). The first characterized AM fungal effector SP7 from *R. irregularis* translocates to the plant nucleus and interacts with the pathogenesis‐related transcription factor ERF19 to dampen defense responses and promote symbiosis [268]. Similarly, the ECM fungal effector MiSSP7 from *L. bicolor* is secreted upon host perception, enters plant cells via phosphatidylinositol 3‐phosphate‐mediated endocytosis, and localizes to the nucleus, where it stabilizes the JA co‐repressor JAZ6 (Jasmonate ZIM‐domain protein 6) to repress defense genes and facilitate colonization [269, 314]. Another *L. bicolor* effector, MiSSP8, promotes hyphal aggregation and mantle formation, with its C‐terminal repeats likely processed by KEX2 (Killer expression protease 2) protease to release peptides that regulate early symbiotic structures [315]. In *Pisolithus microcarpus*, the effector PaMiSSP10b enters host cytoplasm and interacts with S‐adenosyl methionine decarboxylase (AdoMetDC) in *Eucalyptus grandis* roots, enhancing its activity and increasing polyamine accumulation, a defense modulator that favors mycorrhizal colonization [316]. Together, these lines of evidence illustrate the diversity of host pathways targeted by effectors, from nuclear signaling to cytoplasmic metabolism, reflecting the sophisticated strategies mycorrhizal fungi have evolved to establish mutualism.

#### Cross‐kingdom RNA interference

Beyond effectors, recent discoveries have identified small RNAs (sRNAs) as critical cross‑kingdom signaling molecules in mycorrhizal symbiosis (Figure 6). Cross‑kingdom RNA interference (ckRNAi) refers to RNAi triggered by sRNAs from one organism in other kingdoms. In ECM, a microRNA from *P. microcarpus*, *Pmic_miR‑8*, is induced during colonization and transferred into host cells. Synthetic *Pmic_miR‑8* increases Hartig net depth, while its inhibition reduces Hartig net depth and mantle formation, and it suppresses host CC‑NLR (Coiled‑Coil Nucleotide‑binding Leucine‑rich Repeat receptor) immune genes, indicating that ckRNAi promotes ECM symbiosis by dampening immunity [317]. In AM, *R. irregularis*‑derived sRNA *Rir2216* loads into *M. truncatula* AGO1 (ARGONAUTE 1) and cleaves *MtWRKY69*; overexpression of *MtWRKY69* suppresses colonization, indicating the AM fungus uses ckRNAi to promote symbiosis [318]. Furthermore, four *R. irregularis* sRNAs (*RsRNA8/11/15/23*) loaded into *L. japonicus* AGO1 induces RNAi in colonized tissues, and sequestering them reduces colonization, confirming ckRNAi as a regulator of AM symbiosis [319]. Whether plant‑derived sRNAs similarly silence fungal genes remains unresolved. Host‑induced gene silencing (HIGS) has been shown to silence AM fungal genes via artificially expressed plant sRNAs [320], and extracellular vesicles in the periarbuscular space may mediate such transfer [313, 321].

In summary, mycorrhizal fungi employ a dual strategy, consisting of secreted effector proteins and functional small RNAs, to precisely modulate host immunity and metabolism across transcriptional, translational, and post‑transcriptional levels. Elucidating these mechanisms not only reveals striking convergent evolution between mutualistic and pathogenic microbes but also opens new avenues for harnessing these molecular tools to enhance symbiotic efficiency in crops and to promote sustainable agriculture.

#### Unlocking AM fungal biology: From imaging to genetic manipulation

AM symbiosis involves continuous rounds of root invasion and asynchronous colonization, with the fungus proliferating both inside and outside the root. AM fungi grow as coenocytic (multinucleate, aseptate hyphae) cells with bidirectionally moving nuclei, making it difficult to define cell types based solely on morphology [322, 323].

Single‑nucleus RNA sequencing (snRNA‑seq) and spatial transcriptomics offer unprecedented resolution of developmental and molecular dynamics in AM symbiosis [324, 325]. Serrano et al. applied snRNA‑seq to *M. truncatula* roots colonized by *R. irregularis*, but recovered only plant nuclei, highlighting the need for optimized isolation of fungal nuclei [326]. Recent advances in cell type‑specific and imaging‑based spatial transcriptomics have achieved nanometer‑scale resolution, enabling visualization of gene expression in fungal structures within rice roots [327]. Establishing molecular markers for distinct AM fungal “cell types” across lineages will be a critical step toward understanding the functional roles of their diverse morphologies [320].

Biophysical approaches are also advancing our understanding of fungal foraging and resource transport. Robotic imaging and mathematical modeling have revealed pulse‑like exploration modes in AM mycelium development [328, 329]. Imaging tools such as AMSlide, AMF‑SporeChip, colonization reporters, and transparent soil systems allow AM fungi to be visualized live and undisturbed [330, 331, 332, 333]. Using membrane lipid biosensors, Guyon et al. showed that AM fungi and oomycetes display distinct lipid signatures at plant interfaces, suggesting that specific membrane compositions may distinguish mutualistic from pathogenic interactions [334].

Genetic engineering holds great promise for deciphering AM fungal biology, though stable transformation remains elusive. Reverse genetics via RNAi has relied on transgenic plants or viruses to express dsRNA [335, 336], but Fan et al. (2025) demonstrated that direct spore soaking with ~200‑bp dsRNA enables efficient gene‑specific knockdown without transgenesis [337]. To date, at least 15 AM fungal genes have been functionally characterized using RNAi. However, a recent preprint by Dallaire et al. reveals that Glo‐meraceae species lack several DNA polymerases essential for eukaryotic replication, posing fundamental challenges for classical transformation approaches [320].

Taken together, high‑resolution imaging and targeted genetic manipulation are converging to illuminate the cellular and molecular principles that govern AM fungal biology, opening new avenues for biotechnological application.

### Plant‐arbuscular mycorrhizal fungus‐bacterium continuum

#### The hyphosphere and the plant‐AM fungus‐bacterium continuum

The hyphosphere represents a specialized microhabitat defined by the thin layer of soil (approximately 2 mm) surrounding the extraradical hyphae (ERH) of AM fungi, which is distinct from bulk soil because its physical, chemical, and biochemical properties are reshaped by hyphal exudates [296]. These exudates create a high‐energy niche that not only supports a diverse of microbiota but also serves as a biological “highway”, facilitating the dispersal of bacteria through the complex soil matrix [338, 339].

The interactions within this zone are characterized by the functional synergy between the fungi and hyphospheric bacteria. Specific bacterial genera, including *Bacillus* and *Streptomyces*, act as “mycorrhizal helper bacteria” by actively promoting hyphal elongation, branching, and overall colonization efficiency in the host roots [340, 341]. Beyond structural support, these bacteria perform a critical metabolic service by mineralizing and releasing otherwise inaccessible P, N, and essential micronutrients [342, 343]. By bridging the gap between immobile nutrients and the fungal network, these bacteria influence the broader biogeochemical cycling of terrestrial ecosystems, offering significant potential for enhancing agricultural productivity and resilience in the face of a changing climate.

These complex biological layers converge into the plant‐AM fungus‐bacterium (PFB) continuum, a tripartite system that links atmospheric C fixation to soil mineral cycles [59]. This continuum is anchored by two primary exchange interfaces: the peri‐arbuscular space (PAS) and the hyphosphere interface. Inside the root cortical cells, the PAS facilitates the internal trade where the plant provides C to the fungus in exchange for minerals. Simultaneously, in the soil micropores, the hyphosphere serves as the external interface where the fungus exchanges plant‐derived C for bacterial‐sourced nutrients [59]. This biological bridge allows for the efficient bidirectional flow of energy and matter, transforming the soil‐root interface into a highly integrated and dynamic ecosystem.

#### Carbon flows in the plant‐AM fungus‐bacterium continuum

**FIGURE 7:**
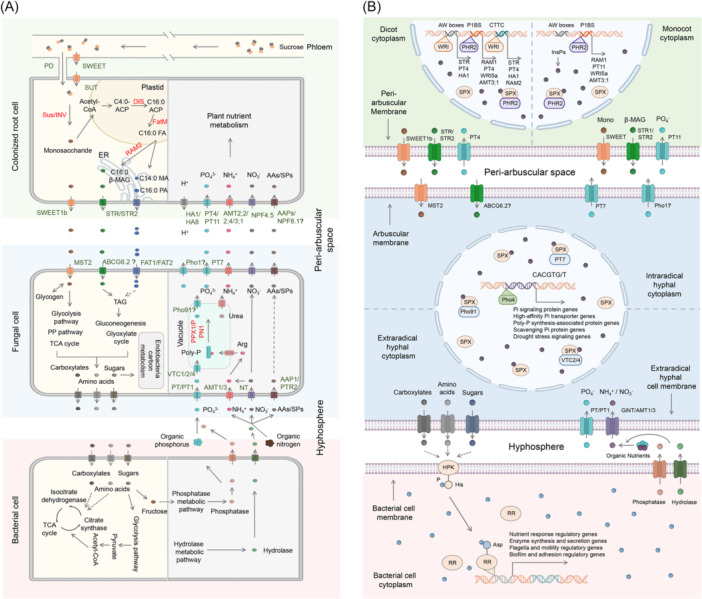
Cross‐kingdom nutrient exchange in the plant‐arbuscular mycorrhizal fungus‐bacterium continuum. (A) Carbon and mineral flows along the plant‐arbuscular mycorrhizal (AM) fungus‐bacterium continuum. Sucrose synthesized in leaves is unloaded into root cells and hydrolyzed to monosaccharides, which are used to synthesize fatty acids (e.g., myristic acid, palmitic acid, and β‐monoacylglycerols). Plant‐derived fatty acids and monosaccharides are transferred across the peri‐arbuscular and fungal membranes into AM fungi. There they are metabolized through the glyoxylate cycle and gluconeogenesis, providing carbon and energy for endobacteria and hyphospheric bacteria. Fructose acts as a signal to induce bacterial phosphatase pathways, accelerating organic phosphorus mineralization, while hydrolases convert organic nitrogen to inorganic forms. AM fungi take up these nutrients, transport them to arbuscules, and deliver them into root cells. Solid lines indicate experimentally verified pathways; dotted lines denote unverified steps. Transporters with a question mark lack functional validation. (B) Molecular mechanisms governing carbon and mineral exchange along the plant‐AM fungus‐bacterium continuum. In plants, monocots and dicots employ distinct regulatory modules for carbon‐mineral exchange. Both possess the SPX‐PHR2 module: PHR2 binds P1BS *cis*‐elements to regulate phosphate‐starvation and symbiosis genes (e.g., *RAM1*, *PT11*, *WRI5a*, and *AMT3;1*). In dicots, the WRI pathway induces bidirectional C‐Pi exchange and symbiosis genes by binding CTTC/AW motifs. Within AM fungi, the transcriptional factor Pho4 controls Pi homeostasis genes via CACGTG/T elements. Bacteria sense external signals through two‐component systems, where a histidine protein kinase (HPK) phosphorylates a response regulator (RR) to modulate target gene expression. AA, amino acid; ABCG, ATP‐binding cassette G‐type transporter; ACP, acyl carrier protein; AMT, ammonium transporter; DIS, β‐ketoacyl‐ACP synthase I; FatM, acyl‐ACP thioesterase; FAT, fatty acid transporter; HA, H^+^‐ATPase; INV, invertase; MST2, monosaccharide transporter 2; NT, nitrate transporter; NPF, nitrate transporter 1/peptide transporter family; PHO1, PHO1‐type phosphate transporter; PPX/PPN, exopolyphosphatase; PT, PHT1 family phosphate transporter; PTR/NPF, peptide/nitrate transporter; RAM2, glycerol‐3‐phosphate acyltransferase; STR, half‐size ABCG transporter; Sus, reversible sucrose synthase; SUT, sucrose uptake transporter; SWEET, sugar transporter; VTC, vacuolar transporter chaperone; ER, endoplasmic reticulum; PD, plasmodesmata; PP pathway, pentose phosphate pathway; Poly‐P, polyphosphate; SP, short peptide; TCA cycle, tricarboxylic acid cycle; β‐MAG, β‐monoacylglycerol; InsP, inositol polyphosphate; Pi, inorganic phosphate; HPK, histidine protein kinase; RR, response regulator; P1BS, PHR1‐binding sequence; WRI, WRINKLED1‐like.

In the PFB continuum, metabolic networks are sustained by top‐down C flows and bottom‐up nutrient flows, with AM fungi acting as the central coordinator (Figure 7A). Plants typically allocate about 6% of their net photosynthates to AM fungi to facilitate this symbiosis [344]. Because AM fungi cannot cleave sucrose and synthesize fatty acids *de novo*, they rely on host‐specific metabolic processing in roots [345]. In the host cytoplasm, plant invertases and reversible sucrose synthases facilitate fungal sugar acquisition by processing sucrose into monosaccharides like glucose and fructose [346]. These are then exported via SWEET family transporters (e.g., SWEET1b) localized in the PAM into the PAS, where they are captured by high‐affinity fungal transporters (e.g., RiMST2) [347, 348]. Simultaneously, plants synthesize fatty acids (C16:0) in the plastids using mycorrhizal‐specific enzymes (e.g., DIS and FatM), which are then converted to β‐monoacylglycerols (β‐MAGs) by the specific enzyme RAM2 [253, 312]. These lipids are transferred to the PAS through PAM‐localized half‐size ABCG transporters, STR, and STR2, before being absorbed by the fungi to facilitate symbiotic development [349].

In the intraradical hyphae (IRH), these acquired C sources undergo multiple transformation to support the fungal lifecycle (Figure 7A). Lipids are elongated and desaturated into triacylglycerols (TAGs) and transported to the ERH as lipid droplets [350]. Plant‐derived monosaccharides enter glycolysis and the tricarboxylic acid (TCA) cycle or are converted into glycogen, the primary form for long‐distance sugar transport [351]. This internal management allows the fungi to buffer intracellular hexose levels and store energy in spores [352]. During asymbiotic stages, such as spore germination, AM fungi remobilize these reserves through the glyoxylate cycle and gluconeogenesis to synthesize structural polymers like chitin [353]. This metabolic flexibility ensures the fungal network maintains its structural integrity and metabolic activity even when reaching into nutrient‐poor soil patches far from the host.

AM fungi function as a rapid hub for translocating photosynthates to the hyphosphere [354]. Hyphal exudates, mainly comprising sugars, amino acids, carboxylates, and nucleic acids, act as a high energy “fuel” for the soil microbiota [296, 355]. While most soil bacteria remain dormant, these C‐rich exudates activate specific taxa such as Solibacterales and Myxococcales [58, 356]. Notably, components like fructose and carboxylates serve as signaling molecules that induce functional gene expression in bacteria. For example, fructose induces the expression of *R. aquatilis* phosphatase genes and carboxylate triggers the *Pseudomonas fluorescens nosZ* gene, accelerating *R. aquatilis*‐mediated organic P mineralization and reducing *P. fluorescens*‐mediated N_2_O emission [299, 357]. By facilitating this exchange, AM fungi do not just feed bacteria; they recruit functional partners that release inaccessible minerals, effectively integrating the three‐kingdom metabolic network and maintaining ecosystem stability [358].

#### Mineral flows in the plant‐AM fungus‐bacterium continuum

The hyphosphere and PAS serve as the primary sites for nutrient acquisition and exchange within the PFB continuum [296]. In nutrient‐limited soils, hyphospheric bacteria facilitate AM fungal nutrient uptake by secreting phosphatases and carbohydrate‐degrading enzymes (Figure 7A). These enzymes hydrolyze complex organic compounds into inorganic forms, such as phosphate (PO_4_^3−^), ammonium (NH_4_^+^), and nitrate (NO_3_^−^), which are then available for fungal absorption [299, 359]. This microbial activity is associated with the upregulated expression of mineral transporter genes in both ERH and IRH, increasing the efficiency of nutrient flux toward the host plant [343].

Phosphorus acquisition and transfer constitute a rigorously controlled molecular pathway (Figure 7A). The ERH absorb PO_4_^3−^ from the soil through high‐affinity PHOSPHATE TRANSPORTER 1 (PHT1) family transporters, including GintPT and GigmPT, localized on their plasma membranes [360, 361]. Once inside the fungal cytosol, the vacuolar transporter chaperon (VTC) complex (VTC1/2/4) polymerizes PO_4_^3−^ into poly‐phosphate (Poly‐P), which is sequestered into tubular vacuoles [362]. The long‐distance translocation of Poly‐P toward the host root is thought to be driven by water flow mediated by plant transpiration [363]. Upon reaching the IRH and fine arbuscular branches, fungal exopolyphosphatases (e.g., PPX1 and PPN1) hydrolyze the Poly‐P back into free PO_4_^3−^ [364]. The release of this PO_4_^3−^ into the PAS is mediated by specialized fungal exporters (e.g., RiPT7) [365]. An alternative proposed route suggests Poly‐P might be directly unloaded into the PAS and subsequently hydrolyzed by apoplastic acid phosphatases [362]. Finally, the free PO_4_^3−^ in the PAS is absorbed into plant cortical cells through mycorrhizal plant‐specific PHT1 family transporters localized on the PAM (e.g., MtPT4 and OsPT11) [366, 367]. This process strictly relies on plant H^+^‐ATPases (e.g., MtHA1 and OsHA1) to maintain the essential proton gradient across the PAM [368, 369].

Nitrogen transfer occurs through three distinct molecular routes. In the ammonium pathway, ERH transporters (e.g., GintAMT1) absorb soil NH_4_^+^ [278], which is assimilated into arginine via the GS‐GOGAT pathway and transported to the IRH alongside Poly‐P [370]. In the arbuscules, arginine is hydrolyzed to release NH_4_^+^, and then exported to the PAS and imported into root cells by plant transporters (e.g., ZmAMT3;1) [371]. In the nitrate pathway, NO_3_^−^ is acquired by potential fungal transporters (e.g., GiNT) [372], and then is either transformed into NH_4_^+^ or translocated directly to the PAS for uptake by plant transporters (e.g., OsNPF4.5) [373]. Concurrently, ERH transporters (e.g., RiPTR2) acquire organic N (e.g., dipeptides and oligopeptides) from soil, moving these peptides to the IRH for eventual import into host cells via putative PAM‐localized transporters [373, 374]. By integrating these specialized transport systems, the AM fungus‐bacterium partnership functions as an efficient biological nutrient‐recovery system (Figure 7A). This tripartite synergy establishes a comprehensive network that mobilizes chemically bound minerals from the soil matrix and directs them into the plant's metabolic streams, ensuring productivity in resource‐limited environments.

#### The regulation of carbon–phosphorus exchange at the peri‐arbuscular interface

Phosphate starvation response (PHR) transcription factors are central regulators of both P starvation‐induced gene expression and AM symbiosis [375, 376, 377] (Figure 7B). Under low P conditions, PHR proteins (e.g., OsPHR2 in rice) bind to P1BS (GNATATNC) cis‐elements in the promoters of symbiosis‐related genes, promoting AM fungal colonization and nutrient exchange [376]. Conversely, under high P conditions, P‐sensing SPX‐domain proteins bind to PHR transcription factors, preventing their interaction with P1BS elements and suppressing colonization [378, 379, 380]. However, the regulatory wiring of the SPX‐PHR module exhibits species‐specific divergence [381]. In *O. sativa*, SPX overexpression reduces colonization without impairing arbuscule development [376, 382]. In contrast, in *M. truncatula*, MtSPX1 and MtSPX3 positively influence early AM fungal colonization, yet their overexpression induces premature arbuscule degeneration, highlighting distinct transcriptional profiles between plant lineages [383].

These mechanistic differences are partially explained by the varied architecture of symbiosis‐induced gene promoters, which frequently contain MYC motifs (NTTCTTGTTN) and AW boxes (CG(N)7CNANG) adjacent to P1BS elements [384, 385]. While the P1BS element is conserved, the MYC element is critical for the expression of mycorrhiza‐induced P transporters in dicots (*e.g*., *MtPT4* and *LjPT4*) [386, 387], whereas it is absent in the *O. sativa* transporter *OsPT11*. Current models propose that PHR transcription factors enhance chromatin accessibility at these promoter regions [388], potentiating the activation of symbiotic genes by WRINKLED1‐like (WRI) transcription factors, which directly bind the MYC and AW elements (Figure 7B).

Consequently, WRI transcription factors function as master regulatory hubs governing the bidirectional exchange of carbon and phosphate at the symbiotic interface [389]. Evolutionarily conserved from liverworts to angiosperms [390], WRIs coordinate this exchange by coregulating lipid biosynthesis and P uptake. For example, MtWRI5a directly activates the promoters of both STR (mediating β‐MAGs export) and MtPT4 (mediating Pi import) [384]. While WRI expression is governed by the common symbiotic signaling pathway, its capacity to integrate host‐derived C signals with arbuscule development and bidirectional nutrient transport remains a primary focus for future molecular investigations.

#### Phosphate homeostasis inside the hyphae of arbuscular mycorrhizal fungi regulates carbon‐phosphorus exchange

AM fungi actively orchestrate Pi transport during symbiosis based on environmental availability and host plant demand. Tracking with fluorescent quantum‐dot nanoparticles demonstrates that AM fungi operate within a biological market framework: during environmental P surges, fungi sequester surplus P rather than immediately trading it, holding the resource until plant demand increases its biological value [391, 392]. Conversely, during P depletion, AM fungi remobilize internal reserves, preferentially transferring P to host roots exhibiting the highest nutrient demand [393].

This dynamic allocation is governed by the AM fungal PHO pathway (Figure 7B). Based on evidence from eukaryotes (*e.g*., yeasts, fungi, and plants), central to this mechanism are SPX domain‐containing proteins, which are hypothesized to sense cytosolic Pi fluctuations via interactions with inositol polyphosphates [365, 394, 395]. The PHO pathway is transcriptionally regulated by the transcription activator Pho4 and its co‐factor Pho2 [361]. Under P starvation, *R. irregularis* significantly upregulates *RiPho4*, which coordinates arbuscule development and downstream Pi transport components [276].

Bidirectional C‐P transfer supports the Luxury Resource exchange hypothesis, where nutrient exchange is strictly modulated by the internal resource status of both organisms [376, 396]. At the molecular level, this dynamic is mediated by the Pho4‐CACGTG/T *cis*‐regulatory module. Under P limitation, Pho4 translocates to the nucleus to bind CACGTG/T element (Figure 7B). Genomic analyses identify these motifs in numerous *R. irregularis* genes responsible for Pi (*RiPHO1*), monosaccharide (*RiMST2*), lipid (*RiABCGs*) transport, and stress‐response mediators like *RiMsn2* and *RiHog1* [277, 283, 347, 397]. Consequently, AM fungal Pho4 is proposed to function as a central regulatory hub governing internal C‐P exchange at the symbiotic interface. This regulatory architecture mechanically mirrors PHR system of the host plant, establishing an integrated, dual‐regulatory network that coordinates symbiotic nutrient exchange with environmental P fluctuations.

#### Crosstalk between arbuscular mycorrhizal fungi and hyphospheric bacteria

AM fungi actively secrete C‐rich hyphal exudates into the surrounding soil. Rather than a passive leakage of plant‐derived C, this exudation functions as a targeted mechanism to shape the hyphospheric microbiota [298]. By suppressing certain bacterial taxa while stimulating beneficial ones, AM fungi assemble a specialized core microbiome that compensates for their inherently limited saprotrophic capabilities [58].

A prominent feature of this microbial recruitment is the functional integration of phosphate‐solubilizing bacteria [297, 398]. The exuded fungal metabolites act as environmental signals that engage two‐component regulatory systems in specific taxa, such as *R. aquatilis*. This signaling cascade upregulates the transcription of genes encoding phosphatases and glucose dehydrogenases, thereby driving the solubilization of inorganic P and the mineralization of organic P [343]. Crucially, the outcome of this metabolic exchange is governed by local nutrient stoichiometry. Under severely deficient P conditions, bacteria compete with AM fungi for available resources, suppressing hyphal proliferation. Conversely, at moderately low phosphate concentrations, the dynamic shifts reciprocity, enhancing fungal nutrient acquisition and hyphae development [358].

Beyond P metabolism, this core microbiome expands the N‐acquisition capacity of the fungal network. Specific hyphospheric inhabitants, including *Paenibacillus* and *Streptomyces* species, secrete enzymes that degrade recalcitrant organic polymers like cellulose and chitin, effectively mobilizing soil organic N [359, 399]. Furthermore, the presence of *nifH‐*harboring bacteria introduces biological N fixation to the microhabitat. While the functional roles of these microbial assemblies are well established, the molecular signals that mediate this cross‐kingdom communication remain largely unresolved.

#### Crosstalk between endobacteria and arbuscular mycorrhizal fungi

Microscopic and molecular analyses have identified two primary lineages of vertically transmitted endobacteria residing in the cells of AM fungi [400]. The first group, *Burkholderia*‐related endobacteria (BRE, e.g., *Candidatus* Glomeribacter gigasporarum, *Ca*Gg), consists of rod‐shaped cells localized in fungal vacuoles and is largely restricted to the Gigasporales [401]. The second group, *Mycoplasma*‐related endobacteria (MRE, e.g., *Candidatus* Moeniiplasma glomeromycotorum, *Ca*Mg), features a coccoid morphology, resides directly within the fungal cytoplasm, and exhibits a broader phylogenetic distribution [402]. Both lineages display substantial genome reduction and diminished metabolic autonomy compared to their free‐living counterparts, indicating a highly specialized intracellular existence [403, 404].

Functional investigations utilizing cured, endobacteria‐free AM fungi lines have elucidated the strict metabolic dependencies defining these associations. Endobacteria rely entirely on the fungal host for C and mineral acquisition, including P and Zn. In return, the presence of BRE profoundly alters fungal physiology [353, 405]. Transcriptomic data demonstrate that BRE modulates approximately 25% of gene expression in the fungal host, significantly shifting fungal lipid profiles as a consequence of a decreased lipid flow from the plant to the fungus in the absence of the BRE [353]. Furthermore, these intracellular residents enhance the ecological fitness of the AM fungi by promoting hyphal elongation, increasing ATP synthesis, and upregulating pathways for ROS detoxification [406].

High‐resolution mass spectrometry has mapped the C trajectory across the plant‐AM fungus‐endobacterium continuum, revealing differential resource allocation by the host fungus. Tracing studies indicate that AM fungi allocate a greater proportion of plant‐derived C to MRE than to BRE, leading to hypotheses that MRE may function as opportunistic, weak parasites [407]. Because MRE‐cured AM fungal lines have not yet been generated, the precise ecological nature of the MRE‐AM fungus interaction remains indeterminate.

### Molecular cell biology and engineering of nodule and mycorrhizal symbioses

Throughout the course of land plant evolution, the establishment of mutualistic symbioses with soil microbes is a central adaptive strategy for coping with environmental nutrient limitation. Among diverse plant‐microbe interactions, arbuscular mycorrhizal symbiosis (AMS) and root nodule symbiosis (RNS) represent two of the most prominent mutualistic associations, and the underlying molecular mechanisms have been extensively investigated [248, 249]. AMS, which is formed between plant roots and fungi of the subphylum Glomeromycotina [249], is an ancient symbiosis that originated about 460 million years ago and has been retained across most land plant lineages [250, 251, 279]. AM fungi expand the absorptive surface of plant roots, thereby enhancing the acquisition of water and mineral nutrients, particularly phosphate and nitrate. In return, host plants provide fatty acids and sugars as carbon sources to the fungal partner [253, 312]. In contrast, RNS evolved more recently and is phylogenetically restricted, occurring primarily within the nitrogen‐fixing clade (NFC), which comprises four orders: Fabales, Cucurbitales, Fagales, and Rosales. NFC plants establish symbiotic associations with nitrogen‐fixing rhizobia through the formation of specialized root organs known as nodules [155, 254]. Within nodules, host plants supply malate and other organic acids as carbon sources to symbiosome‐enclosed rhizobia [218, 408], while the rhizobial nitrogenase complex consumes ATP to reduce atmospheric nitrogen into ammonia [408, 409]. In addition, nitrogen‐fixing bacteria of the genus *Frankia* can establish symbiotic relationships with actinorhizal plants [410].

#### Developmental stages of AMS and RNS

**FIGURE 8:**
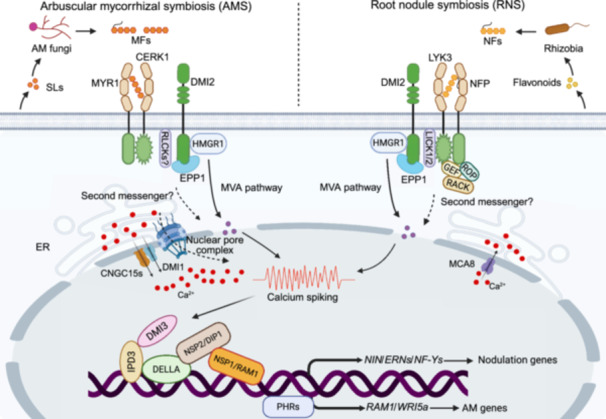
Common and distinct mechanisms underlying arbuscular mycorrhizal and root nodule symbioses. In both AMS and RNS, symbiotic signal perception follows similar molecular mechanisms. Mycorrhizal factors (MFs) are perceived by the CERK1‐MYR1 complex, while Nod factors (NFs) are perceived by the LYK3‐NFP complex. In RNS, LICK1/2 promote symbiotic signaling by activating MtLYK3. Unidentified RLCKs may function similarly in AMS. Following signal perception, AMS and RNS share a conserved signaling module. The co‐receptor DMI2/SYMRK interacts with downstream components to mediate the production of secondary messengers. HMGR associates with DMI2/SYMRK to activate the mevalonic acid (MVA) pathway, while EPP1 is phosphorylated by SYMRK to transduce cytoplasmic signals. MVA and other putative second messengers trigger nuclear calcium spiking mediated by DMI1, CNGC15 channels, and the Ca^2+^‐ATPase MCA8. Calcium spiking is decoded by CCaMK/DMI3, which phosphorylates IPD3. DELLA proteins link IPD3 to NSP2/DIP1, which further interact with NSP1/RAM1 to form distinct transcriptional complexes, ultimately activating gene expression programs specific to AMS or RNS. AMS, arbuscular mycorrhizal symbiosis; CCaMK, Ca^2+^/calmodulin‐dependent protein kinase; CERK1, CHITIN ELICITOR RECEPTOR KINASE1; CNGC15, Cyclic Nucleotide‐Gated Channel 15; DIP1, DELLA‐interacting protein 1; DMI1/2/3, DOES NOT MAKE INFECTIONS 1/2/3; EPP1, Early Phosphorylated Protein 1; HMGR1, 3‐hydroxy‐3‐methylglutaryl‐CoA reductase 1; IPD3, INTERACTING PROTEIN OF DMI3; LICK1/2, LYK3‐interacting cytoplasmic kinases 1/2; LYK3, LysM domain receptor‐like kinase 3; MCA8, Medicago truncatula calcium ATPase 8; MYR1, Myc FACTOR RECEPTOR1; NFP, Nod Factor Perception; NSP1/2, NODULATION SIGNALING PATHWAY 1/2; RAM1, REQUIRED FOR ARBUSCULAR MYCORRHIZATION 1; RNS, root nodule symbiosis; SYMRK, SYMBIOSIS RECEPTOR‐LIKE KINASE.

In the 19th century, German scientist Albert Bernhard Frank formally proposed the concept of “mycorrhiza” and defined it as a mutually beneficial symbiotic relationship [411]. Subsequently, Hermann Hellriegel and Hermann Wilfarth demonstrated that root nodules of leguminous plants establish symbiosis with soil microorganisms, allowing legumes to fix atmospheric nitrogen. Later, Martinus Willem Beijerinck successfully isolated rhizobia [412], marking the beginning of modern research on AMS and RNS. The successful establishment of AMS and RNS involves highly coordinated and complex biological processes (Figure 8).

Under phosphate starvation, the biosynthesis and exudation of strigolactones (SLs) are upregulated in plant roots. This process involves proteins such as the carotenoid isomerase DWARF27 (D27), CAROTENOID CLEAVAGE DIOXYGENASE7 (CCD7), and CCD8, the cytochrome P450 homolog MORE AXILLARY GROWTH1 (MAX1), and the ATP‐binding cassette transporter PLEIOTROPIC DRUG RESISTANCE1 (PDR1) [301, 413]. Secreted SLs function as rhizosphere signals that promote fungal spore germination and hyphal branching, thereby enabling AM fungi to establish physical contact with plant roots [300]. In turn, AM fungi secrete mycorrhizal factors, including lipochitooligosaccharides (Myc‐LCOs) and short‐chain chitin oligomers (COs, mainly CO4/CO5), which are perceived by host plants to initiate symbiotic signaling [55]. Following mutual recognition, fungal hyphae form an appressorium on the root epidermis and penetrate host tissues through a prepenetration apparatus (PPA) [310]. The fungus then grows toward the inner cortex, where it differentiates into highly branched arbuscules within cortical cells. Arbuscule formation requires extensive cellular remodeling, including membrane proliferation and organelle reorganization [414], thereby establishing a transient nutrient exchange interfaces before regulated arbuscule degeneration completes the symbiotic cycle.

In parallel, RNS is established through a coordinated sequence of infection and organogenesis, which is tightly regulated by host plants. Under nitrogen limitation, legume roots exude flavonoids to NodD proteins which are LysR‐5 type transcriptional regulators (LTTRs); NodD activates rhizobial nodulation genes, leading to the production of Nod factors (Nod‐LCOs) [415, 416, 417]. A recent study demonstrated that two pockets in NodD are essential for its activation by flavonoids, with key residues involved in specific recognition [167]. The length of the fatty acyl side chain and the specific modification of the GlcNAc backbone strongly influence host specificity [416, 417]. Recognition of Nod factors (NFs) induces root hair deformation, including swelling, branching and curling, which entrap rhizobia at the root surface. Localized cell wall remodeling and plasma membrane invagination then generate infection pockets in root hair cells, enabling bacterial accommodation [418]. This process culminates in the initiation of infection threads (ITs) [419]. ITs elongate under the guidance of the plant cell nucleus, allowing bacteria to move inward through the epidermis and cortical tissues [420, 421]. Concurrently, inner cortical and pericycle cells re‐enter the cell cycle to form a nodule primordium [422]. During these processes, the cytoskeleton, polarized vesicle trafficking, and localized cell wall remodeling are extensively reorganized and are essential for successful infection and nodule development [420, 422, 423, 424]. As the primordium develops into a functional nodule, rhizobia are released into host cells and enclosed within symbiosomes, where they differentiate into nitrogen‐fixing bacteroids [425]. The establishment of a microaerobic environment within nodules is essential for sustaining biological nitrogen fixation [424, 426].

#### Symbiotic signaling perception and recognition

In both AMS and RNS, symbiotic signaling perception and recognition follow broadly similar molecular principles. Myc factors (COs) and NFs are perceived by plasma membrane‐localized LysM‐type receptor‐like kinase complexes. In rice, a heteromeric receptor complex composed of Myc FACTOR RECEPTOR1 (OsMYR1) and CHITIN ELICITOR RECEPTOR KINASE1 (OsCERK1) mediates the perception of mycorrhizal signals (COs) [308, 427]. Similarly, in *Medicago truncatula*, mycorrhizal signal perception is mediated by a receptor complex formed by LysM RECEPTOR‐LIKE KINASE 4 (MtLYR4) and MtCERK1, also known as MtLYK9 [428]. In the liverwort *Marchantia paleacea*, the LysM‐containing receptor‐like kinases MpaLYR and MpaCERK1 are responsible for the perception of short‐chain chitin oligomers, such as CO4 [429]. In legumes, NFs are perceived by a receptor complex composed of the LysM receptor‐like kinase Nod Factor Perception (MtNFP) and the LysM domain receptor‐like kinase 3 (MtLYK3) in *M. truncatula*, or by Nod factor Receptor 5 (LjNFR5) and LjNFR1 complex in *Lotus japonicus* [430, 431]. Notably, upon NFs stimulation, symbiotic receptors accumulate within plasma membrane nanodomains, where they are stabilized by scaffold proteins such as flotillins and remorins, thereby enhancing signal amplification and specificity [432, 433, 434]. A similar nanodomain‐based regulatory mechanism may also operate during AMS [435].

#### CSSP‐mediated signal transduction

Despite marked differences in the morphology of their microbial partners and symbiotic structures, both AMS and RNS require tightly coordinated interactions between the host and symbiont, including signal exchange, infection, *de novo* membrane formation, and root developmental reprogramming. Central to these processes is a conserved host signaling framework, the CSSP, which connects microbial signal perception with intracellular signal transduction and transcriptional regulatory reprogramming [436, 437].

Following ligand perception by LysM receptor‐like kinases, the leucine‐rich repeat‐containing receptor‐like kinase SYMBIOSIS RECEPTOR‐LIKE KINASE (SYMRK), known as OsSYMRK in rice and LjSYMRK in *L. japonicus*, and DOES NOT MAKE INFECTIONS 2 (MtDMI2) in *M. truncatula*, is recruited and activated as a co‐receptor at the symbiotic plasma membrane [428, 438, 439, 440, 441]. SYMRK/DMI2 functions as a central signaling hub that relays signals from the plasma membrane to intracellular signaling components. However, the molecular intermediates that connect SYMRK/DMI2 activation to the generation of nuclear calcium spiking remain incompletely understood. One proposed component is 3‐hydroxy‐3‐methylglutaryl‐CoA reductase 1 (HMGR1), a DMI2‐interacting protein that catalyzes the conversion of HMG‐CoA to mevalonic acid (MVA) [442]. Exogenous application of MVA is sufficient to induce nuclear calcium spiking, suggesting that metabolites from the mevalonate pathway may function as secondary messengers in symbiotic signaling [443]. Early Phosphorylated Protein 1 (EPP1) exhibits increased phosphorylation levels following 1 h of NF treatment and is required for the activation of early rhizobial infection programs [444, 445]. EPP1 can be phosphorylated by DMI2 and promotes the production of secondary messengers during symbiotic signalling [446, 447]. However, the precise molecular mechanism underlying this regulatory process remains largely unclear. In addition, receptor‐like cytoplasmic kinases (RLCKs) act downstream of receptor‐like kinases (RLKs) by forming RLK‐RLCK complexes and transmitting ligand‐induced signals [448]. These kinases may represent important downstream components of symbiotic signal transduction. For example, LYK3‐interacting cytoplasmic kinases (LICKs) act as key regulators that attenuate host immunity during symbiosis while promoting symbiotic signaling [208].

At the nuclear periphery, several nucleoporins, including NUP85, NUP133, and NENA, are essential for the establishment of symbiotic calcium spiking [449, 450, 451]. Calcium‐permeable cyclic nucleotide‐gated channels, including CNGC15a/b/c, which are localized at the nuclear envelope function as major Ca^2+^ channels during this process [452]. The nuclear membrane protein DOES NOT MAKE INFECTIONS 1 (DMI1), together with its orthologs LjCASTOR and LjPOLLUX, is also indispensable for calcium spiking; these proteins may either contribute to ion conductance or act as pacemakers that regulate oscillatory frequency through interaction with CNGC15 channels [228, 453, 454], although their precise mechanistic roles remain to be fully elucidated.

Symbiotic calcium spiking is decoded by the Ca^2+^/calmodulin‐dependent protein kinase CCaMK, represented by LjCCaMK in *L. japonicus* and DOES NOT MAKE INFECTIONS 3 (MtDMI3) in *M. truncatula* [455]. LjCCaMK/MtDMI3 interacts with and phosphorylates LjCYCLOPS/INTERACTING PROTEIN OF DMI3 (MtIPD3), which functions as a transcriptional activator to convert calcium signals into transcriptional outputs [456, 457, 458]. Phosphorylated IPD3 further interacts with DELLA proteins to assemble distinct transcriptional complexes that activate AMS or RNS specific gene expression programs [459]. Through this conserved signaling module, extracellular microbial signals are ultimately translated into symbiosis‐specific developmental responses.

#### Transcriptional regulation in AMS and RNS

RAM1 is a GRAS family transcription factor that is induced upon AM fungal inoculation and plays a central role in AMS [460]. In AMS, phosphorylated IPD3 interacts with DELLA proteins and directly binds to the *LjRAM1* promoter to activate *LjRAM1* expression [461]. Additionally, the GRAS transcription factor DELLA‐interacting protein 1 (OsDIP1) interacts with both DELLA and RAM1, and is required for efficient mycorrhizal colonization [462]. RAM1 further directly targets the promoter of *RAM2*, which encodes a glycerol‐3‐phosphate acyl transferase [460]. Overexpression of *OsRAM1* increases the expression of *OsRAM2* in *O. sativa* [463], while overexpression of *MtRAM1* markedly upregulates *EXOCYST70I (MtEXO70I)*, *STUNTED ARBUSCULE (MtSTR)*, *WRINKLED5b (MtWRI5b)/MtWRI5c*, and *MtPT4* [464]. These findings suggest that RAM1 may function as a pioneer transcription factor that activates a broad spectrum of AM‐associated genes.

MtWRI5a, a member of the APETALA2 (AP2) transcription factor family, is another key regulator of bidirectional nutrient exchange during AMS [385]. In *M. truncatula*, MtWRI5a directly regulates the expression of *MtSTR* and *MtPT4*. Its homolog in *L. japonicus*, CTTC MOTIF‐BINDING TRANSCRIPTION FACTOR1 (LjCBX1), activates *LjPT4* and *LjHA1* [389]. Moreover, WRI5a and RAM1 regulate each other at the transcriptional level, forming a positive feedback loop that reinforces AMS [385]. Under phosphate starvation, plants activate a series of adaptive responses at the transcriptional, metabolic, and developmental levels, largely controlled by PHR proteins [465]. PHR transcription factors serve as key regulators of AMS [376]. As a central component of this transcriptional regulatory network, OsPHRs directly activate key mycorrhiza‐related genes, including transcriptional regulators such as *OsRAM1* and *OsWRI5a*, as well as nutrient exchange‐involved genes such as *OsPT11* and *OsAMT3;1*, by binding to the P1BS element in their promoters.

In RNS, DELLA proteins also function as linker proteins that assemble with IPD3 and NODULATION SIGNALING PATHWAY1/2 (NSP1/NSP2) to form a higher‐order transcriptional complex [459, 466]. This complex acts upstream in the symbiotic transcription network and directly activates the expression of *NODULE INCEPTION (NIN)* [467]. NIN belongs to the NIN like protein (NLP) subfamily of plant specific RWP‐RK transcription factors family, and functions as a core regulator throughout multiple stages of RNS [468]. Indeed, NIN represents a relevant innovation for the symbiotic nitrogen fixation [469]. During ITs formation, NIN directly regulates the expression of several key infection‐associated genes, including *Exopolysaccharide Receptor 3* (*EPR3*), *Rhizobial Infection Receptor‐like Kinase1* (*RINRK1*), *nodulation pectate lyase* (*NPL*), and *Rhizobium directed polar growth* (*RPG*) [470, 471, 472, 473].

The transcriptional regulation of *NIN* itself is also critical for nodulation. A *cis*‐regulatory element in the *NIN* promoter, known as PACE (Pol‐box Associated Cis‐regulatory Element), is essential for the formation of cortical cells carrying infection threads [474]. NIN is also required for nodule organogenesis. It directly binds to the promoter regions of *NF‐YA1*, which encodes a member of the Nuclear Factor‐Y transcription factor family. Mutations of *NF‐YA1* result in delayed infection, abnormal infection, and the formation of smaller white nodules [475, 476]. Consistently, overexpression of *LjNIN* or *LjNF‐YA1* induces cell division in the root cortex [475, 477]. The FR motif, a 15‐amino‐acid motif located immediately downstream of the RWP‐RK domains is required for NIN function. Deletion of the FR motif abolishes the ability of NIN to bind *NF‐YA/NF‐YB* and other NIN‐specific elements. Nodules formed by FR‐deletion mutants lack nitrogen‐fixing capacity [477]. NIN also regulates nodule organogenesis by recruiting developmental program associated with lateral roots formation and phytohormone signaling [478, 479, 480, 481]. In addition, proteolytic cleavage of NIN generates a C‐terminal fragment that activates genes involved in symbiosome development and nitrogen fixation [150]. Besides NIN, ERF REQUIRED FORNODULATION (ERN1) is a key transcriptional regulator of rhizobial infection [482]. Moreover, key components of the stem cell regulatory program, including SHORT ROOT (SHR) and SCARECROW (SCR), also contribute to nodule organogenesis; the SHR‐SCR network initiates legume‐specific cortical cell division for *de novo* nodule organogenesis after perceiving rhizobial signals [483].

Taken together, these transcriptional regulators integrate symbiotic signaling, infection, nutrient exchange, and developmental reprogramming to ensure the successful establishment of AMS and RNS.

#### Engineering plant‐microbe symbiosis under agricultural nutrient regimes

In natural ecosystems, plants acquire limiting nutrients by establishing symbioses with soil microbiomes such as AMS and RNS. In modern agricultural systems, however, crop production often relies on intensive chemical fertilizer inputs to achieve high yields [484]. Excessive fertilizer application can suppress beneficial symbiotic interactions, including both AMS and RNS. Because both AMS and RNS require substantial carbon and energy investment from the host plant, these associations are strongly suppressed under nutrient‐sufficient conditions. Therefore, if AMS and RNS can be more efficiently integrated into agricultural production systems with moderate fertilizer input, it offers an important strategy to improve nutrient use efficiency and achieve long‐term agricultural sustainability. Under high nitrate conditions, the NIN‐like protein 1 (NLP1) accumulates in the nucleus and interacts with NIN, thereby suppressing the expression of NIN target gene *CRE1* [485]. Under high phosphate conditions, SPX domain‐containing proteins function as phosphate sensors and suppress PHR activity, leading to reduced activation of mycorrhizal symbiotic programs [376]. In addition to these local nutrient‐responsive mechanisms, plants have evolved systemic regulatory pathways, including autoregulation of nodulation (AON) and autoregulation of mycorrhization (AOM), to control the extent of symbiotic colonization [486]. Therefore, under moderate fertilizer‐input systems, optimizing AMS and RNS may improve nutrient acquisition efficiency and support sustainable agricultural development. The root‐derived CLE (CLAVATA3/EMBRYO SURROUNDING REGION) peptides are the key regulatory components of the AON and AOM pathways [487, 488]. A recent study reported that two *ric1a/ric2a* mutants exhibited increased grain yield and protein content in multi‐year and multi‐site field trials [489], highlighting the potential of engineering systemic symbiotic regulation to improve crop performance under agricultural nutrient regimes.

#### Engineering nitrogen‐fixing symbiosis into non‐legume cereals

During natural evolution, only species within NFC acquired the capacity to establish nitrogen‐fixing symbioses with rhizobia. Transferring this symbiotic competence to cereal crops could substantially improve nitrogen use efficiency and reduce dependence on synthetic nitrogen fertilizers. AMS is widespread among land plants, including most cereal crops. Moreover, RNS is thought to have evolved through the recruitment of components from the ancient AMS signaling pathway. The presence of conserved CSSP components in cereals suggests that these plants already possess part of the molecular machinery required for symbiotic signaling and may therefore potentially be engineered to acquire rhizobial nitrogen‐fixing capability [490]. In wild‐type rice, treatment with low concentrations of NFs does not induce calcium oscillations. However, co‐expression of the chimeric receptors MtLYK3‐OsCERK1 and MtNFP‐OsMYR1, generated by replacing the extracellular domains of OsCERK1 and OsMYR1 with those of MtLYK3 and MtNFP, enables rice to produce calcium oscillations in response to low concentrations of NFs [308]. This finding demonstrates that cereal plants can be engineered to perceive rhizobial signals and activate downstream symbiotic calcium signaling, providing an important step toward the reconstruction of nitrogen‐fixing symbiosis in cereals. Recently, a study introduced nine key genes of the nodulation signaling pathway into *O. sativa* to generate Nodulation Signaling Pathway Overexpression (NSPO) rice. The NSPO rice exhibited enhanced formation of 2,4‐d‐induced nodule‐like structures [491].

Receptor engineering has also provided insights into the molecular determinants of symbiotic signal specificity. LjCERK6 functions as a chitin receptor for CO8 and is essential for plant immune signaling. Structure‐guided domain swapping revealed that regions II and IV within the LysM1 domain are major determinants of ligand specificity. Replacement of these regions in LjCERK6 with the corresponding motifs from LjNFR1 reprogrammed its ligand‐binding specificity, enabling the engineered receptor to recognize and bind NFs [479, 492]. In addition, two amino acid residues located in the Symbiosis Determinant 1 (SD1) region of the LjNFR1 transmembrane‐juxtamembrane region are crucial for the initiation of nodulation signaling. Substitution of these two LjNFR1‐specific residues into LjCERK6 and its barley homolog HvRLK4 is sufficient to confer symbiotic signaling capacity on these chitin receptor kinases, enabling them to initiate root nodule symbiosis in *L. japonicus* [493].

Beyond signal perception, transcriptional reprogramming is another central requirement for engineering nitrogen‐fixing symbiosis in cereals. Key symbiotic transcriptional regulators coordinate diverse developmental and physiological programs, including stem cell fate determination, lateral root development, stress responses, and nitrate signaling [155, 483, 486]. Through these regulators, multiple cellular processes are integrated to support successful symbiotic establishment. However, in cereal crops, the promoters of many corresponding pathway genes lack the cis‐regulatory elements required for symbiosis‐specific transcription factor binding. Therefore, large‐scale promoter engineering to introduce appropriate cis‐regulatory elements remains a major challenge. With the rapid development of genome editing and synthetic biology technologies, this bottleneck may eventually be overcome, opening new possibilities for engineering nitrogen‐fixing symbiosis in non‐legume crops.

#### Interactions between the rhizosphere microbiome and soil protozoa

Among the diverse soil biota, protozoa are the most abundant and dominant bacterial consumers in the rhizosphere, playing irreplaceable roles in shaping microbial community structure and function [494]. The rhizosphere microbiome and protists engage in multifaceted, bidirectional communication, forming an intricate regulatory network that is critical for maintaining plant health, nutrient cycling efficiency, and soil ecosystem stability [495].

Protozoa influence the structure and function of rhizosphere microbial communities through various mechanisms. First, protozoa can markedly alter bacterial community composition. Selective predation by protozoa tends to reduce the abundance of copiotrophic bacteria such as Proteobacteria, while promoting the proliferation of oligotrophic groups like Acidobacteria. This shift can optimize carbon and nitrogen fluxes in the rhizosphere [60, 496]. In addition to compositional changes, this selective pressure also modulates ecosystem functioning by influencing the expression of functional genes within the microbial community [60, 497, 498]. Second, protozoa play a central role in rhizosphere nutrient cycling. By grazing on bacteria, they convert nitrogen immobilized in microbial biomass into ammonium, thereby facilitating nutrient remineralization [499]. In other words, protozoa release ammonium from the nitrogen that was previously incorporated into bacterial cells. The presence of protozoa has been shown to significantly enhance bacterial nitrogen mineralization efficiency [500], which in turn accelerates the decomposition of soil organic matter and subsequent nutrient release [501]. Furthermore, protozoa regulate microbial functions in the rhizosphere through chemical signaling. A notable example is *Acanthamoeba castellanii*, which triggers the synthesis of 2,4‐diacetylphloroglucinol (2,4‐DAPG) in biocontrol *Pseudomonads*, thereby enhancing their resistance to soil‐borne pathogens [502]. This top‐down regulatory pathway highlights the potential application of protozoa in rhizosphere biological control.

Similarly, rhizosphere microorganisms exert top‐down regulatory pressure on protozoa. They modulate the spatial distribution and foraging behavior of protozoa through reciprocal chemical communication [503]. For example, rhizosphere bacteria such as *Pseudomonas* species, release acyl‐homoserine lactone (AHL) signals via quorum‐sensing pathways, which can influence the predation intensity and prey selectivity of protozoa [504]. Shifts in microbial community structure also govern protozoa growth and reproduction [505]. As an illustration, bacteriophages‐bacteria interactions considerably affect the size and diversity of bacterial communities, thereby modulating resource availability for protozoa grazers [506, 507]. Through such bidirectional regulation, the rhizosphere microbial food web maintains ecological equilibrium and functional efficiency.

The reciprocal influences between protozoa and rhizosphere microorganisms collectively contribute to plant growth and health, forming a complex regulatory system [495]. In the context of soil‐borne diseases suppression, predatory protozoa directly consume pathogens such as *Ralstonia* spp. and *Fusarium* spp., which may contribute to reduced disease incidence [505]. Concurrently, protozoa promote the proliferation of beneficial biocontrol strains, including *Bacillus* spp. and *Pseudomonas* spp., and stimulate the upregulation of antibiotic biosynthesis genes (including *Nonribosomal peptide synthetase* and *Polyketide synthase*), thereby enhancing the antagonistic activity of these beneficial bacteria against pathogens [506]. Protozoa such as soil amoebae also encourage biocontrol agents to form thicker biofilms, reinforcing the defensive barrier at the root surface [506]. With respect to plant nutrition, protists enhance nutrient acquisition by releasing nutrients immobilized in microbial biomass through their grazing activities [508, 509]. For example, protozoa grazing facilitates the remineralization of nitrogen and phosphorus, improving their availability to plants, particularly in high P‐fixing soils [510, 511]. Furthermore, soil protists contribute to crop productivity by enhancing plant growth‐promoting (PGP) traits such as siderophore production, ammonia synthesis, and biofilm formation in response to grazing pressure [512]. At the same time, protist grazing reshapes the composition and function of rhizosphere bacterial community [513], indirectly improving plant tolerance to environmental stresses [514].

In summary, protozoa‐microbe interactions in the rhizosphere constitute a sophisticated bidirectional regulatory network driven by selective predation, chemical signaling, and trophic cascades. This regulation not only reshapes the composition and functional potential of microbial communities, but also translates into measurable benefits for plant growth and health through enhancing nutrient mineralization, reinforcing biological control, and priming systemic resistance. Consequently, protozoa‐microbial interplay represents a critical nexus for maintaining rhizosphere ecosystem functioning, extending beyond the conventional plant‐microbe binary paradigm that has historically dominated rhizosphere research.

Future studies should integrate single‐cell sequencing [515] and live‐imaging technologies [516] to resolve the in situ spatiotemporal dynamics of protozoa‐microbial interactions and their long‐distance modulation of plant immunity. Furthermore, the design of synthetic food webs incorporating protozoa holds great promise for the development of next‐generation precision agriculture strategies based on the targeted manipulation of soil trophic networks.

### The aerial root mucilagesphere: A reframed microhabitat for functional microbiome assembly and stability

**FIGURE 9:**
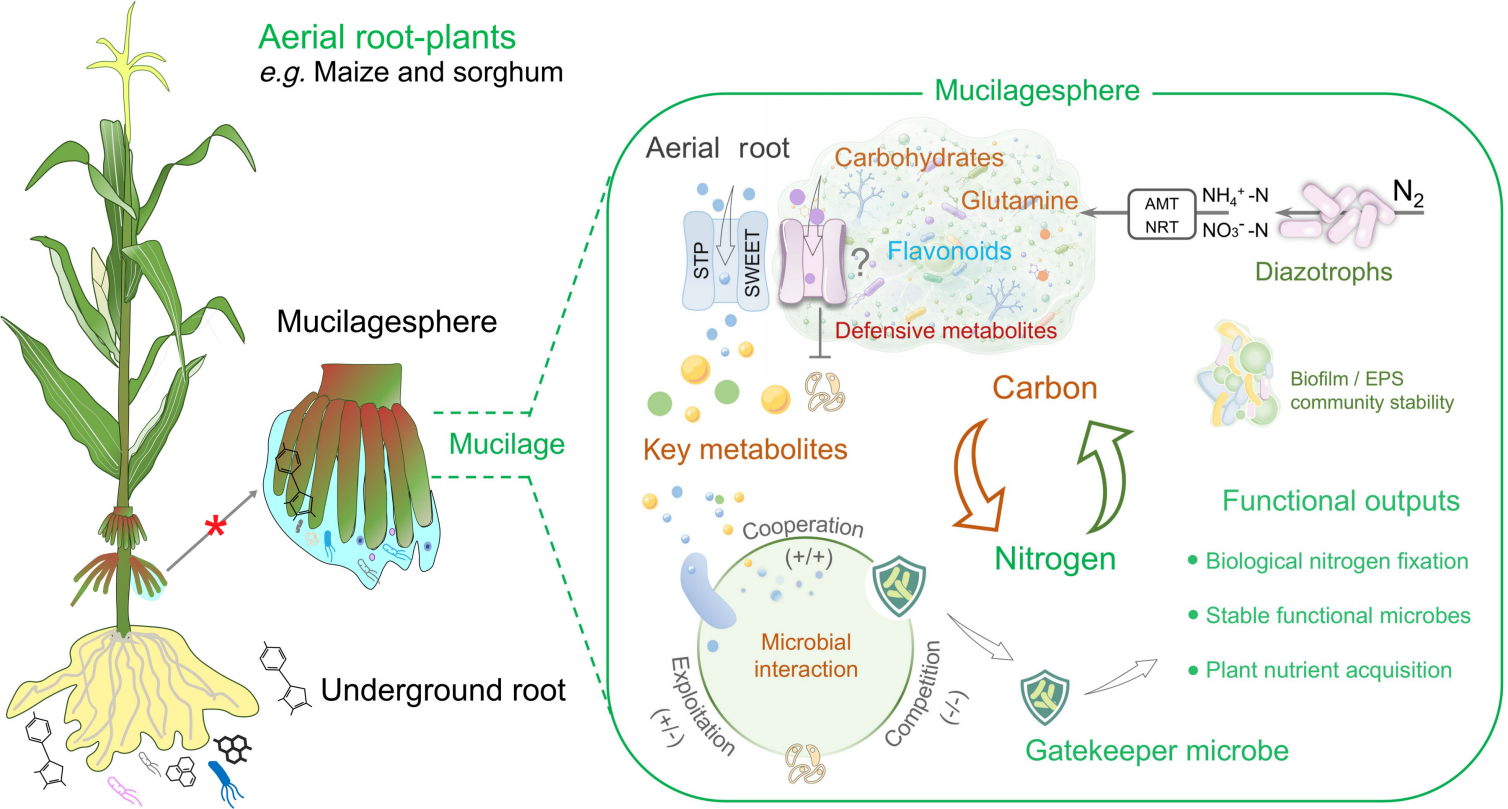
Conceptual model of plant aerial root mucilage‐microbiome interactions. Plants allocate carbon through mucilage secretion and carbohydrate transport (e.g., via SWEET and STP transporters), creating a nutrient‐rich niche that recruits and sustains diverse microbial communities, including diazotrophs. Root‐derived compounds such as flavonoids and defense‐related metabolites further modulate microbial composition and activity. Within this microenvironment, microbes engage in complex interactions, including cooperation, competition, cross‐feeding, and biofilm (EPS) formation, which collectively influence community assembly and function. Diazotrophic bacteria fix atmospheric N_2_ and contribute to nitrogen availability (e.g., NH_4_^+^), which can be assimilated by the host through nitrogen transport systems (*e.g*., AMT and NRT transporters). These processes are embedded within a dynamic and potentially host‐regulated framework of microbiome plasticity, where targeted carbon investment and chemical signaling shape microbial function and enhance nitrogen acquisition under specific environmental conditions.

Beyond the well‐studied soil interfaces, recent studies have shown that some aboveground plant structures also generate chemically distinct microhabitats capable of supporting specialized microbial communities. A representative example is the mucilage secreted by aerial roots. Here, we use the term “Aerial Root Mucilagesphere (ARM)” (Figure 9), A previously underexplored niche that extends traditional views of nutrient acquisition beyond the rhizosphere to aboveground organs [109, 517]. This concept does not simply extend the rhizosphere upward; rather, it highlights a separate microhabitat in which nutrient acquisition, microbial filtering, and community stability are shaped under aboveground environmental conditions.

Aerial root mucilage differs from typical belowground root exudates in both physical context and chemical composition. It is rich in soluble carbohydrates, amino acid derivatives, and secondary metabolites, with easily metabolizable sugars often representing a prominent fraction [109, 518]. This carbon‐rich environment can support rapid microbial growth, but the resulting community is not highly diverse. Instead, available evidence suggests that aerial root mucilage tends to select for a narrower set of microorganisms with specific functional capacities [517, 518, 519]. Such a pattern implies that host‐derived mucilage acts not only as a nutrient source but also as an ecological filter [109].

One defining feature of the mucilagesphere is the consistent enrichment of nitrogen‐fixing microorganisms. Bacterial taxa including *Burkholderia*, *Herbaspirillum*, and *Klebsiella* have been frequently reported in aerial root mucilage and are thought to contribute to biological nitrogen fixation (BNF) [109, 518, 519, 520]. Isotope tracing experiments further demonstrated that the nitrogen fixed in this microenvironment can be taken up and utilized by plant tissues, accounting for as much as 54% − 82% of the total BNF [518, 519]. In certain plant systems, this pathway represents a significant source of nitrogen input, indicating that the mucilagesphere functions as an aerial extension of nutrient acquisition strategies [520]. Nevertheless, the extent of aerial root‐mucilage contribution is likely to vary among plant species, developmental stages, and different environmental conditions, and should therefore be interpreted as a context‐dependent strategy rather than a common trait.

The maintenance of a functional mucilage‐associated microbiome appears to depend on reciprocal interactions between the host and microbial residents. This “carbon‐nitrogen exchange” is a bidirectional interaction in which plants secrete carbon‐rich mucilage, while the symbiotic microorganisms in return may provide nitrogen fixation products or other growth‑promoting substances (Figure 9). Compounds such as flavonoids, organic acids, and phenolic substances may serve dual roles. [109, 518]. On one hand, they act as chemical cues or nutrient sources that attract beneficial microbes [109, 521]. Consistent with this interpretation, transcriptomic evidence has revealed enhanced expression of genes related to carbon allocation (e.g., via SWEET and STP transporters) and nitrogen acquisition (e.g., AMT and NRT transporters) in mucilage‐aerial root tissues [109]. On the other hand, they function as defensive agents that limit the establishment of harmful organisms. Although the precise regulatory pathways of aerial root‐mucilage remain to be fully elucidated, it is evident that plant metabolic outputs are central to microbiome structuring.

In addition to metabolic cooperation, the stability of the mucilagesphere is closely linked to mechanisms that regulate microbial balance. Despite its nutrient‐rich nature, this habitat does not favor uncontrolled microbial proliferation. Instead, it maintains a relatively stable and functionally coherent community [518, 519]. This is mainly thanks to certain microbial regulators, often called “gatekeepers” [518]. These microbes hold back opportunistic or harmful species while still letting the helpful ones stick around. Take some fungi; for example: they have broad antimicrobial effects, but they don't wipe out the nitrogen‐fixing bacteria living alongside them, so the system keeps working as it should.

Compared with the conventional rhizosphere, the aerial root mucilagesphere has several distinctive features. It forms aboveground, is exposed to fluctuating humidity and oxygen availability, and may change with plant developmental stage. These properties make it a more transient and environmentally sensitive habitat than many soil‐associated interfaces. At the same time, its relatively simplified community structure may provide a useful model for dissecting host control over microbiome assembly without the full complexity of soil.

From an agricultural applied perspective, the aerial root mucilagesphere offers a potentially useful system for improving biological nitrogen fixation and reducing dependence on synthetic fertilizers. However, practical application will require a clearer understanding of three issues: which host traits promote stable mucilage formation, which microbial members are essential for nitrogen fixation and community regulation, and how different environmental variation affects the persistence of this system. Addressing these questions may support the development of targeted bioinoculants or engineered microbial consortia, but such applications should be approached cautiously until the mechanisms of host‐mucilage‐microbe specificity are better resolved. All in all, the mucilagesphere on aerial roots is a pretty unique spot where plants and microbes meet above ground. It matters for how roots take up nutrients and keep their microbial communities stable. What's interesting isn't just that nitrogen‐fixing microbes live there‐it's also how the mucilage made by the plant pulls together carbon flow, which microbes stick around, and how the whole community stays in balance. Looking at it this way, we get a broader picture of the microbial habitats connected to plants. It also gives us a good starting point to explore how plants manage helpful microbes, not just in soil but in other settings too.

### Phyllosphere microbiome

#### Leaf microbiome

##### The leaf as a unique microbial habitat

The leaf microbiome comprises complex microbial communities that colonize both the surface and the internal tissues of plant leaves, representing the largest microbial habitat in the aboveground parts of plants [522]. The total leaf surface area of terrestrial plants worldwide is estimated at 6.4 × 10^8^ km^2^ [523], offering a vast ecological niche for microorganisms. Although the leaf surface is generally considered an oligotrophic environment, where access to carbon, nitrogen, and micronutrients may be limited and subject to competition among resident microbes [524]. However, due to localized nutrient‐rich microsites such as veins and trichome bases, leaves still harbor relatively diverse microbial communities.

The physical structure of leaves significantly influences the colonization patterns of microorganisms [525]. Features such as the arrangement of epidermal cells, cuticle characteristics, distribution of stomata and salt glands, density of trichomes, and venation architecture collectively create a distinct “micro‐topography” for microbial colonization [516]. Research has shown that microorganisms tend to accumulate in nutrient‐rich microsites, including leaf veins, the bases of trichomes, grooves of epidermal cells, and areas surrounding stomata [524]. The waxy components and hydrophobic properties of the cuticle determine the wettability and nutrient permeability of leaves, thereby directly influencing microbial attachment and growth [19]. In fact, cuticular hydrophobicity is considered a major barrier to microbial colonization, as it impedes the formation of a continuous water film on the leaf surface, thereby limiting the movement and germination (such as many fungal spores) of microorganisms that require free water [526]. However, microorganisms are not merely passive recipients of these surface constraints. Many have evolved strategies to actively modify their immediate environment. For example, certain bacteria (such as *Pseudomonas syringae*) produce biosurfactants that alter cuticle permeability, thereby gaining more effective access to nutrients and enhancing their colonization on leaf surfaces [527]. Therefore, the leaf surface represents a dynamic and challenging habitat [528], and microbial success depends largely on the ability to adapt to its fluctuating conditions.

##### Composition and diversity of leaf microbial communities

The leaf microbiome is composed of a wide array of microorganisms, including bacteria, fungi, archaea, yeasts, and protists. Among these, bacteria are typically the most dominant and abundant colonizers of leaf surfaces [529]. These bacterial communities are generally dominated by four major phyla, including Proteobacteria, Firmicutes, Actinobacteria, and Bacteroidetes [530]. Such community structures have been observed across a diverse range of plant species, including bioenergy crops (switchgrass) [79], annual dicots (e.g., *Arabidopsis*, clover, lettuce, and spinach) [531, 532], and perennials (e.g., various tree species) [533]. Moreover, many of these microorganisms engage in significant interactions with their host plants. Prominent epiphytic bacterial genera include *Pseudomonas*, *Methylobacterium*, and *Sphingomonas* [16, 534, 535]. These bacteria are capable of utilizing volatile organic compounds released by plants, such as methanol as carbon sources, thereby playing a crucial ecological role in the leaf microenvironment [536].

In addition, the fungal community associated with leaves also exhibits considerable diversity, encompassing various classes within Ascomycota and Basidiomycetes, as well as a substantial number of yeasts [537]. The core fungal microbiota commonly includes filamentous genera such as *Acremonium, Alternaria, Aspergillus, Cladosporium, Mucor*, and *Penicillium* [537] along with the yeast *Aureobasidium pullulans* [535]. While many of these taxa are considered widespread inhabitants of the phyllosphere, certain groups display host specificity [538].

Rice, as one of the world's most important food crops, offers a valuable model system for studying plant‐microbe interactions in the phyllosphere. The rice leaf microbiome harbors a rich and diverse microbial community, shaped in part by the plant's unique growth environment, which alternates between aquatic and terrestrial conditions. Bacterial communities associated with rice leaves are predominantly composed of Proteobacteria, Actinomycetes, and Bacteroidetes. Notably, genera such as *Methylobacterium, Sphingomonas, Pantoea*, and *Khuskia* [539] have been found to consistently colonize rice leaves across different genotypes and environmental conditions. Among these, *Methylobacterium* is particularly prevalent and is known for its ability to fix nitrogen and produce PGP substances such as cytokinin, thereby conferring beneficial effects on host growth and development [536]. However, these effects are often strain‐specific, highlighting the importance of selecting compatible host‐microbe combinations for agricultural applications [19].

##### Assembly and determinants of the leaf microbiome

The leaf surface serves as a crucial interface where plants directly interact with the external environment [523]. The assembly of microbial communities on this surface is a dynamic process shaped by complex interactions between the host plant and microorganisms, as well as by environmental filtering [524]. This process begins with the dispersal of microorganisms across different media. Airborne transmission, insect‐mediated transfer, soil splash, and human activities all contribute to the initial species pool available for leaf colonization [535, 540, 541]. Subsequently, the unique micro‐environment of the phyllosphere selectively filters potential colonizers through physical and chemical barriers, thereby establishing an initial microbial community. On this basis, microbe‐microbe interactions and active recruitment by the host plant further drive community assembly and help maintain microbiome homeostasis [523].

**FIGURE 10:**
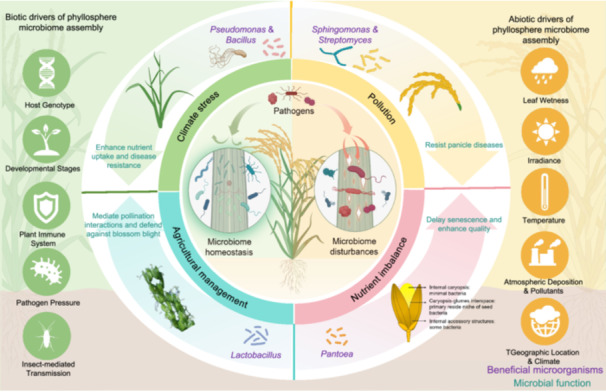
The role of the phyllosphere microbiome in plant health and stability. This figure illustrates key aspects of phyllosphere microbiome assembly, regulation, functional differentiation, and disturbance. The assembly and structure of phyllosphere microbiome are jointly regulated by multiple biotic and abiotic drivers. The green section on the left side of the figure highlights the major biotic drivers, including host genotype, developmental stage, the plant immune system, pathogen pressure, and insect‐mediated transmission. The yellow section on the right side shows the major abiotic drivers, including leaf wetness, irradiance, temperature, atmospheric deposition and pollutants, and geographic location and climate. Pronounced niche‐specific differentiation in both community composition and functional attributes is observed across plant organs (including leaves, flowers, fruits, and panicles) in crops such as rice. Beneficial microbial communities on leaves contribute to nutrient acquisition and stress tolerance; those associated with flowers mediate pollination interactions and suppress floral diseases; fruit‐associated microbiota help delay senescence and improve quality; and panicle‐associated microbes play critical roles in resistance to panicle diseases, including rice blast. Microbiome homeostasis is maintained through the combined effects of community diversity, interspecific interactions, and host immune signaling, forming a structurally stable symbiotic system. This stability suppresses pathogen colonization via mechanisms such as resource competition, antagonism, and induction of plant immunity, thereby supporting normal plant growth and function. However, this homeostasis is dynamically regulated by external drivers. Key stressors, including climate stress, agricultural management practices, nutrient imbalance, and environmental pollution, can disrupt microbiome structure by altering resource availability, microenvironmental conditions, and microbial interaction networks, thereby driving a transition from homeostasis to microbiome disturbance. Once this balance is disrupted, community structure becomes disordered, facilitating pathogen enrichment and infection, ultimately leading to the onset and spread of plant diseases.

The assembly of the leaf microbiome is shaped by the interplay of biotic and abiotic factors, whose complex interactions ultimately determine community structure [542] (Figure 10). Among the biotic factors, the species characteristics of the host plant, such as leaf morphology [543], physiological metabolism [22], and immune competence [544] are considered among the most crucial determinants. Variations in leaf microhabitats across different plant species or varieties directly influence the diversity of the microbial community [545]. Furthermore, dynamic changes during plant growth and development modulate community succession by altering nutrient availability and leaf defense responses [546]. For example, microbial communities undergo ecological succession as leaves mature, transitioning from pioneer assemblages to more stable, climax communities [547].

Abiotic factors indirectly shape leaf microbiome assembly by modulating both microbial activity and the physiological status of the host plant [548]. Climatic variables, particularly temperature, humidity, and irradiance, are key determinants in this context [549]. Extremes such as heat stress and drought significantly reduce microbial diversity while concurrently enriching stress‐tolerant taxa [550] (Figure 10). Anthropogenic disturbances, such as fertilization regimes [551], pesticide applications [552], and genetic modification [88], can disrupt the natural processes community assembly by altering leaf microhabitats or directly compromising microbial survival. Long‐term pesticide use, for example, has been associated with declines in beneficial microorganisms on leaves, whereas well‐managed agricultural practices, including the application of biological agents, offer potential to optimize microbiome structure and function [553].

##### The ecological functions of the leaf microbiome

The leaf microbiome harbors several critical ecological functions that contribute to plant health and broader ecosystem processes. First, phyllosphere microorganisms play an active role in global biogeochemical cycling, particularly in the transformations and cycling of carbon and nitrogen. Functional traits such as biological nitrogen fixation, ammonia oxidation, and organic matter decomposition are widespread among leaf‐associated microbial communities and are crucial for plant nutrient acquisition [554]. Second, the leaf microbiome serves as the first line of defense against invasion by pathogenic microorganisms. In healthy plants, the resident microbiota can suppress pathogen colonization through mechanisms including competitive exclusion, secretion of antimicrobial compounds, and induction of systemic resistance in the host [555]. Moreover, certain PGP bacteria inhabiting the phyllosphere, analogous to their rhizosphere counterparts, can influence plant growth, development, and responses to environmental stimuli by synthesizing phytohormones such as auxin (IAA), cytokinin (CK), and gibberellin (GA) [555].

In recent years, the potential applications of the leaf microbiome in agricultural production and food safety have garnered considerable attention. Approaches to intentionally shape beneficial phyllosphere communities, such as modulating fertilization regimes, refining cultivation practices, and developing microbial inoculation [556], have emerged as important research directions in sustainable agriculture and plant protection.

### Microbiome of plant reproductive organs

From inflorescence to infructescence, plants go through their reproductive growth stage. During this process, the interaction between microorganisms and reproductive organs, such as flowers and fruits, play important roles in plant health. In particular, fruits contain seeds, the carrier of intergenerational transmission of plant genomes and microbiomes.

#### The community composition and influencing factors

Due to the variability in the morphology and physiological structure of plant reproductive organs (including flowers, fruits, and seeds), the composition of their microbial communities varies greatly among different plants. The microbial community of plant reproductive organs is influenced by location [557], soil type [558], plant taxonomy [558], and the host developmental stage [559, 560, 561]. Although there are some common taxa in the floral microbiota among different plants, different studies whose plant samples were collected from different areas revealed distinct floral core microbes of multiple plant species [562, 563]. This indicates that the floral microbiota is largely affected by location and plant species. Nevertheless, *Alternaria* and *Cladosporium* are consistently the floral common core of various plants in different studies [562, 563]. Moreover, the common bacteria of rice seeds among plenty of rice species and cultivars unveiled by different studies are similar, such as *Pantoea*, *Pseudomonas*, and *Sphingomonas* [557, 564]. These results indicate that different plants exhibit a certain degree of commonality in their selective filtering of microbes dwelling in their reproductive organs, maybe involved in convergent evolution.

#### The sources

There are three sources for the microbiota of plant reproductive organs: (i) the environment, mainly the air and rain; (ii) parent plants; and (iii) pollinators, especially insects such as honey bees, hoverflies, and so on. As early as 1999, Stockwell et al. found that the flowering period is the key time for bacteria to colonize on pear blossoms [565]. Chen et al. revealed rice grain bacteria predominantly originate from the external environment during panicle heading and flowering stage, through quantitative PCR and synthetic bacterial community [98]. Soil microorganisms can migrate to the plant phyllosphere during the very early developmental stage of new plants, from seed germination to the emergence of the first true leaf [566]. Whether they continue to migrate to the reproductive organs as the plant development will be the key evidence in determining if soil is a source of the microbial communities in plant reproductive organs. It is generally believed that endophytes of parent plants can enter plant reproductive organs through the xylem or nonvascular tissue of stems and leaves [92, 93]. The direct evidence was provided for bacteria entering seeds through parent plants until last year, by inoculating GUS‐labelled wheat endophytic isolate during the germination of parent plants and then detecting the colonization of the bacteria in the seedlings of the progeny plants [97]. However, the visualization of the process by which microorganisms migrate from the parent plant to its flowers or seeds is still lacking. As pollinators, insects such as bees can facilitate the horizontal transmission of microbes through nectar [567, 568]. But it is still unclear whether the microbes transmitted to nectar by pollinators can reach the stigma and subsequently enter the fruits and seeds.

#### The function and mechanism

There are diverse beneficial microbes in the microbiota of plant reproductive organs, which regulate host health through multiple functions and mechanisms. The research focus of microbiome functions of different plant reproductive organs is as follows: (i) the interaction among flower or nectar, pollinators and pathogens [569, 570, 571]; (ii) the correlation between diseases and the microbiota during fruit development and storage [572, 573, 574]; microbes, such as *Lactiplantibacillus pentosus* [575] and *Cryptococcus laurentii* [576], which help fruits extend their storage life and improve the quality of fruits; and (iii) the regulation of seed microbiota on plant disease resistance, including at the panicle stage of parent plants [577, 578] and at the seedling stage of offspring plants [69, 579].

The microbiome of plant reproductive organs regulates host health in direct or indirect ways. The direct way is that some key members in microbiome antagonize pathogens by acting on diverse molecular targets. For example, *Pantoea agglomerans* colonized in apple flowers competes with the pathogen *Erwinia amylovora* for arabinogalactan, thus inhibiting the expression of pathogenic virulence factors [570]. Through detoxifying plant chemical defenses, fruit microbe *Pantoea* enables safe consumption by frugivorous birds. The microbe transforms fruit chemical defense from a dispersal barrier into a microbe‐mediated seed dispersal mechanism, illustrating an intricate three‐way mutualism [580]. Indirectly, the microbiome improves plant disease resistance by modulating host physiological states. Keystone microbial taxa, *Lactobacillus* spp. and *Aspergillus* spp., induce rice panicles to secret branched‐chain amino acids which interfere the metabolic homeostasis of *Ustilaginoidea virens*, thereby enhancing host resistance to rice false‐smut disease [577]. *Pseudomonas* species in wheat spikes counteract the pathogen‐induced alkalinization by secreting organic acids, leading to the suppression on Fusarium head blight caused by *Fusarium graminearum* [90]. Through direct antagonism and indirect regulation of host physiology, the microbiome establishes the second defense layer of plant reproductive organs. Further exploring the interaction between the microbiome of plant reproductive organs and the plant immune system, as well as whether reproductive organ microbes can establish an acquired genetic microbiome defense through intergenerational transmission, will help us obtain a deeper understanding of the adaptive evolution of plant holobiont.

### Phyllosphere microbiome homeostasis and plant health

#### Phyllosphere microbiome homeostasis: Regulators and plant health

The phyllosphere is often subjected to dramatic variations in temperature, humidity, ultraviolet (UV) radiation, and nutrient accessibility, making it a more challenging habitat for microbial colonization and survival [581]. Maintaining phyllosphere microbial community homeostasis, a dynamic balance modulated by microbial interactions and host genetic regulation, is critical for plant growth, stress tolerance, and overall health; its disruption (dysbiosis) markedly increases physiological disorders and disease susceptibility. The homeostasis of microbial communities in this niche is a dynamic ecological and evolutionary process, shaped by a variety of signals originating from the microorganisms themselves, their host plants, and the environmental interfaces with which the phyllosphere interacts [582]. While the intrinsic characteristics of microbes, such as hydrophobic adhesive forces, have been thoroughly investigated and clarified [583], the factors associated with host plants and the potential impacts from the aerial interface remain insufficiently understood. Elucidating how these diverse factors regulate microbial community homeostasis in the phyllosphere is a fundamental and indispensable prerequisite for harnessing the potential of beneficial microbial taxa and their functional traits to maintain plant health.

#### Interplay between microbiota homeostasis and plant immunity

In contrast to the conventional view that plant immunity primarily acts to restrict pathogenic microbes, emerging evidence has revealed a reciprocal interplay between microbiota homeostasis and immunocompetence, in which a eubiotic microbiota is not merely a consequence of host defense but a prerequisite for proper immune function. Using *Arabidopsis thaliana* as a model system, the He group identified a core plant genetic network dedicated to maintaining microbial balance in the phyllosphere [88]. This regulatory circuitry integrates pattern‐triggered immunity (PTI), HopM1 interactor 7 (MIN7)‐dependent vesicle trafficking, and constitutively activated cell death 1 (CAD1)‐mediated signaling to modulate microbial community structure, limit the overgrowth of potentially harmful taxa, and preserve commensal‐dominated homeostasis. This work established that plants actively govern phyllosphere microbiota stability through dedicated genetic pathways, rather than passively tolerating microbial colonization.

More recently, in gnotobiotic plant systems and defined synthetic communities, dysbiotic microbiota was found to be capable of triggering aberrant and excessive transcriptional activation of immune pathways, leading to autoimmunity‐like phenotypes and impaired plant performance [584]. Reconstitution with a healthy, balanced microbial community restored normal immune regulation and suppressed inappropriate defense activation. These findings established a causal relationship: a eubiotic microbiota is required to gate proper immune responsiveness, ensuring that immune reactions remain calibrated and non‐detrimental to host fitness.

These seminal studies illustrate a deeply integrated regulatory loop between plant genetics, microbiota homeostasis, and immune function. Host genetic networks maintain microbial eubiosis in the phyllosphere, and in turn, a balanced microbiota permits appropriate, controlled immunity rather than uncontrolled inflammation or immunodeficiency. This bidirectional interaction redefines our understanding of plant‐microbe coadaptation and highlights microbiota homeostasis as a central determinant of plant health. How plants prevent dysbiosis and how commensals shape immunity are key to engineering beneficial microbiomes that enhance crop resilience and disease resistance.

#### Plant genetics and chemical crosstalk

Plants can maintain microbiome homeostasis through endogenous factors; in addition to the plant developmental stage, age, and species richness reported previously, the genetic factors and their underlying molecular mechanisms remain poorly understood [585]. Using a synthetic community to investigate host effects on the phyllosphere community composition and abundance, the alleles that resulted in the strongest perturbation of the microbiota relative to the wild type (WT) were *lacs2* and *pec1* in Arabidopsis, which suggests that the host genotype can shape the associated phyllosphere microbiota [20].

Accumulated evidence shows that the capacity to shape the microbiome through specific genes has been conserved in various plants, including barley, cucumber, maize, and rice. A recent study using Genome‐wide association studies (GWAS) on 110 rice accessions identified 235 SNP‐containing loci associated with specific microorganisms, with four bacterial orders (including Pseudomonadales, Burkholderiales, Xanthomonadales, and Enterobacterales) responsive to host genetics and linked genes enriched in the phenylpropanoid pathway [19]. *indica* and *japonica* rice differ in the OsPAL02 haplotype, which mediates 4‐hydroxycinnamic acid (4‐HCA) synthesis. 4‐HCA recruits *Pseudomonas*, inhibits *Xanthomonas oryzae*, and maintains phyllosphere microbiome homeostasis [19]. Therefore, OsPAL02 regulates microbiome homeostasis and plant health via 4‐HCA biosynthesis. These findings collectively indicate that microbiome genes are likely to be distributed and conserved in plant hosts, leading to their designation as the *M* genes.

In the context of disease resistance breeding, the *M* gene enables both the enrichment of disease‐suppressive microbiota and a direct disease‐suppressive effect. Current resistance breeding, known as the ‘*R* gene breeding strategy’, is increasingly challenged by rapidly evolving pathogens. In contrast to an alternative breeding strategy based on editing the *S* (susceptibility) gene in the host, harnessing the *M* gene emerges as a novel microbiome‐targeted strategy [22]. This strategy may facilitate the breeding of crop varieties that are more broad‐spectrum and durable in disease resistance. One prominent feature of *M* genes is that they are responsible for the regulation of chemical substance biosynthesis, suggesting the core role of the intricate chemical crosstalk mediated by various molecules between plants and their microbiota in regulating microbiome homeostasis [586]. In the future, a systematic understanding of the regulatory pathways that govern the homeostasis of microbial communities will open up new avenues for developing crop cultivars that are friendly to functional microbial taxa.

#### Underestimated environmental perturbation and Anthropocene

Recent evidence highlights the substantial influence of the environment on phyllosphere microbiota assembly [587]. Plants in close proximity often harbor highly conserved bacterial communities across leaves, flowers, or even different host species [588]. Even genetically identical plants display variable microbial structures under diverse environmental conditions. In model plants including Arabidopsis and rice, environmental factors such as geographic location, season, temperature, moisture, and CO_2_ significantly shape phyllosphere microbial composition [589]. Rainfall, wind, light, and local microbial dispersal also affect microbial colonization and community diversity, with neighboring plant identity, biomass, and age acting as key determinants.

In the context of global change, emerging atmospheric perturbations, such as elevated ozone, microplastics, and PM2.5, have drawn growing attention, yet their impacts remain underestimated [590]. Elevated ozone has been correlated with altered phyllosphere microbiota, compromised host disease resistance, and reduced grain quality, although causal relationships remain unclear [591]. Microcosm experiments will be essential to identify microbial responders to such perturbations and characterize resilient beneficial taxa, which can improve microbiome‐assisted plant modulation.

The Anthropocene also exerts profound, bidirectional effects on phyllosphere microbiome homeostasis. Urbanization gradients have been linked to shifts in tree leaf microbial communities. While agrochemicals, fertilizers, and nanotechnologies may boost short‐term crop performance, their long‐term unintended effects on commensal microbes remain poorly understood. Agricultural nanomaterials, such as nanosilver, pose potential nanotoxicity risks to non‑target organisms and native microbiota by disrupting rhizosphere microbial homeostasis and reducing crop productivity [592].

Given that agricultural ecosystems are open and complex, microbiome impacts must be integrated into risk assessments of anthropogenic inputs. Human activities targeting aboveground plant tissues and aerial surfaces continuously shape phyllosphere communities and threaten ecosystem integrity, warranting greater research focus.

#### Innovative detection methods based on the low abundance characteristics of phyllosphere microbiomes

The rapid advancement of sequencing technologies has greatly promoted the flourishing development of plant microbiome research. However, general sequencing processes are not suitable for plant phyllosphere microbiomes due to the low abundance of phyllosphere communities and the contamination from plant organellar DNA. Plant phyllosphere, including plant leaves, stems, flowers, fruits, and seeds, is a low‐abundance microbial niche. Phyllosphere microbiota, especially endophytes, is much lower than the rhizosphere in microbial quantity, community richness and diversity. Quantitative analyses of the bacterial community found that endophytic bacteria in *Arabidopsis* leaves are 10^5^ CFU (colony‐forming unit) per gram [fresh weight], accounting for only 1/1000 of the total leaf bacteria [88], and the endophytic bacteria of rice leaves (10^6^ per gram [fresh weight]) is 1/1000 of that in rice roots [593]. 16S rRNA gene profiling studies revealed that the bacterial species richness of rice stems and seeds is about 1/10 or even less than that in rice roots, and the community diversity of stems and seeds is less than half of that of the roots [557]. Based on 50 wild flowering plants representing 22 families, researchers found that the species richness and diversity of both bacterial and fungal communities in flowers are significantly lower than those in leaves [558]. Collectively, plant phyllosphere microbiota, especially the endophytes, possess low bacterial content.

Plant chloroplast 16S rRNA gene and mitochondrial 18S rRNA gene share high sequence similarity in conserved regions with bacterial 16S rRNA genes, leading to plant DNA contamination in 16S rDNA amplicon libraries [594]. The low abundance characteristic of plant phyllosphere microbiota results in extremely severe contamination of plant organellar DNA in amplicon libraries, making it difficult to obtain high‐quality libraries through data filtering. To address this issue, various methods to avoid organellar DNA amplification have been developed, including PNA (Peptide nucleic acid)/LNA (Locked nucleic acid) clamps, CRISPR/Cas strategy, and bacteria‐specific primer pairs. Lundberg et al. designed PNA clamps which specifically block the DNA of plant plastid (pPNA) and mitochondria (mPNA), thus the amplicon library of the second‐round PCR is free of plant organellar DNA [595]. LNA clamps can also block plant organellar DNA [596]. PNA clamps have been widely used in the microbial sequencing of plant leaves [597], flowers [558], and seeds [564]. CRISPR/Cas strategy utilizes nuclease Cas9 and designed gRNAs that target plant organellar DNA to eliminate plant DNA‐derived amplicons of the first‐round PCR, thereby reducing the plant DNA contamination rate of the second‐round PCR products to less than 15% [598]. 799F is the first primer to avoid the co‐amplification of plant chloroplast 16S rRNA gene [599]. 799F and 1193R [27] is a commonly used primer pair for the sequencing of plant phyllosphere microbiota. By agarose gel electrophoresis, the mitochondrial 18S rDNA amplicons of this primer pair can be divided from bacterial 16S rDNA amplicons for most plants, like *Arabidopsis* [584], wheat [600], and so on. However, the 799F/1193R amplicon of rice mitochondrial 18S rDNA is around 300 bp shorter than in other plants, leading to a length only about 50 bp greater than that of bacterial 16S rDNA, which hampers resolution by agarose gel electrophoresis. Chen et al. designed two primer sets, 322F‐Drs/796R (V3–V4) and 799F/1107R (V5–V6), which can simultaneously avoid the co‐amplification of mitochondrial and chloroplast DNA. The primer set 322F‐A/796R was further used for fluorescent quantitative PCR to quantify the bacterial content of rice plants at the nucleic acid level. Moreover, these two primer sets are applicable for various plants [593]. Furthermore, the quantitative abundance of plant microbiota samples can be obtained by combining the qPCR absolute quantification results with the relative abundance of bacterial communities [98]. These innovative detection methods provide powerful tools for plant phyllosphere microbiome research.

### Endophytic and seed microbiome

Having surveyed the microbial communities inhabiting below‐ground interfaces (rhizosphere) and above‐ground surfaces (*e.g*., mucilagephere and phyllosphere), we now turn our focus on endophytic and seed microbes. This section provides a detailed examination of the endosphere, the internal plant tissues, and the seed microbiome, emphasizing their unique transmission modes, co‐evolution with the host.

### Plant endo‐microbiome

#### Definition and transmission of plant endo‐microbiome

**FIGURE 11:**
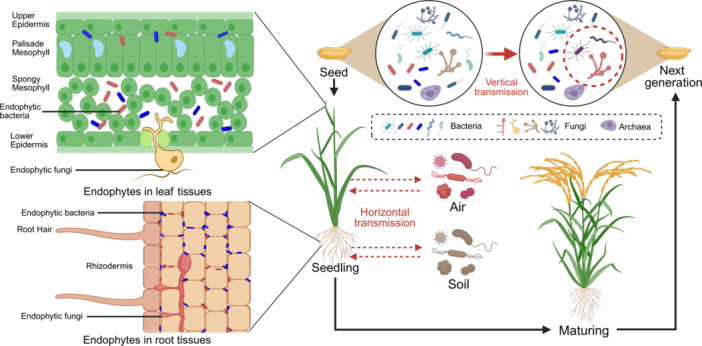
The shaping process of plant endo‐microbiome and seed microbiome. Plant endo‐microbiomes and the seed microbiomes together constitute a microbial link for intergenerational transmission of plant microbiomes. Certain seed microorganisms, including bacteria, fungi, and archaea, inherited vertically from the parent plant, migrate to the roots and aboveground parts of the next‐generation seedlings upon germination, becoming the core endophytes. Meanwhile, through horizontal transmission, environmental microorganisms from the soil, air, and other media can enter the plants and colonize as new endophytic members. At the mature stage, some successfully colonized endophytes can migrate back to the developing seeds, thereby supplementing or reshaping the seed microbiome and completing an intergenerational cycle of plant endophytes.

The term “endophyte” came to refer primarily to microorganisms, mainly bacteria and fungi, that inhabit internal plant tissues (e.g., roots, leaves, and seeds) during at least part of their life cycle without causing apparent disease symptoms to their host by the early 1990s [601]. The plant‐endophyte interactions may exhibit different modes of symbiotic association, ranging from beneficial (mutualism), neutral (commensal), to even pathogenic [602]. Beneficial endophytes facilitate the establishment of symbiotic association within plants and contribute to the development of plant endo‐microbiome, which in turn mutualistically endorses and protects plant from diverse environmental stresses [603]. Generally, endophytic microorganisms can be acquired by plants through both vertical transmission and horizontal transmission (Figure 11). Vertical transmission refers to the transfer of microorganisms from parent to offspring via seeds, wherein seed‐borne endophytes play critical roles in early plant development and certain taxa are stably transmitted across generations [604]. Horizontal transmission refers to microorganisms being re‐acquired from the surrounding environment, especially the soil, with approximately 50% of endogenous bacterial taxa exhibiting rhizospheric origins, and less than 10% fungal communities derived from the soil [605]. Although soil harbors a high diversity of microorganisms, its influence on the wood microbiome is limited, suggesting that the wood microbiome is shaped by multiple potential sources beyond soil [606]. This dual mode of acquisition significantly contributes to the diversity and composition of endophytic communities within plants [603].

#### Diversity and composition of plant endo‐microbiome

Plant endo‐microbiome exhibits exceptionally high diversity, richness, population count with community structures shaped collectively by host genotype and tissue compartments (e.g., leaves, stems, and roots) [603]. A study on endophyte richness patterns across plant tissue types indicates that variation in endophyte assemblages in above‐ground tissues depends on host growth habit, with stems being the richest tissue in woody plants, whereas roots are the richest in graminoids [607]. Among bacteria, Firmicutes, Proteobacteria, Actinobacteria, and Bacteroidetes are typically the dominant phyla, with common core genera such as *Bacillus*, *Burkholderia*, *Pseudomonas*, *Enterobacter*, and *Pantoea* [608]. While most endophytic fungi are represented by Ascomycota, with a few belonging to Basidiomycota, with genera such as *Epichloe* and *Balansia* (clavicipitaceous endophytes), and *Colletotrichum*, *Fusarium*, and *Trichoderma* (non‐clavicipitaceous endophytes) being widely distributed [609]. The diversity and relative abundance of endophyte species within the endo‐microbiome can further shift in response to environmental changes, such as drought and salt stress [603]. Additionally, endophyte‐endophyte interactions, including competition, mutualism, and antagonism, can also shape community shifts. For example, one endophyte species may produce compounds that inhibit competitors or enhance host fitness, thereby altering endophytic community composition [31].

Nowadays, with the development of advanced high‐throughput sequencing and next‐generation sequencing, our understanding of endophyte diversity has been greatly expanded. However, a large number of unculturable endophytic microorganisms still constitute the so‐called “microbial dark matter”, whose functions require further exploration [610].

#### Co‐evolutionary processes between plant and their endo‐microbiomes

The co‐evolutionary process between endophytes and plants shapes their interactions: endophytes are likely to have originated from pathogens, while an endophytic phase may also play a crucial role in the life cycle of pathogens, as evidenced by studies in fungal endophytes [609]. This co‐evolutionary dynamic may reflect patterns of ecological relatedness in host‐associated microbial communities, a concept known as “phylosymbiosis”, which supports a hypothesis‐driven framework for illustrating a new paradigm bridging host‐associated microbiomes [611]. Among the endo‐microbiome, the population stability of non‐pathogenic ones within plants is maintained through a dynamic balance in multiplication and death, thereby ensuring the long‐term coexistence of endophytes and their host [612]. The co‐evolution between plants and endophytes is evident across multiple levels of molecular interaction and ecological adaptation. Plants distinguish beneficial microorganisms from pathogens through pattern recognition receptors (PRRs), while beneficial microorganisms coordinate growth‐defense trade‐offs in plants through microbe‐associated molecular patterns (MAMPs) [613]. This “arms race” drives co‐evolution of both partners, exemplifying typical Van Valen's Red Queen hypothesis [614]. Notably, an economic trade‐off exists in plant‐endophyte interaction, where plants support endophytes with photosynthetic products in exchange for microbe‐mediated host fitness benefits, whereas this resource allocation strategy determines the stability of symbiotic relationships [615].

#### Functions of plant endo‐microbiomes

Broadly, beneficial plant endo‐microbiomes enhance host nutrient acquisition (*e.g*., nitrogen via fungal endophyte *Phomopsis liquidambaris* [616]; phosphorus via fungal endophyte *C. tofieldiae* [72] or bacterial endophyte *Flavobacterium* [617]), plant growth (e.g., via fungal endophyte *Tinctoporellus* [618]), and stress resistance (e.g., salt stress via bacterial endophyte *Pseudomonas* [619], drought stress via fungal endophyte *S. indica* [620]) through various mechanisms, including synthesizing phytohormones (e.g., indole‐3‐acetic acid [621]), fine‐tuning plant physiological processes (e.g., ferroptosis induction [622]; phenylpropanoid‐auxin axis [618]), and interacting with other plant‐associated microbiota [623] (e.g., DSE and soil microbiome [76]; fungal endophyte and rhizosphere microbiome [624]). Furthermore, plant endophytes act as the second layer of plant defense, exhibiting significant potential in regulating host disease resistance [625, 626]. For example, a recent study proposed a “Sentinels” strategy in which genetically engineered plant endophytes serve as sentinel strains initiating effector expression upon pathogen invasion, thereby precisely activating the plant immune system and conferring host disease resistance [627]. Meanwhile, beneficial endo‐microbiome plays a dominant role in synthesizing novel metabolites, such as alkaloids and phenols [621]. The functional traits of these plant endophytes thus position them as a promising candidate for sustainable agriculture.

### Seed microbiome

#### Definition and composition of seed microbiome

Seed microbiome is defined as the microbial communities that are located internally or externally and inhabit or interact with seeds [106, 628], including both endophytic and spermosphere microbiota [628, 629]. Seed microbiome can be categorized into core and transient communities [630]. Transient communities are mainly composed of opportunistic or environmental microbes that colonize only temporarily, while core communities persist stably across diverse plant genotypes and environmental conditions [630]. A large‐scale meta‐analysis of seeds from 50 species systematically identified approximately 30 core taxa where the predominant bacterial phyla include Proteobacteria, Actinobacteriota, Firmicutes, and Bacteroidetes, with *Pseudomonas*, *Xanthomonas*, *Bacillus*, *Pantoea*, and *Enterobacter* being the most widespread genera [103]. A recent study has revealed that *Pantoea* is widely present in wheat seeds worldwide through vertical transmission [97]. While the classes Dothideomycetes, Sordariomycetes, and Tremellomycetes are dominant in fungal seed microbiome [105].

The structure of the core seed microbial community varies across plant species, even among different subspecies of the same species (as found in rice [631]), which are attributed to the selective effects imposed by host phenotypic variation [105, 632].

#### Evolution and transmission of seed microbiome

Plants and seed‐associated microorganisms form a mutually beneficial holobiont and establish a tightly coordinated co‐evolutionary relationship [633]. This co‐evolution pattern is also encapsulated by the concept of phylosymbiosis, demonstrating congruence between host phylogenetic distance and microbiome composition [564]. For example, domestication profoundly reshapes the seed endophytic microbial communities in cereals [564, 634].

The coexistence of diversity and stability in the seed microbiome is largely determined by the origin of seed microorganisms and their assembly rules. Previous studies have demonstrated that the sources of seed microbiota are diverse, primarily including three pathways: vertical transmission (parent‐to‐seed), horizontal transmission (environment‐to‐seed) and pollinator‐mediated transmission [92, 97, 630] (Figure 11). Among these, parental vertical transmission, through reproductive tissues such as flowers, ovules, and fruits, is considered the foundation for the formation of seed core microbiomes [92, 103, 604]. Additionally, microbes in paternal pollen can also influence the composition of endophytic microbiomes in seeds [635, 636]. Through vertical transmission, certain bacteria and fungi are transferred across generations, forming relatively stable associations with their host [97, 637]. Therefore, seeds act as a bridge for the succession of the plant microbiome across generations, which is essential for crop domestication.

Compared to vertical transmission, environmental sources have relatively minor yet significant impacts by continuously introducing diverse microbial members into seeds [638], thereby reshaping the community structure during seed development, dispersal, and germination [639]. The spermosphere serves as a significant source of the endophytic seed microbiome [639]. It is noteworthy that, although most microbial genera detected in seeds are present in soil, the seed microbiome differs significantly compared to the soil microbiome [640], suggesting that host plant exerts filtering and selection effects on horizontally transmitted microorganisms [104].

From fertilization to germination, seeds experience a range of unique and fluctuating environmental conditions that drive complex microbial community dynamics [641]. For example, in *Amorphophallus muelleri*, the composition and function of seed endophytic bacterial communities are shaped by seed maturity status [642]. Upon germination, seed microbes colonize the inner tissues like radicles and plumules of next‐generation seedlings [643], thereby shaping the future rhizosphere, endosphere, and phyllosphere microbiota [96, 639, 644] (Figure 11). It is also demonstrated that the seed microbiome can even spread to the soil and enhance the nutritional conditions for host plants [645, 646]. These processes thus allow seed‐borne microorganisms to interact with soil‐derived microbial communities that are horizontally recruited by the host post‐germination. Such interactions include “niche competition” or “cooperative assembly”, thereby further shaping plant‐associated microbial communities.

#### Functions of seed microbiome

Although the microbial biomass in seeds is relatively low, the seed microbiome is closely associated with seed development, storage, germination, seedling establishment, and plant growth [105]. With the development of multi‐omics technologies, seed microbiomes have gained significant potential for agricultural applications [105, 630].

During germination process, seed endophytes are able to resume metabolic activities after seed hydration [647], and induce host phytohormone biosynthesis or directly synthesize phytohormones [105]. For example, *Bacillus subtilis* QM3 can promote wheat seed germination by inducing gibberellin biosynthesis of the host plant [648]. In addition, seed microorganisms can enhance seed resistance to pathogens by inducing host‐induced systemic resistance (ISR), competing for ecological niches, and producing antimicrobial substances [69, 639]. For example, a large‐scale screening of 260 seed lots from seven different crops (e.g., beetroot, onion, spinach, and pepper) demonstrated that certain seeds may contain beneficial microorganisms capable of suppressing seedling‐stage diseases [649]. In addition, seed microbiome can assist seeds in coping with abiotic stresses such as drought, chilling, salinity, and heat [105]. Collectively, these findings highlight that the seed microbiome functions as a multifaceted symbiotic consortium that supports plant growth, stress resilience, and pathogen defense from germination through early seedling establishment.

### Plant pathobiome

#### From single‐pathogen paradigms to community‐level pathobiome

While the preceding sections have focused on how individual microbes, whether beneficial or pathogenic, interact with the plant host, accumulating evidence indicates that plant diseases rarely result from infection by a single pathogen acting in isolation [650]. Instead, disease outcomes are frequently shaped by complex microbial communities in which co‐infecting pathogens, opportunistic microbes, and resident commensals collectively influence host susceptibility and symptom severity [2]. This ecological perspective has given rise to the concept of the plant pathobiome, defined as the assemblage of pathogenic and disease‐facilitating microorganisms that, together with the host and its environment, determine the trajectory of disease development [651]. When the balance of the resident microbiota is disrupted, a state termed dysbiosis, normally commensal or endophytic microbes may shift toward pathogenic behavior, further compounding disease severity [652]. The pathobiome framework represents a natural extension of the microbiome‐centered view discussed above, shifting the focus from single‐pathogen causality to community‐level disease dynamics. Understanding the pathobiome is critical for designing management strategies that target not only the primary pathogen but also its microbial allies, as discussed in the following sections. Disruption of the balanced plant microbial community can trigger disease outbreaks in plants.

**FIGURE 12:**
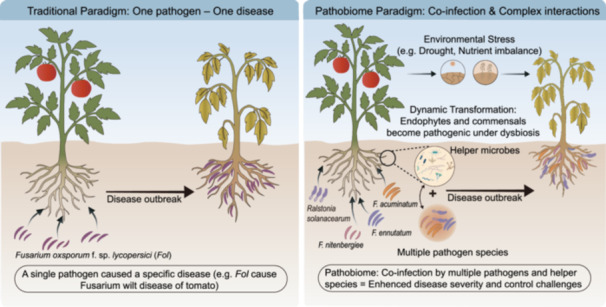
Conceptual shift from the traditional single‐pathogen paradigm to the plant pathobiome paradigm in understanding plant disease. Traditional paradigm: one pathogen‐one disease. In the classical framework, plant disease is attributed to infection by a single causal agent. For example, *Fusarium oxysporum* f. sp. *lycopersici* is considered the sole cause of Fusarium wilt disease in tomato. Disease development is explained through a linear relationship between one pathogen and one disease outcome. Pathobiome paradigm: co‐infection and complex microbial interactions. In this framework, disease is an emergent outcome shaped by the core pathogen, co‐infecting pathogens, disease‐facilitating microbes, and environmental conditions. The core pathogen can restructure the local microbiota and recruit helper microbes that promote its colonization and virulence expression. Meanwhile, environmental stresses may trigger dysbiosis, converting commensal or endophytic microbes into pathogenic agents. Consequently, disease severity is not determined by a single pathogen species but emerges as a community‐level property of cross‐kingdom microbial interactions. This perspective supports integrated management strategies that target both the core pathogen and its key microbial partners.

The traditional “one pathogen, one disease” paradigm is giving way to a more comprehensive view, represented by the concept of the “pathobiome”. For example, the Koch's postulates provided plant pathology with a powerful but deliberately simplified framework that one pathogen, one disease (Figure 12). For over a century, this linear causal logic guided the identification of disease agents and the design of control strategies, and its contributions to understanding virulence mechanisms and host recognition remain significant. Yet the assumption that a single microbial species acts as both the necessary and sufficient cause of disease is inconsistent with the complexity of what is actually observed in the field. Many plant diseases resist explanation by single‐agent models, and control measures directed at one pathogen species often fail to deliver lasting protection. The gap between laboratory‐defined pathogenicity and field‐level disease outcomes has become difficult to ignore.

Microbiome‐scale surveys of diseased plants have begun to close this gap. Multiple independent studies indicate that plant diseases frequently involve not just the primary pathogen but a broader consortium of microorganisms whose composition and activity shape virulence, transmission, and host response [37, 651]. The term “pathobiome” was introduced to reflect this reality (Figure 12), referring to the causative pathogen together with the associated microbial community that modulates disease outcome [37]. The plant pathobiome has been defined as a functionally distinct assemblage arising when an invading core pathogen directionally restructures the rhizosphere or phyllosphere microbiota, recruiting certain members into a cooperative network that collectively disrupts plant homeostasis [653]. Under this framework, disease is not merely the outcome of a single organism's virulence arsenal; rather, it is an ecological process determined at the community level.

The empirical basis for this view continues to expand. Large‐scale field surveys by previous researches revealed that the *Fusarium* complex associated with tomato wilt comprises at least 19 species, including *F. acuminatum*, *F. ennutatum*, and *F. nirenbergiae*, with marked geographic variation and widespread co‐infection [654, 655]. The emerging picture is not one of a single clonal pathogen invading a uniform host, but of a diverse, regionally structured assemblage of related and unrelated species co‐occupying the same disease niche (Figure 12). This also implicates that the same disease in different geographic regions may be driven by substantially different species combinations, a pattern that single‐pathogen diagnostics would fail to capture [656]. In soybean root rot, for example, *Fusarium*‐produced vitamin B6 enables *Phytophthora sojae* to evade soybean host resistance, strongly supporting the rationale for dual‐target management against both pathogens simultaneously [657]. These examples reveal a recurring pattern: the core pathogen does not act alone but recruits specific resident microbiota members to form a “disease‐facilitating consortium” whose collective virulence exceeds that of any single member [37, 651].

#### The mechanisms underlying pathobiome assembly and function operate across both chemical and physical dimensions

At the chemical level, pathogen‐secreted effector proteins do more than simply suppress plant immunity. Some exert direct antimicrobial activity that reshapes the surrounding microbial community in favor of the pathogen. VdAve1, an effector from *Verticillium dahliae*, is a well‐characterized example: it selectively kills antagonistic bacteria in the rhizosphere, thereby clearing ecological space for microbial taxa that are compatible with or beneficial to the pathogen [658]. Secondary metabolites serve a parallel function. Polyketides produced by certain *Fusarium* species act as both a phytotoxin and a selective antimicrobial agent. By inhibiting beneficial microorganisms and releasing ecological niches, polyketides promote the directional enrichment of co‐pathogenic taxa near the infection site, constructing a localized zone where pathogen allies are favored and competitors are suppressed [659]. Beyond these individual‐level mechanisms, effector proteins can also function as extracellular public goods within pathogen populations. Because effectors are secreted into the shared environment, hypovirulent strains or those with incomplete virulence gene clusters can benefit from proteins produced by their neighbors, collectively breaching host defenses that no single strain could overcome alone [660]. This cooperation maintains genetic diversity within the pathogen population while stabilizing its aggregate virulence capacity. The distinction matters that the community‐level virulence does not require every member to carry a full complement of virulence genes, only that the population as a whole produces the necessary arsenal.

Physical interactions add another layer of complexity. Bacteria that specifically attach to fungal hyphal surfaces can exploit the mycelial network as a dispersal corridor, moving through soil pores or plant intercellular gaps far more efficiently than they could alone. In at least one well‐documented system, bacterial colonization of fungal hyphae simultaneously upregulates toxin biosynthesis genes in the fungal partner, thereby coupling enhanced dispersal with increased virulence output [661]. Among congeneric or phylogenetically related pathogen species, co‐infection in the field is common [655, 662], and the interactions involved extend well beyond passive coexistence. Weaker pathogens may play defined roles at particular disease stages, contributing to early colonization or late saprotrophic exploitation of host tissues, while strong pathogens dominate active infection. This spatiotemporal partitioning allows the pathogen consortium to maintain continuous pressure on the host across the full disease cycle [662, 663].

Across both the chemical and physical dimensions described above, a recurring theme is that certain indigenous community members consistently facilitate pathogen infection. This common ecological pattern can be encapsulated by the definition of “disease‐facilitating microbes,” referring to resident microbial taxa whose abundance is positively associated with pathogen density and which enhance infection via chemical signaling or physical facilitation [664]. This framework is particularly valuable, as it shifts research focus away from individual pathogens toward the broader biotic interaction networks that drive disease emergence and spread. These individual‐level and community‐level mechanisms do not operate in isolation. Co‐infecting pathogens influence each other's virulence expression and population dynamics through both direct interference and host‐mediated feedbacks, and the consequences extend beyond the immediate infection event. Co‐infection generates selection pressure on virulence traits, creates opportunities for novel genetic combinations through horizontal gene transfer or reassortment, and maintains pathogen diversity within populations. At the epidemiological scale, multi‐infection can amplify the severity and reduce the predictability of disease outbreaks beyond what single‐pathogen models would anticipate. The central argument emerging from this body of work is that pathobiome virulence is a community‐level property rather than a trait encoded by any one genome. It is generated by the active restructuring of the rhizosphere micro‐environment through effector secretion, secondary metabolite production, and physical association, and sustained by the mutualistic networks that form between the invading pathogen and resident microbiota. This interpretation offers a straightforward account of a long‐standing puzzle in applied plant pathology: namely, why control strategies targeting a single pathogen frequently fail under field conditions. Removing the primary pathogen does not necessarily dismantle the cooperative microbial infrastructure that supported disease in the first place.

Several specific questions arising from this framework remain largely unresolved. The cross‐kingdom partners functioning as disease facilitators for major crop pathogens need systematic identification, and the chemical mediators and physical interfaces governing their cooperation require molecular‐level characterization [665]. Equally important, disease management strategies must move beyond single‐target intervention. Durable control is more likely when both the core pathogen and its key microbial allies, including cross‐kingdom disease‐facilitating partners, are disrupted simultaneously. In practice, this could involve designing synthetic communities that competitively exclude facilitating taxa, or developing targeted approaches that interfere with the specific molecules, such as effectors, secondary metabolites, or quorum‐sensing signals, that sustain cooperation within the pathobiome. There is also a temporal dimension to consider that even after the primary pathogen has been successfully suppressed, residual disease‐facilitating communities may persist in the rhizosphere and lower the colonization threshold for subsequent pathogen invasion, creating conditions for recurrent disease. This possibility reinforces the argument that management strategies should target the cooperative network as a whole rather than the core pathogen in isolation. Integrating pathobiome ecology into epidemiological modeling should further improve the capacity to anticipate disease outbreaks under variable environmental conditions and intensifying agricultural practice. The practical direction is clear in that effective management of plant diseases in complex field environments will increasingly depend on strategies that address not only the core pathogen but also the cooperative cross‐kingdom microbial network that enables its colonization, persistence, and virulence expression.

## DRIVING FORCES OF PLANT MICROBIOME COMMUNITY ASSEMBLY

**FIGURE 13:**
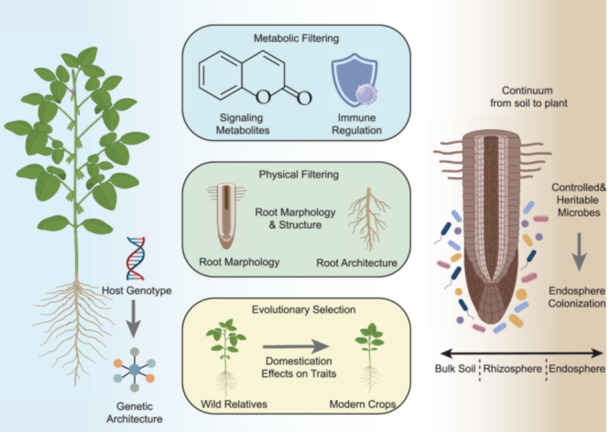
Conceptual framework of the driving forces and multi‐layered filtering mechanisms governing plant microbiome assembly. Plant microbiome assembly arises from the interplay between host‐mediated selection and stochastic environmental contingencies along the continuum from bulk soil to the endosphere. As microbial communities transition toward the host interior, selection pressure intensifies, progressively enriching for a subset of heritable, host‐regulated microorganisms within plant tissues. Metabolic filtering operates at the primary host–microbe interface, whereby signaling metabolites translate the host's physiological status into ecological cues that promote the recruitment or exclusion of specific microbial taxa. Concurrently, the plant innate immune system acts as a selective gatekeeper, balancing tolerance of beneficial symbionts with resistance to pathogens. Physical filtering is mediated by developmental and structural host traits; variation in root morphology, including root hair density and overall root system architecture, reshapes the microbial habitat template, with particular relevance during stress‐induced microbiome remodeling. From an evolutionary perspective, domestication has substantially altered these assembly processes, frequently eroding the capacity of modern crop varieties to sustain the diverse and functionally integrated microbial partnerships characteristic of their wild progenitors. Together, these interconnected selective dimensions define the boundary conditions underpinning the predictive design of plant‐associated microbiomes.

**FIGURE 14:**
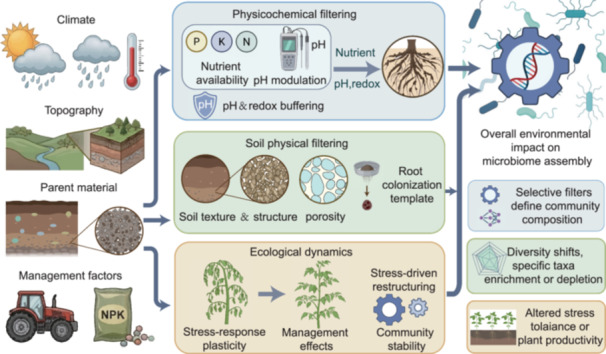
Environmental context defines the boundary conditions and filtering mechanisms governing plant microbiome assembly. Macroscopic environmental factors, including climate, topography, parent material, and anthropogenic management, collectively determine the regional microbial species pool and constrain root colonization potential. These drivers act through three interconnected filtering pathways: (1) Physicochemical filtering, whereby soil nutrient availability (N, P, K), pH, and redox conditions impose broad physiological constraints on microbial survival and modulate host metabolic plasticity; (2) Soil physical filtering, whereby texture, aggregate structure, and pore geometry establish the physical template for root‐microbe spatial interactions; (3) Ecological dynamics, encompassing management‐induced perturbations and stress‐driven community restructuring, which regulate assembly stability and host stress‐response plasticity. Together, these overlapping selective pressures drive compositional shifts, progressively enriching or depleting specific microbial taxa, that cumulatively determine microbiome structure and, consequently, plant stress tolerance and agricultural productivity.

Plant microbiome assembly emerges from the joint action of deterministic and stochastic processes. In community ecology, niche‐based mechanisms emphasize environmental filtering and biotic interactions, whereas neutral processes underscore the roles of dispersal and ecological drift [666, 667]. In plant‐associated systems, however, these processes are not uniformly distributed across compartments. Along the continuum from bulk soil to the rhizosphere, rhizoplane, endosphere, and phyllosphere, host‐mediated selection progressively strengthens, indicating that plants function not merely as substrates for microbial colonization, but as active biological filters [2, 5]. Although this hierarchical framework provides strong explanatory value at the community level, its mechanistic basis remains only partially resolved. In particular, host genetic modules imposing selection, metabolites translating host state into rhizosphere signals, and environmental contingencies modulating these effects remain incompletely understood. Here, we synthesize the major drivers of plant microbiome assembly across three interconnected dimensions: host genotype, metabolite‐mediated molecular interactions, and environmental context (Figures 13 and 14).

### Host genotype as a determinant of microbiome assembly

Host genetics is a fundamental factor in the assembly of host‐associated microbiomes, shaping microbial communities across generations through selective filtering processes. Although parental legacy (vertical transmission) often determines initial colonization, host genetic factors can override this influence, particularly in later generations, resulting in a host‐specific rather than purely inherited microbiome. While environmental factors (e.g., stress, soil properties, and agricultural practices) strongly influence community structure, host genetic variation—expressed through species‐specific genes and immune pathways—also helps determine microbial persistence. In particular, host genes involved in nutrient uptake, immunity, and root exudation contribute to this selective filtering.

### Heritability of plant microbiomes and their genetic architecture

A central question in plant microbiome research is how much variation in microbial community composition can be attributed to host genetic variation. Drawing from quantitative genetics, several studies have treated the abundance of individual microbial taxa as host‐associated traits and estimated their heritability. In maize, Peiffer et al. [50] found that while host genotype accounted for only a modest fraction of overall rhizosphere variation, specific operational taxonomic units displayed significant heritable patterns, suggesting that host control is concentrated in a genetically responsive subset of microbes rather than evenly distributed across the entire community.

GWAS have subsequently enabled the identification of host loci underlying such effects. In sorghum, Deng et al. [668] detected a genomic region on chromosome 4 associated with rhizosphere community variation and further identified heritable bacterial lineages shared with maize, suggesting that host control over portions of the microbiome may be evolutionarily conserved across grasses. Similar logic has been extended to aerial tissues. For example, Horton et al. [669] found in *Arabidopsis* that loci related to defense and cell wall integrity contribute to phyllosphere community structure. Host genetic control has also been documented in seeds, as evidenced by a recent Arabidopsis study that linked seed microbiome variation to host genomic background and identified key host determinants influencing the persistence of beneficial bacterial assemblages [104].

Taken together, these studies indicate that microbiome assembly is, at least in part, under host genetic control (Figure 13). Yet this control is modular, compartment‐specific, and taxon‐selective, rather than global. Importantly, association does not by itself establish mechanism. The microbiome acts as a high‐dimensional composite phenotype, and reducing community variation to individual taxa can overlook emergent properties and interaction effects. Moreover, genotype‐by‐environment interactions can obscure or reshape host effects in field settings. A key advance toward integrative analysis was demonstrated in foxtail millet, where GWAS, metabolite‐wide association study (MWAS), and microbiome GWAS (mGWAS) were combined to link host loci, metabolite variation, and microbiome structure within a unified framework [670]. Nevertheless, causal validation remains essential.

### Immune signaling and root architecture as selective mechanisms

Host genetic effects are mediated by biological systems that determine which microbes are admitted, excluded, or maintained in association with roots (Figure 13). The plant innate immune system represents a major filtering mechanism. Its role extends beyond pathogen defense, contributing to the structuring of beneficial and commensal assemblages. SA signaling modulates colonization by specific root‐associated bacterial taxa, suggesting that immune signaling contributes to defining the taxonomic boundaries of the microbiome [671]. Furthermore, the dual‐layered immune system comprising pattern recognition receptors and intracellular NLR (Nucleotide‐binding Leucine‐rich Repeat) receptors has been proposed to function as a selective gatekeeper, balancing tolerance to commensals with resistance to pathogens [672]. This balance is not static. Synthetic community experiments have demonstrated that commensal bacteria can actively modulate host immune output, revealing reciprocal feedback between immune calibration and community assembly [673].

Host genetic control is also exerted through developmental traits that shape the physical and chemical microhabitat of roots. Root hairs provide a clear example. In barley, root hair mutants display significant shifts in rhizosphere community composition, demonstrating that even subtle changes in root morphology can alter the microbial habitat template [674]. More recently, work under drought conditions has demonstrated that root hair developmental regulators contribute to stress‐triggered microbiome remodeling and strengthen interactions with beneficial Rhizobiaceae, linking developmental genetics with stress‐responsive microbial recruitment [675]. Therefore, host control over the microbiome is not only biochemical but also architectural, for example, plants shape microbial assembly through immune thresholds and through the physical organization of the root interface.

### Domestication as an evolutionary force reshaping microbiome assembly

Domestication offers an evolutionary framework for examining the long‐term coupling between host genome evolution and microbiome restructuring. Selection for yield, architecture, and agronomic performance has profoundly altered plant phenotype, but it may also have modified crops' ability to recruit and maintain beneficial microbial partners.

Evidence from plant systems supports this view. In common bean, wild accessions harbour more diverse rhizosphere microbiomes than domesticated lines, a difference associated with root phenotypic traits such as a more extensive root hair system [18]. Across independent domestication events in the Americas, Soldan et al. reported convergent microbiome shifts between wild and domesticated accessions [676], suggesting that domestication repeatedly remodels plant‐associated microbial communities in a convergent rather than idiosyncratic manner [676].

Domestication also affects microbial function. In tetraploid wheat, Yue et al. [677] reported a shift from fungal‐enriched rhizospheres with stronger carbon fixation signatures in wild relatives toward bacterial‐enriched communities with enhanced carbon degradation potential in domesticated forms. In rice, domestication has likewise been linked to reduced abundance of microbial nitrogen fixation functions and increased enrichment of genes associated with nitrous oxide emissions, suggesting that crop improvement may sometimes come at the expense of beneficial microbiome services [678]. Similar trait‐associated microbiome shifts have also been documented across tetraploid wheat domestication gradients [679]. Together, these findings have given rise to the concept of microbiome “re‐wilding,” wherein crop wild relatives serve as reservoirs of host traits and microbial partnerships that could be reintroduced into modern agriculture [680].

### Host gene–metabolite–microbe regulatory interfaces

The host gene–host metabolite‐recruited microbe regulatory interface represents a complex, multi‐directional communication system: host genetic factors and metabolites actively shape the composition and function of the microbiome, which in turn feeds back to modulate host gene expression, metabolism, immunity, and environmental stress responses [46]. This intersection, often mediated by small molecules, plays a crucial role in maintaining plant health and performance, driving disease, and influencing the evolution of host and associated microbiomes.

### Root exudates as the molecular interface between host and microbiome

Host genetic regulation of the microbiome must ultimately be translated into the soil environment through molecular intermediates, and root exudates are central to this process (Figure 13). Plants allocate a substantial fraction of fixed carbon belowground, with exudation acting as both a metabolic subsidy and a signaling system for surrounding microbes [12]. Conceptually, exudates operate at two functional levels. Primary metabolites such as sugars, amino acids, and organic acids broadly sustain microbial growth and activity, whereas specialized metabolites such as coumarins, benzoxazinoids, terpenoids, and flavonoids, exert more selective effects on community structure and function [681].

This distinction is useful, but not absolute, as some compounds perform both nutritive and selective functions. In Arabidopsis, root‐secreted malic acid can serve as a carbon source while simultaneously acting as a recruitment cue for beneficial *B. subtilis* [682]. Additionally, root exudation creates a fundamental ecological dilemma, since molecules that sustain beneficial microbes can also be intercepted by opportunists or pathogens. Therefore, root exudation is not merely a passive leakage of metabolites but rather a tightly regulated interface through which plants balance nutrient provisioning, selective microbial recruitment, and defense.

Importantly, exudate‐mediated recruitment should not be viewed as a simple presence‐ absence effect. Root‐derived compounds form concentration gradients around different root zones, with abundances varying by root type, developmental stage and spatial position along the root axis [46, 683]. These gradients can create local thresholds for chemotaxis, substrate utilization, growth promotion or inhibition, thereby generating spatial heterogeneity in microbial distribution. Dynamic exudate chemistry and microbial substrate preferences have been shown to jointly drive rhizosphere community assembly [11]. Recent studies further emphasize that stress‐induced modulation of root exudation should be interpreted in relation to microbial recruitment capacity and rhizosphere conditions [684, 685]. Therefore, concentration‐dependent recruitment provides a more realistic framework for linking root exudate chemistry to spatially structured microbiome assembly.

The signaling dimension of exudation is particularly evident in mutualistic interactions. Under nitrogen limitation, legumes increase flavonoid secretion, which induces rhizobial *nod* gene expression and initiates symbiotic signalling cascades [415]. Under phosphorus limitation, strigolactones are released to promote the recruitment and activation of AM fungi [686, 687]. These examples illustrate a broader principle: plants use exudates to translate internal physiological state into ecological cues that direct microbiome assembly and function.

### Causal evidence for metabolite‐mediated microbiome structuring

A major conceptual advance in this field has been the shift from correlation to causation. The most compelling studies have involved disrupting specific biosynthetic pathways and quantifying the resulting effects on microbiome composition and plant phenotype.

Coumarins provide one of the clearest causal systems. In Arabidopsis, MYB72‐dependent coumarin secretion does not just restructure the root microbiome; it also promotes iron mobilization and plant health [14]. Synthetic community experiments confirmed that loss of F6'H1‐dependent coumarin production alters root community composition under iron deficiency. Some bacterial strains that coumarins normally keep in check through redox activity end up enriched [688]. This framework was extended functionally when root‐secreted coumarins and the microbiota work together to improve iron nutrition in alkaline soils [689]. Therefore, coumarins do double duty: they help acquire nutrients and filter the microbial community at the same time.

Benzoxazinoids offer a parallel example in grasses. In maize, mutants that can't make benzoxazinoids show consistent shifts in rhizosphere and root microbiomes across multiple field soils, especially among fungi [690]. Later work found that bacterial tolerance to benzoxazinoid breakdown products predicts which bacteria can successfully colonize wild‐type maize roots. This indicates that plants use their own antimicrobials to shape the root microbiome by favoring compatible strains [691]. Importantly, benzoxazinoids also generate soil legacy effects: one generation's metabolites can change which microbes the next generation recruits. That links chemistry, microbiome assembly, and temporal inheritance in the field [692, 693].

Arabidopsis triterpenes reveal a complementary mechanism. Instead of just inhibiting microbes, triterpene networks create metabolic niches that favor specific taxa. Huang et al. showed that root‐specific triterpenes can either suppress or sustain particular bacteria, with some compounds even serving as sole carbon sources for distinct members of the microbiota [694]. This finding broadens the prevailing view of host filtering: plants do not simply exclude undesirable microbes, but can also actively construct biochemical niches to enrich their chosen partners.

### Shoot‐to‐root signals shape rhizosphere microbiome assembly

Root exudation and rhizosphere microbiome assembly are not regulated solely at the local level but they are tied into whole‐plant signaling. In Arabidopsis, shoot‐derived signals can modulate root camalexin exudation. That means what is happening above‐ground directly affects the chemical environment that root microbes experience [695]. Similarly, herbivory‐induced jasmonate signaling can alter root exudation profiles, reshape the rhizosphere microbiome, and generate plant‐soil feedbacks that influence subsequent above‐ground interactions [696]. These findings are conceptually important because they put microbiome assembly inside the plant's systemic stress physiology, not just in the root zone.

### Challenges in establishing host gene‐host metabolite‐microbiome causality

Despite substantial progress, rigorous causal inference remains difficult. A robust evidence chain should ideally proceed through four levels: namely, (i) identification of host loci associated with microbiome variation, (ii) loss‐ and gain‐of‐function studies of the relevant biosynthetic pathways in relation to microbiome variation, and (iii) reconstitution experiments showing that the resulting microbiome changes affect plant performance. In practice, most studies still concentrate on the first two steps. Functional validation is often limited by a lack of transformation systems in non‐model crops, the context‐dependent nature of field microbiome effects, and the difficulty of teasing out causality from high‐dimensional multi‐omics data [670, 677]. Going forward, accumulating more correlations will not cut it, we need experimentally tractable mechanistic chains.

### Environmental context as the boundary condition for host filtering

Host genetics and metabolism do not operate in isolation. They are embedded in environments that determine which microbes are available, what physical and chemical constraints they face, and how strong host filtering actually is. At small spatial scales, host genotype can measurably influence microbiome composition; across broader spatial gradients, however, soil properties, climate, and management practices often dominate community variation [48, 697]. The environment, therefore, sets the boundaries within which host‐dependent assembly unfolds (Figure 14).

The relative contributions of environmental filtering and host genetic control are context‐dependent rather than fixed. Field studies in maize showed that host genotype accounts for a modest proportion of total rhizosphere variation, although some microbial taxa display significant heritability [50, 698]. Similar host‐associated signals have been detected in sorghum and foxtail millet using GWAS and multi‐omics, linking host loci, metabolites, and microbiome variation under field conditions [668, 670]. However, soil physicochemical properties, climate, developmental stage, and management practices often dominate community variation across broader spatial scales [48, 697, 699]. Therefore, researchers have proposed distinguishing an environment‐dominated variable microbiome from a plant genetic‐dominated heritable microbiome. This distinction has practical value for crop improvement: breeding is unlikely to control the entire rhizosphere community, but might select stably for host‐responsive microbial subsets or functions that persist across soils, developmental stages and stress combinations. Maintaining genetically driven recruitment under field conditions will therefore need multi‐environment validation and genotype‐by‐environment analyses.

### Soil properties and host responses interaction shape microbiome composition and structure

Soil pH, nutrient availability, redox status, and texture all impose broad physiological filters on microbial communities [697]. Yet in plant microbiome research, the key issue is not simply that soil matters, but that soil conditions are often transduced through the host. In alkaline soils, iron deficiency induces coumarin biosynthesis, which in turn reshapes the microbiome through selective chemical filtering [689]. Under phosphorus limitation, enhanced strigolactone production promotes arbuscular mycorrhizal recruitment [687]. These cases illustrate that environmental effects are frequently mediated through host metabolic plasticity. Plants, therefore, act as biological relays that convert specific soil properties into microbiome assembly signals.

### Stress‐driven adaptive restructuring of microbiomes

Environmental stress alters host transcriptional and metabolic programs, which in turn changes the chemical environment of the rhizosphere. The resulting microbial community then feeds back on nutrient acquisition, stress tolerance, and plant performance [700]. This observation has led to the idea that the rhizosphere microbiome is part of the plant's stress‐response system, and might even retain a sort of ecological “memory” that helps with stress resilience [701]. A big unresolved question is whether such restructuring reflects active host recruitment of beneficial partners, passive sorting by environmental change, or, more likely, a combination of both. Resolving this issue is crucial for the rational design of stress‐adapted microbiomes.

### Management, time and space as dynamic modifiers of microbiome assembly

Agricultural management represents one of the major anthropogenic modifiers of plant microbiome assembly. Intensive management can reduce network complexity and deplete keystone taxa in root‐associated communities [702]. Similarly, pesticides can also harm beneficial symbionts like AM fungi [703]. These interventions act not only on microbial abundance but also on the ecological architecture of microbiome function.

Assembly is also dynamic across time and geography. Seasonal tracking of the *Populus* microbiome showed that temporal variation can exceed host genetic effects, highlighting the importance of development stage and shifting resource allocation [699]. Across the natural range of switchgrass, host genotype retained detectable and repeatable effects on root‐associated microbiota despite strong environmental turnover [704]. These findings indicate that microbiome assembly is simultaneously contingent and structured: shaped by local context, yet constrained by host‐associated regularities that may prove exploitable for breeding or engineering.

### Multi‐factor interactions and future directions

Plant microbiome assembly should therefore be viewed as an emergent property of interacting host genotype, metabolite‐mediated signaling, and environmental context. These layers are nested rather than independent. Environmental stress alters host transcription and metabolism, which changes the chemical environment of the rhizosphere, and then the microbial community feeds back on nutrient acquisition, stress tolerance, and plant performance [2, 5]. Therefore, the microbiome is best understood not as a passive consequence of plant growth, but as an active component of plant ecological strategy.

Several big gaps now define the frontier of the field. First, complete causal pathways remain rare: very few studies connect host genetic perturbation to metabolite change, microbiome restructuring, and plant phenotype within a single framework. Second, higher‐order interactions (genotype × metabolite × environment) remain poorly resolved. Third, many mechanistic insights still derive from Arabidopsis and a few cereals; we do not know how well they transfer to legumes, perennials, and other crops. Fourth, temporal processes, including successional shifts across development and intergenerational soil legacy, have not yet been fully integrated into theory.

The next phase of plant microbiome research needs to move from descriptive profiling to predictive and design‐oriented biology. Precision genome editing can accelerate causal testing in non‐model crops. Spatial metabolomics and spatial transcriptomics will let us resolve microbiome assembly at the microscale where roots actually release metabolites. Meanwhile, AI‐enabled frameworks are starting to offer predictive tools for microbiome modeling, synthetic community design, and even digital‐twin integration of plant and microbiome data [705]. The long‐term challenge is no longer simply to describe which microbes are present in the microbiome, but to understand when, why, and under what constraints we can actually design microbiome assembly.

## METHODOLOGIES THAT ARE REVOLUTIONIZING PLANT MICROBIOME RESEARCH

To test the hypotheses generated from our understanding of community assembly drivers and to translate this knowledge into applications, we require the sophisticated toolkits. The following section provides an overview of the cutting‐edge methodologies that are revolutionizing plant microbiome research, from quantitative profiling to synthetic community design and advanced statistical analyses.

### Quantitative microbiome

Quantitative Microbiota Profiling (QMP) represents a fundamental paradigm shift in microbial ecology, overcoming the critical limitations of relative abundance analysis, which still dominates the vast majority of microbiome research [88, 706]. Relying on proportional data suffers from inherent compositional bias, lacks absolute quantity information, and constrains the resolution of true ecological interactions, often masking the underlying biology of microbial communities [707, 708]. To address this, QMP methodologies allow precise measurement of absolute microbial abundances, moving beyond proportions to quantify the actual number of cells or gene copies per sample unit. Core techniques include traditional CFU counting, targeted quantitative PCR (qPCR), high‐throughput flow cytometry, and the transformative spike‐in method (Table 1). The spike‐in approach, which adds a known quantity of exogenous cells or DNA as an internal standard, is especially effective at correcting technical biases across the entire sequencing workflow, thereby converting standard relative sequencing data into reliable absolute abundance data [709, 710]. More recently, methods such as Accu16S/AccuITS [711], HA‐QAP [712], alongside spike‐in and rrnDB‐informed absolute quantitation [710], have been developed for accurate absolute microbiome quantification. Comparative studies have demonstrated the higher sensitivity of methods like droplet digital PCR (ddPCR) [713] and flow cytometry [714]; the use of spike‐in standards with sequencing has been instrumental in challenging old ecological models and establishing QMP as an indispensable discipline for deciphering complex microbial ecosystems.

**TABLE 1:** Comparison of methods for absolute quantification in plant microbiomes.

Method	Core principle	Key advantages	Major limitations	Notes for host‐associated microbiomes (*e.g*., plant endophytes)
Spike‐in Internal Standard	Adding a known quantity of exogenous DNA (spike‐in) to the sample prior to DNA extraction. Absolute abundance is calculated from spike‐in recovery in subsequent sequencing data (16S/Shotgun).	Community‐wide absolute quantification. Corrects for technical biases across the entire workflow. Seamlessly integrates with taxonomic/functional profiling.	Critical dependence on spike‐in properties (must mimic native cell lysis & amplification). Increases experimental cost and data analysis complexity. Cannot distinguish viable cells.	The preferred high‐throughput solution. Directly addresses host DNA interference, enabling absolute abundance profiling of the entire community.
Quantitative PCR (qPCR)	Target‐specific amplification with fluorescent probes. Quantification against a standard curve to determine absolute gene copy number.	High specificity and sensitivity for targeted taxa/genes. Mature and standardized.	Low throughput (limited targets per run). Primer bias and potential off‐target amplification. Cannot distinguish viable cells.	Use with extreme caution. Universal primers (*e.g*., 16S) co‐amplify host organelles (chloroplast/mitochondria). Requires highly specific primers (*e.g*., for single‐copy housekeeping genes) validated against host DNA.
Colony Forming Unit (CFU) Count	Serial dilution and plating on culture media. Counting of visible colonies after incubation.	The gold standard for enumerating cultivable, viable cells. Simple, intuitive, and yields isolates for downstream study.	Extremely low throughput and time‐consuming. Severely underestimates total diversity (>99% uncultivated). Selective bias based on media and conditions.	Applicable only for the cultivable fraction. Useful for obtaining isolates but does not represent the in‐situ community. Results are complementary to molecular data.
Flow Cytometry	Fluorescent staining of cells in liquid suspension and high‐throughput single‐cell detection/counting.	Very rapid total cell counts (viable and total). Can assess cell physiological status.	Low taxonomic resolution (requires coupling with FISH for identification). Complex sample preparation for solid matrices to remove debris.	Challenging for solid tissues. Can estimate total microbial load in tissue homogenates but requires extensive optimization to separate microbes from host debris.
(Epifluorescence) Microscopy	Direct visualization and manual counting of fluorescently stained cells on a slide.	Most direct and visual absolute count. Provides morphological and spatial context.	Extremely low throughput, labor‐intensive, and subjective. Low taxonomic resolution.	Impractical for most host samples. Difficult to reliably distinguish microbial cells from host cell fragments and auto‐fluorescent material.
Automated Cell Counter	Electronic impedance or image‐based counting of particles/cells passing through a microaperture.	Simple and rapid concentration and size measurements.	Cannot distinguish microbial cells from abiotic particles/debris. Provides no biological information.	Not recommended for complex samples. Significant overestimation due to host particles. Only suitable for pure cultures or very simple suspensions.

*Note*: The Spike‐in Internal Standard method is currently the most robust and informative high‐throughput approach for obtaining community‐wide absolute abundances, as it directly corrects for the overwhelming host DNA background. qPCR is reliable only for tracking specific taxa with rigorously validated primers. CFU counts remain valuable for cultivating and quantifying the viable, cultivable subset. An integrated approach using Spike‐in (community profile) and qPCR/CFU (target validation) is often the strongest strategy.

The application of QMP has led to new discoveries across diverse ecosystems by providing an unambiguous, quantitative lens. In plant rhizosphere research, combining spike‐in standards with sequencing revealed that bacterial abundance is higher in the rhizosphere soil than in bulk soil or roots, leading to the proposal of a new “amplification‐selection” model for community assembly that challenged the previous “two‐step recruitment” model based on relative data [710]. Similarly, quantitative abundance profiling has revealed microbial load variation in the soil and root microbiomes [381, 715]. Further quantitative studies showed that under low‐nitrogen stress, the absolute abundance threshold of rhizosphere nitrogen‐fixing bacteria strongly correlated with soybean nitrogen use efficiency [716], and that AM fungal colonization increased rhizobial absolute abundance, revealing biomass‐mediated symbiotic networks [381]. In animal gut microbiota, a landmark study identified microbial load (total bacterial cells per gram) as the primary discriminator between health and disease, with Crohn's disease patients showing a three‐fold reduction compared to healthy controls [708]. QMP also resolved ecological artifacts, showing that the purported reciprocal *Prevotella‐Bacteroides* relationship was actually a synergistic reduction under inflammation [708], and linked specific reductions in gut bacterial abundance to conditions like osteoporosis [717]. In environmental microbiomes, the within‐ and between‐functional redundancy degrees quantified that soil microbial functional redundancy is deterministically regulated by temperature and pH [718], while in aquatic systems, absolute abundance thresholds for ammonia‐oxidizing archaea and nitrite‐oxidizing bacteria serve as early warning indicators for nitrogen cycle imbalance [719].

The precision of QMP unlocks transformative application prospects across multiple fields. In medical diagnostics and therapy, it improves the detection sensitivity of tissue‐specific microbial differences, as seen in hypopharyngeal squamous cell carcinoma, where it boosted sensitivity compared to relative profiling [720], and helps clarify therapeutic mechanisms, such as how a microbiota‐dependent metabolite alleviates insulin resistance [721]. For sustainable agriculture, QMP guides soil management by linking the absolute abundance of specific microbial taxa to soil health indicators; for example, agricultural reclamation in the Tengger Desert increased total bacterial load by nearly two orders of magnitude, with certain groups' abundances positively correlated with soil organic carbon [722]. It also enables the screening of effective biocontrol strains and the optimization of silage additives [723]. In environmental remediation, QMP allows for the optimization of strategies by correlating degrader cell density with pollutant breakdown rates, such as in a marine bacterial consortium degrading polyethylene, where five bacterial general dominated and enzyme activity correlated with its density [724], and quantifies pathogenic risks, like the elevated antibiotic resistance gene load in microplastic‐surface biofilms [725]. Furthermore, in industrial contexts like food fermentation, it systematically characterizes microbial structure and metabolite dynamics to optimize processes [726].

**FIGURE 15:**
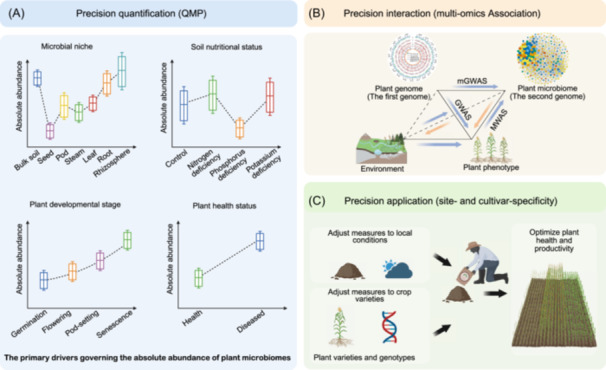
A research‐to‐application framework for the precision agricultural microbiome. (A) Precision Quantification via Quantitative Microbiome Profiling (QMP). This approach quantifies absolute microbial abundance, with key determinants including plant developmental stage, soil nutrient status (*e.g*., phosphorus deficiency), distinct microbial niches (*e.g*., bulk soil, rhizosphere, and endosphere), and plant health. A line graph illustrates the dynamic change in endophytic microbial load across the plant life cycle, shaped by these factors. (B) Precision interaction via GWAS‐MWAS‐mGWAS integration. A core multi‑omics paradigm decodes plant‐microbiome relationships by treating the microbiome as the plant's “second genome”, linking the host genome (first genome) to the phenome. The workflow combines microbiome‑wide association studies (MWAS) to identify phenotype‑associated microbes, and microbiome genome‑wide association studies (mGWAS) to map host genetic loci that regulate these key taxa, with environmental factors modulating these associations. (C) Precision application based on “site‐specificity” and “cultivar‐specificity”. Translating fundamental insights into practice, this strategy enables tailored microbial management. Guided by the principles of site‑ and cultivar‑specificity, it involves the rational selection and application of SynComs or beneficial consortia to optimize plant health and fitness, thereby closing the loop from discovery to field implementation.

The future of QMP is likely to see further breakthroughs through the integration of single‐cell technologies and multi‐omics analyses, promising new solutions for precision medicine, sustainable agriculture, and environmental protection. Crucially, absolute abundance is highly sensitive to environmental perturbations, exhibiting significant fluctuations even when relative abundance remains stable. To accurately resolve spatiotemporal dynamics—spanning the rhizosphere interface, soil nutrient status, plant developmental stages, and health conditions—we advocate for a shift toward absolute abundance‐based QMP. This approach is imperative to elucidate the true ecological functions of plant microbiomes and enable the precision deployment of microbial inoculants (Figure 15A).

### Agricultural precision microbiome

Precision Agricultural Microbiome Engineering (PAME) represents a transformative shift in leveraging plant‐microbiome interactions for sustainable crop improvement. This concept, first proposed through the combinatorial analysis of genome‐wide association studies (GWAS), microbiome‐wide association studies (MWAS), and microbiome genome‐wide association studies (mGWAS) method (Figure 15B) using foxtail millet as a model, is founded on the principle that a plant's genotype is a key driver in shaping its associated microbial communities [670]. Research across various crops, including maize, tomato, and barley, has consistently identified heritable microbial taxa, confirming that plants can genetically control the assembly of specific segments of their microbiome [727, 728]. To strategically exploit this, a framework of Precision Agriculture Microbiome Engineering (PAME) has been proposed to dissect the rhizosphere microbiome into environment‐dominated and plant genetics‐dominated components, allowing targeted identification of microbial groups under host genetic control [729]. The core of PAME involves identifying the plant genetic basis behind the recruitment of these beneficial, heritable microbes. This is achieved through mGWAS, which pinpoints specific plant genes or loci, termed “*M* genes”, responsible for assembling functional microbiota. For example, in maize under nitrogen stress, a gene (*Zm00001d048945*, encoding a TPX2 (Targeting Protein for Xklp2) protein related to the WAVE‐DAMPENED2) involved in microtubule organization was found to recruit the beneficial bacterium *Massilia* to enhance growth [727]. The *M* gene concept has been further refined into Microbiome‐shaping (*Ms*) genes and Microbiome‐responsive (*Mr*) genes to better distinguish plant‐driven microbial assembly from plant responses to microbial cues [19]. The ultimate goal of PAME is to use this genetic knowledge to precisely manage the microbiome, either by editing host *M* genes or applying tailored microbial inoculants, to enhance crop traits like stress tolerance and yield [20, 728].

The implementation of PAME follows a systematic workflow that integrates advanced analytics to bridge discovery with application. The process begins with establishing robust correlations between plant genotypes, their microbiomes, and agronomic phenotypes within diverse genetic populations. The next critical step is the identification of “keystone” or “core” microbial taxa that are not only heritable but also exert a beneficial functional influence on the host plant. Moving beyond traditional correlation networks, researchers are increasingly employing sophisticated computational tools. For example, weighted correlation network analysis (WGCNA) has been used to identify *Massilia* as a key genus driving microbiome assembly under nitrogen deficiency [727]. Furthermore, machine learning and deep learning models are being harnessed to predict plant phenotypes from microbiome data, identify keystone species within complex communities, and design synthetic microbial consortia with desired functions [35, 675, 730]. Following microbial identification, mGWAS is deployed to rapidly map the plant genetic loci controlling the abundance of these key microbes [670, 729]. The final stage translates this knowledge into practical interventions. This can involve developing SynComs based on the identified core beneficial taxa, as demonstrated in soybean, where specific SynComs improved growth and nutrient uptake [730, 731], or leveraging the identified *M* genes for gene editing and microbiome breeding to create plant varieties optimized for beneficial microbial partnerships [19].

Recent progress in PAME has been driven by holistic and dynamic perspectives that account for the multifaceted nature of plant–microbiome–environment interactions. A “holo‐omics” approach, which integrates host‐derived data (genomics, transcriptomics, metabolomics) with microbiome profiles, is crucial for unraveling the functional mechanisms of these interactions [732]. For example, studies have shown that root exudates like benzoxazinoids shape the rhizosphere microbiome, which in turn provides feedback on plant growth and defense [692]; and that host metabolites and transporter genes (e.g., *SWEET*) create spatial niches for specific microbial colonization along the root axis [733]. Concurrently, a spatiotemporal understanding is essential for effective application. Microbial communities change dynamically across plant developmental stages and different plant organs, indicating that the efficacy of microbial interventions likely depends on timing and location [631, 675, 734]. Moreover, the environment exerts a strong influence. While core, heritable microbiomes exist, their composition and effect are modulated by soil type, climate, and agricultural management [735, 736]. This emphasizes the importance of developing environmentally adaptable SynComs or identifying universal core microbes that function across diverse conditions, as seen with conserved diazotrophic bacteria in maize xylem sap [737]. Furthermore, agricultural practices like intercropping and rotation can be designed to foster beneficial microbiome assemblies that suppress disease or enhance nutrient cycling [738, 739].

Future directions point towards dynamic, quantitative microbiome profiling and the design of SynComs that can adapt to environmental changes, moving PAME from a static concept to a responsive management tool for precision agriculture. Central to this approach is the translation of basic science into actionable strategies: leveraging site‐ and cultivar‐specificity to rationally select and apply microbial consortia. This targeted intervention optimizes plant fitness and completes the continuum from laboratory discovery to agricultural application (Figure 15C).

### Synthetic microbial community and microbiome engineering

**FIGURE 16:**
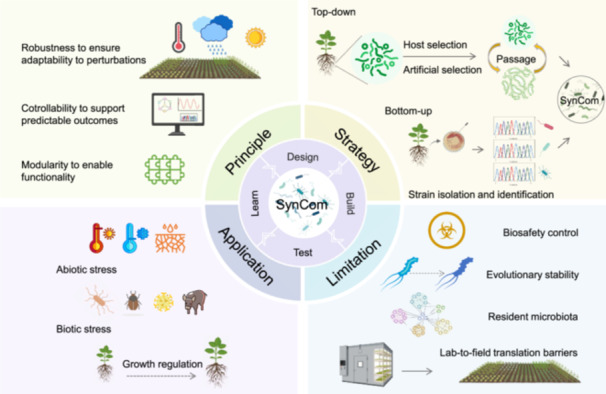
Conceptual framework for the design, construction, and application of plant‐associated SynComs. SynComs are designed to regulate plant growth and health by integrating ecological principles with synthetic biology tools, thereby bridging the gap between laboratory design and field application. The engineering of effective SynComs is guided by three core principles: modularity to enable specific functions, controllability to improve reproducibility and facilitate predictable outcomes, and robustness to ensure adaptability to environmental perturbations. SynComs construction follows two main strategies: the top‐down approach, which involves host‐mediated or artificial selection over successive passages to enrich for beneficial communities from a complex inoculum, and the bottom‐up approach, which relies on the individual strains with defined traits to assemble a consortium rationally. Successful deployment must address critical challenges that create a gap between laboratory design and field application, including but not limited to biosafety, resistance from native microbiota, and evolutionary stability.

The plant microbiome contributes significantly to plant growth and tolerance to biotic and abiotic stresses [2]. However, it is challenging to use these native communities consistently in agriculture due to their high complexity and frequent compositional variation across environments and host genotypes [38]. To tackle this challenge, researchers have increasingly focused on SynComs (Figure 16). SynComs simplify plant–microbiome systems while preserving key ecological and functional features, serving as useful tools for mechanistic studies and microbiome engineering [740, 741].

A SynCom is a strain‐defined assembly of culturable microorganisms with known membership and controlled inoculum ratios, typically reconstructed from a collection of plant‐associated isolates [740, 741]. Because SynComs reduce community complexity and standardize the starting input, they allow controlled perturbation experiments, including drop‐out, add‐back, and ratio‐titration tests, in which specific strains are removed, added back, or varied in relative abundance, respectively. These experiments facilitate linking community composition and microbial interactions to plant phenotypes. SynComs also connect reductionist microbiology with community ecology by allowing direct tests of concepts such as niche complementarity, functional redundancy, and priority effects [538, 742]. However, host filtering, resident microbiota, and environmental variation can all reshape SynCom assembly and activity, indicating that the defined community is not necessarily stable. Therefore, SynComs are best regarded as tools for mechanism discovery and as prototypes for engineering purposes. Any agricultural application would therefore further require ecological validation, suitable formulation, and effective delivery strategies to ensure reliable performance under field conditions.

### The construction principles of SynComs

A key goal in SynCom research is to shift from empirical testing toward designing communities with more predictable functions and more stable assembly. This goal is commonly framed around three principles: modularity, controllability, and robustness [743, 744]—concepts rooted in systems engineering and have been further adapted in synthetic biology for the rational design of complex biological systems (Figure 16). Together, these principles help clarify whether a design aims to optimize function, assembly, or long‐term persistence.

Modularity means the combination of several strains so that different members contribute different tasks to the whole community [745]. Complex community‐level functions that are challenging for a single strain to accomplish can be achieved through this division of labor. Additionally, because strains or functional modules can be added, removed, or changed as needed, modularity makes SynCom design easier to modify. In this way, modularity facilitates the transition from trial‐and‐error to a more methodical design process in SynCom construction [746].

Controllability is another important aspect of SynComs. The fact that SynComs usually consist of a limited number of strains enables researchers to monitor the community. Under controlled experimental conditions, this lower complexity often leads to better reproducibility and clearer functional readouts. In addition, combined with synthetic biology and mathematical modeling, controllability can further improve prediction and support iterative community design based on measured performance [747, 748].

Robustness refers to the ability of a SynCom to maintain function under changing environmental conditions. In practice, robustness depends on both community interactions and functional diversity. Researchers therefore often select and combine strains that can coexist through compatible relationships, including cross‐feeding and other stabilizing interactions [749]. In addition, adaptive evolution under selective pressure can sometimes enhance robustness as well, although it may also alter community composition and function in unexpected ways. Despite these risks, this evolutionary flexibility may sometimes help SynComs buffer environmental disturbances and maintain useful functions over time.

### Construction strategies of SynComs

Two main strategies are widely used to construct SynComs (Figure 16). Top‐down selection enriches communities from natural microbiomes under defined pressures, whereas bottom‐up assembly rebuilds strain‐defined communities from isolates [108]. In many cases, these two strategies complement each other. Top‐down studies can identify candidate taxa or interaction patterns in ecologically realistic settings, such as suppressive soils or stress‐shaped rhizospheres. Bottom‐up reconstruction can then be used to test causality, resolve mechanisms, and refine community composition under controlled settings.

### Top‐down enrichment and microbiome selection

Top‐down approaches begin with complex microbiota and apply ecological filters, such as host selection, environmental stress, pathogen challenge, or serial passaging, to enrich communities associated with desirable traits. The resulting consortium may be simplified, but it is not always fully defined at strain level. For example, in cereals such as wheat and maize, repeated planting under biotic pressure has been reported to enrich for beneficial taxa associated with improved host performance [750]. Likewise, multi‐generation microbiome selection in *Brassica rapa* has been used to shift rhizosphere communities toward improved plant traits [751]. These simplified native consortia, selected through stress or host pressure, are examples of SynComs assembled using a top‐down approach. A major strength of top‐down selection is that it preserves ecological context, including redundancy, interaction networks, and host feedback. This feature may help retain interaction structures that support community stability. However, top‐down systems are often harder to interpret and may show limited reproducibility across soils or resident microbiomes. Sequencing, strain isolation, and reconstruction are often needed to identify causal members and convert discovery‐based consortia into designable SynComs.

### Bottom‐up rational assembly and the Design‐Build‐Test‐Learn (DBTL) cycle

Bottom‐up construction starts with defined isolates and assembles them to reconstitute target functions and enable causal analysis. Candidate strains may be selected on the basis of traits, ecological roles, or network position, including possible keystone or connector taxa. Researchers often narrow these candidates through pairwise interaction assays and colonization tests to identify compatible combinations that can coexist stably [740, 748, 752, 753]. An iterative DBTL cycle is increasingly used in this process. In the design phase, strain sets and starting ratios are chosen using ecological reasoning, metabolic models, or interaction data. During the build phase, strains are combined into defined communities with controlled ratios. The test phase then evaluates community function and persistence across a range of systems, from in vitro assays to gnotobiotic plants, greenhouse experiments, and field trials when possible. In the learn phase, profiling and multi‐omics data help infer the interaction mechanisms, such as metabolite exchange, antagonism, immune modulation, and colonization bottlenecks, which then guide the next round of redesign [743, 748, 754]. Bottom‐up SynComs are highly interpretable, but they often perform less well in open soils unless robustness is built into the design process from the start and ecological constraints, including resident competition, abiotic variation, and priority effects, are explicitly considered.

### Recent advances in SynComs for enhancing plant stress resilience

In recent years, a growing number of studies have highlighted the potential of SynComs to enhance plant resilience against biotic and abiotic stresses, showing that they can help plants withstand a range of challenges and thus offer a promising strategy for crop protection [5, 730, 755, 756, 757]. Beyond these demonstrations of improved plant performance, researchers are making headway in understanding how such benefits arise.

Under biotic stress, several studies have shown that SynComs can suppress pathogens and pests through both direct and indirect mechanisms. For example, in susceptible cucumber challenged by root‐knot nematodes (RKNs), a keystone strain *Rhizobium pusense* TYQ1 showed marked enrichment in response to nematode stress. This strain acted against RKNs directly while also remodeling the rhizosphere bacterial community. Furthermore, the TYQ1‐centered SynCom showed enhanced biofilm formation and metabolic networking, helping to stabilize the community and strengthen nematode suppression [758]. In tomato, cross‐kingdom SynComs composed of both bacterial and fungal isolates from the rhizosphere exhibited superior suppression of Fusarium wilt compared to single‐kingdom consortia. This enhanced efficacy was associated with complementary functions involving plant immune modulation and inter‐kingdom microbial interactions [665]. Since then, mechanistic studies have shed light on how such bacterial‐fungal alliances function. In a similar inter‐kingdom SynCom composed of bacterial and fungal isolates, surfactin produced by *Bacillus velezensis* triggers protective azaphilone production in *Trichoderma guizhouense*, while fungal degradation of fusaric acid alleviates bacterial inhibition, collectively suppressing *Fusarium oxysporum* [759].

Beyond biotic stress management, SynComs also represent a promising approach for mitigating abiotic stresses, as illustrated by the case of drought. A global study of wheat rhizosphere microbiota revealed that drought stress enriches a set of drought‐tolerant bacteria (DTB) carrying genes for nutrient cycling, phytohormone production, and osmolyte synthesis. A SynCom composed of four of these DTB significantly improved wheat growth under water deficit, highlighting conserved microbial strategies to support plant fitness across environments [755]. Another recent study isolated a core set of drought‐responsive strains from the desert shrub *Caragana korshinskii*, where extreme aridity has fostered a resilient microbiota. A five‐member SynCom assembled from these isolates markedly increased wheat drought tolerance compared with monocultures; this effect was largely attributed to enhanced collective biofilm formation, which promoted root colonization and activated plant MAPK and JA signaling pathways involved in stress response [760].

### Section summary

A central barrier to agricultural application is that SynComs often lack the ecological robustness to perform reliably outside controlled environments. SynComs that perform consistently in gnotobiotic or greenhouse systems may lose members, shift in composition, or lose efficacy after introduction into heterogeneous field soils. Such failures are often driven by ecological filtering from resident microbiota, including priority effects (e.g., niche pre‐emption) and resource competition, as well as by abiotic variation that alters microbial interactions [538, 748, 761]. Stochastic drift and historical contingency further reduce predictability, while evolutionary dynamics may erode designed functions, posing additional challenges for long‐term reliability of the SynComs [762].

Bridging the laboratory‐to‐field gap requires ecologically informed design rather than simple scale‐up. In this context, AI‐assisted design can serve as a prioritization layer that translates ecological principles into testable SynCom candidates. Data‐driven models can integrate strain‐level data and environmental metadata to narrow the combinatorial search space of community membership and inoculation ratios. This may help identify formulations more likely to establish and persist across variable field conditions [763]. Importantly, such predictions should be coupled with iterative experimental validation to avoid overfitting to controlled systems. Building on ecological principles and informed by AI‐assisted prioritization, promising directions to improve field deployment include: (i) designing for establishment by aligning inoculation timing and delivery with early colonization windows (*e.g*., seed/seedling stages) and, where appropriate, pairing SynComs with supportive carriers or prebiotic amendments; (ii) incorporating niche complementarity alongside targeted redundancy to buffer environmental variation and member loss; and (iii) moving from one‐size‐fits‐all consortia toward modular formulations tailored to crop genotype and soil context [748, 764]. These efforts should be supported by benchmarking across representative soils and seasons, using standardized measures of both persistence and functional stability. From this perspective, introduced SynComs can be treated as ecological invaders, and concepts from invasion ecology and community assembly theory may provide useful guidance for deployment. Based on these ecological insights, AI‐driven SynCom design can translate design rules into optimized, field‐ready formulations.

In the future, synthetic biology could further enhance the ecological robustness of SynComs by enabling more programmable interactions through tools such as quorum‐sensing control, regulated antimicrobial production, and engineered metabolite exchange. Such approaches, however, will need to account for evolutionary dynamics that might erode designed functions, as well as biosafety considerations that accompany the release of engineered strains into open environments. In parallel, advances in multi‐omics, mechanistic modeling, and machine learning should help address these challenges by enabling AI‐driven SynCom design that accelerates predictive community construction and tailors deployment strategies to specific agronomic contexts [35, 765]. Nevertheless, AI‐driven SynCom design is currently constrained by the low level of data standardization and the difficulty of integrating heterogeneous datasets generated across different laboratories, a limitation that has prompted recent calls for community standards, benchmark datasets, and shared infrastructures for SynCom strain and data exchange [766]. Differences in host genotype, soil type, experimental protocols, sequencing platforms, inoculation methods, and phenotyping strategies can introduce strong batch effects and limit model transferability [766]. Moreover, because many AI models are trained primarily on controlled laboratory or greenhouse datasets, they may overemphasize short‐term community functions while insufficiently capturing long‐term persistence, colonization stability, and functional durability under field conditions [2, 38]. Together, advances in synthetic biology, multi‐omics, mechanistic modeling, and AI point to a future where SynComs can be engineered not just for function, but for ecological reliability and field application, ultimately unlocking their potential for sustainable agriculture.

## TECHNOLOGICAL AND ANALYSIS METHODS FOR PLANT MICROBIOME RESEARCH

**FIGURE 17:**
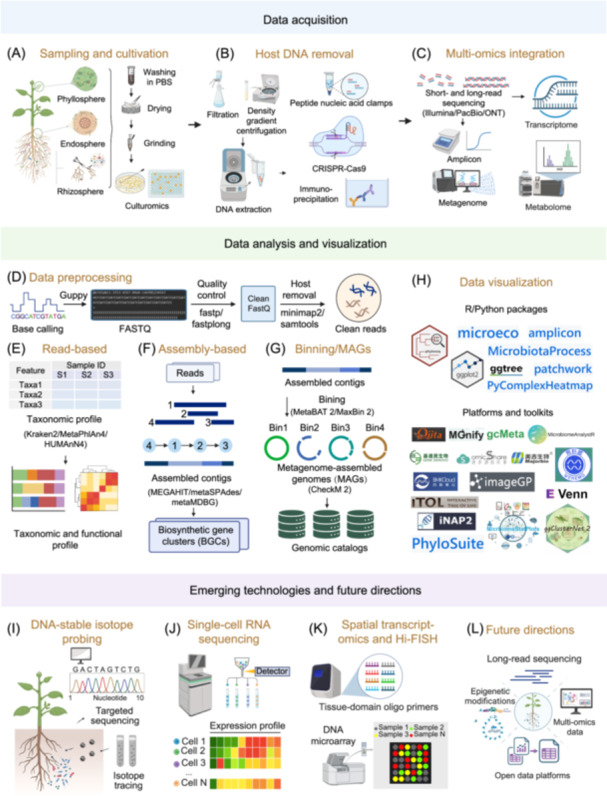
Analytical framework for plant microbiome. This schematic illustrates the full workflow from data acquisition to emerging technologies. (A) Sampling and cultivation: Samples are collected from major plant‐associated niches, including rhizosphere, phyllosphere, and endosphere, followed by washing, drying, grinding, and cultivation. (B) Host DNA removal: Host contamination is reduced using filtration, density gradient centrifugation, peptide nucleic acid clamps, CRISPR‐Cas9, and immunoprecipitation. (C) Multi‐omics integration: Short‐ and long‐read sequencing platforms (e.g., Illumina, PacBio, and ONT) are used to generate amplicon and metagenomic data, complemented by transcriptome and metabolome. (D) Data preprocessing: Raw FASTQ files undergo base calling and quality control (e.g., fastp and fastplong), followed by host read removal using tools such as minimap2 and samtools to obtain clean reads. (E) Read‐based analysis: Taxonomic and functional profiling is performed using tools such as Kraken2, MetaPhlAn4, and HUMAnN4. (F) Assembly‐based analysis: Reads are assembled into contigs using assemblers (e.g., MEGAHIT, metaSPAdes, and metaDBG), enabling the identification of biosynthetic gene clusters (BGCs). (G) Binning/MAGs: Assembled contigs are binned into metagenome‐assembled genomes (MAGs) using tools such as MetaBAT 2 and MaxBin 2, with quality assessment by CheckM 2, leading to the construction of genomic catalogs. (H) Data visualization: Results are visualized using R and Python packages (e.g., phyloseq, amplicon, microeco, ggplot2, and PyComplexHeatmap) and integrated platforms and toolkits (e.g., Qiita, MGnify, gcMeta, Wekemo Bioincloud, and ImageGP). Emerging technologies: These include DNA stable isotope probing (DNA‐SIP; I), single‐cell RNA sequencing (J), and spatial transcriptomics combined with Hi‐FISH for high‐resolution functional and spatial analysis (K). (L) Future directions: Advances in long‐read sequencing, epigenetic modification analysis, multi‐omics integration, and open data platforms will further enhance plant microbiome research.

High‐throughput metagenomics, sequencing technologies, and integrated multi‐omics approaches constitute the core foundation for unraveling the complex mechanisms underlying plant‐microbiome interactions [767]. Optimized sample preparation protocol and cutting‐edge sequencing methods not only enable precise identification of key microbial taxa and clarification of their functions, but also help address challenges posed by microbial “dark matter” (uncultured and uncharacterized microorganisms) within plant microbiomes [768]. Furthermore, standardized bioinformatic pipelines, R software packages [769], visualization tools, and cloud platforms are widely applied for the in‐depth analysis and systematic mining of plant microbiome data, transforming massive genomic datasets into biologically meaningful insights closely associated with plant nutrition and health (Figure 17A–L).

### Methods for efficient acquisition of plant microbiome data

Efficient acquisition of plant microbiome data is a prerequisite for accurately characterizing plant‐associated microbiome. Rhizosphere soil easy to accessible and exhibits a high microbial content, yet it remains soil. The phyllosphere and endosphere are the most activity region of plant–microbiome interaction; however, host DNA contamination poses a major challenge for direct sequencing [770] (Figure 17A). In these niches, plant‐derived DNA, including chromosomes, chloroplasts, and mitochondria, often dominates sequencing libraries, frequently accounting for more than 90% of total reads. This highly host DNA contamination masks microbial signals and reduces effective sequencing depth [593, 770]. Such contamination not only limits the recovery of microbial DNA [771], but also increases the complexity of downstream analyses.

To address this issue, various host DNA depletion and microbial enrichment strategies have been developed across multiple stages of sample processing. During sample preparation, physical methods such as filtration and density gradient centrifugation are widely used to enrich microbial cells prior to DNA extraction [625, 772] (Figure 17B). At the molecular level, for amplicon sequencing, peptide nucleic acid clamps or mismatched primers targeting specific hypervariable regions can be applied to suppress amplification of plant organelle DNA [773]. For metagenomic studies, emerging approaches such as CRISPR/Cas9‐based depletion and chromatin immunoprecipitation‐based host DNA removal offer promising avenues to overcome current technical limitations [770] (Figure 17B). Collectively, these strategies substantially improve the recovery of microbial signals and provide a solid foundation for downstream multi‐omics analyses aimed at deciphering the functional potential of plant‐associated microbiomes.

Building on optimized sample quality, multidimensional sequencing, and cultivation technologies enable comprehensive exploration of plant microbiomes. High‐throughput culturomics facilitates efficient isolation of individual microbial strains [770, 774], amplicon sequencing captures community diversity [775, 776, 777, 778, 779], and shotgun metagenomics reveals functional potential [778, 779]. Furthermore, integrative multi‐omics approaches, including transcriptomics and metabolomics, provide critical insights into active metabolic processes and plant‐microbe interactions [780] (Figure 17C). Nevertheless, despite these advances, each data acquisition strategy has inherent limitations. For example, while culturomics enables the isolation of easy culturable and fast‐growing microorganisms [781], the majority of microbes in natural environments remain uncultured and still require sequencing‐based approaches for further exploration [782, 783]. Similarly, conventional short‐read sequencing platforms (e.g., Illumina, BGI, GeneMind FASTASeq. 300) can generate large datasets for identifying novel microorganisms and metabolic pathways; however, due to the abundance of repetitive sequences and high strain‐level diversity in plant‐associated ecosystems, genome assemblies are often fragmented and incomplete [784, 785].

To resolve these limitations, emerging sequencing and integrative technologies are reshaping plant microbiome research. Third‐generation long‐read sequencing platforms, such as Oxford Nanopore Technologies (ONT), Qitan Nanopore [786], CycloneSEQ [787], and Pacific Biosciences (PacBio), have emerged as transformative tools in this field [788, 789]. These technologies generate ultra‐long reads capable of spanning complex genomic repeat regions, enabling the direct recovery of full‐length 16S rRNA genes, highly contiguous metagenome‐assembled genomes (MAGs), and even complete circular chromosomes from complex environmental samples [784, 790, 791]. Meanwhile, advanced techniques such as chromosome conformation capture (Hi‐C) metagenomic sequencing, based on chromosome conformation capture, shift the focus from fragmented contigs to real microecological interactions, thereby enhancing genome‐resolved analyses. For example, recent Hi‐C analyses have revealed that phages frequently infect keystone bacteria that are highly connected within co‐occurrence networks, fundamentally impacting soil community interactions [792].

Ultimately, this high‐resolution genomic data not only deepens our understanding of rare taxa within plant microbiomes but also facilitates the precise mining of complete biosynthetic gene clusters (BGCs) with important applications in plant growth promotion and biocontrol [790]. In summary, these technological advances have substantially improved our ability to resolve both the taxonomic composition and genomic potential of plant‐associated microbiomes. The resulting high‐quality, low‐contamination, and multi‐dimensional datasets will provide critical support for subsequent analyses of host microbiota assemblies.

### Data analysis and visualization

After obtaining high‐quality data, the analysis of plant microbiome datasets typically begins with standardized preprocessing workflows, which are essential for ensuring data reliability and comparability. The establishment of analytical pipelines for amplicon and metagenomics microbiome data has significantly improved the reproducibility and lowered the technical barriers associated with plant microbiome bioinformatic analysis.

For amplicon data, integrated workflows such as QIIME 2 [793] or EasyAmplicon [777] provide end‐to‐end solutions covering key steps including sequence quality control, OTU/ASV picking, taxonomic classification, diversity analysis, and biomarker discovery. These approaches enable precise identification of microbial indicators associated with plant health or stress conditions. In contrast, raw metagenomic sequencing data from long‐read platforms require preprocessing steps such as base calling, format conversion, and quality control. For example, raw sequences generated by ONT (*e.g*., Fast5 format) must be converted into FASTQ format using Guppy, followed by adapter trimming and sequence filtering using tools such as Porechop or MinkNOW [785]. For routine quality control, fastp/fastplong is widely used due to its efficiency and comprehensive QC report generation [794] (Figure 17D). Subsequently, tools such as minimap2 [795] and samtools [796] are used to align sequences against plant reference genomes to remove host‐derived sequences, ultimately yielding high‐quality, host‐depleted clean reads for downstream analyses (Figure 17D).

Based on these processed datasets, metagenomic analysis generally follows three major paradigms—read‐based, assembly‐based, and binning (MAG‐oriented)—each addressing different biological questions related to plant‐microbe ecology. Standardized pipelines such as EasyMetagenome [778] further integrate these strategies to enhance analytical consistency and reproducibility. The first approach, read‐based analysis, directly maps sequencing reads to reference databases and rapidly generates taxonomic and functional profiles (Figure 17E). Tools such as Kraken2 [797] and MetaPhlAn4 [798] are commonly used for taxonomic profiling, followed by alpha and beta diversity analyses, while HUMAnN4 [799] is employed for functional profiling. This method is computationally efficient and suitable for large‐scale comparative studies, although it is limited by reference database mainly reported from human study, may lack enough microbe reference genome from plant‐associated microbiome. The second approach, assembly‐based analysis, uses assemblers such as MEGAHIT [800], metaSPAdes [801], or metaMDBG [802] to reconstruct longer contigs from short‐ or long‐read data (Figure 17F). This improves gene prediction accuracy and facilitates the identification of key functional elements such as BGCs, which are central to plant defense and microbial competition in the plant‐associated microbiome. However, assembly quality is often constrained by sequencing depth, community complexity, and strain‐level heterogeneity. The third approach, binning (MAG‐oriented) approach, further organizes contigs clustering into MAGs, which can represent putative draft genomes of microbiome (Figure 17G). Tools such as MetaBAT 2 [803] and MaxBin 2 [804] are widely used for binning, while CheckM2 [805] enables genome quality assessment. This strategy allows the construction of high‐quality genome catalogs and provides a critical bridge from community‐level descriptions to genome‐resolved ecological and functional analyses.

Based on these analytical frameworks, statistical analysis and data visualization play central roles in interpreting plant microbiome datasets. In R/Python ecosystem, packages such as phyloseq [806], microeco [807], amplicon [25], ggplot2 [808], ggtree [809], MicrobiotaProcess [810], patchwork [811], and PyComplexHeatmap [812], enable the generation of high‐quality visualizations, including principal coordinate analysis (PCoA), taxonomic composition, heatmaps, and differential abundance visualizations (Figure 17H). These tools allow clear tracking of microbiome shifts driven by plant genetic traits or environmental perturbations and often incorporate statistical testing within the workflows to ensure robust results. Furthermore, microbial association and network inference methods, including SparCC [813] and statistical and visualization tools ggClusterNet 2 [814], enable the identification of co‐occurrence patterns, modular structures, and keystone taxa. These approaches provide deeper insights into community stability and ecological interaction mechanisms, helping to identify the ‘hub’ species essential for maintaining plant‐associated microbial homeostasis under abiotic and biotic stress.

More importantly, with the simultaneous acquisition of multi‐omics data, such as metagenomics, transcriptomics, and metabolomics, multi‐omics integration analyses are becoming key tools for deciphering the complex correlations among the structure, function and phenotype of plant microbiomes [815]. They provide important support for revealing the key molecules and regulatory pathways that drive microbiome functions and host interactions, such as linking specific root exudate profiles to the recruitment and activation of beneficial rhizobacteria. Meanwhile, the rapid development of user‐friendly platforms and no‐code analytical tools has greatly enhanced the accessibility and standardization of microbiome data analysis. Platforms such as Qiita [816], MGnify [817], and gcMeta [818] provide data deposited, reproducible, and modular workflows (Figure 17H). While cloud‐based tools including MicrobiomeAnalyst [819], Wekemo Bioincloud [815], OmicShare tools [820], Majorbio Cloud [821], and BMKCloud [822] offer integrated solutions for data processing, statistical analysis, and visualization. Additional tools such as ImageGP [823], iNAP [824], iTOL [825], PhyloSuite [826], EVenn [827], and MicrobiomeStatPlots [828] further support specialized visualization and ecological analysis tasks (Figure 17H). Together, these resources provide integrated solutions for multi‐omics data processing and significantly lower the technical barriers in plant microbiome research. Overall, by integrating standardized preprocessing, multi‐level analytical strategies (*e.g*., reads, assembly, and binning), advanced statistical methods, and interactive visualization platforms, current analytical frameworks are driving plant microbiome research toward greater reproducibility, scalability, and deeper mechanistic insight.

### Emerging technologies and future directions

Beyond the aforementioned standardized analytical frameworks and visualization tools, the emergence of various cutting‐edge technologies is driving plant microbiome research from descriptive community profiling and preliminary functional analysis toward deeper mechanistic understanding. To accurately identify metabolically active plant‐associated microbes from vast reservoirs of dormant soil microorganisms, integrated strategies combining DNA stable isotope probing (DNA‐SIP) with targeted sequencing have become a research hotspot [768] (Figure 17I). DNA‐SIP has already been successfully applied to identify soil bacteria capable of metabolizing polycyclic aromatic hydrocarbons such as naphthalene, phenanthrene, anthracene, fluoranthene, and pyrene [829]. Additionally, emerging microbial single‐cell RNA sequencing (scRNA‐seq) technologies enable the resolution of cell type and developmental stage specific responses of both beneficial and pathogenic microbes within plant hosts [830] (Figure 17J). Concurrently, spatial transcriptomics and high‐resolution fluorescence *in situ* hybridization techniques (e.g., Hi‐FISH) have been applied to microscopic plant tissues, allowing precise visualization and localization of microbial colonization around the root cortex or the stomata of leaves [831] (Figure 17K). Despite the multidimensional insights provided by multi‐omics technologies, the isolation and cultivation of the microbial communities remain indispensable for mechanistic research. To bridge this gap, high‐throughput culturomics combined with porous microfluidic platforms and fluorescence‐activated cell sorting (FACS) technology has greatly expanded the scope of cultivable microorganisms [832]. From active screening and single‐cell analysis to spatial localization and pure culture isolation, the integration of these emerging technologies provides systematic tool support for unraveling the complex mechanisms underlying plant‐microbe interactions.

Although sequencing technologies and bioinformatics methods have advanced rapidly, a central challenge remains: how to translate high‐dimensional and complex omics data into interpretable biological models. Looking ahead, to tackle this challenge, future developments are expected to achieve breakthroughs in three aspects (Figure 17L): First, continuous optimization in third‐generation long‐read sequencing technologies, represented by ONT, Qitan, BGI, and PacBio, will enhance the continuity and completeness of MAGs, enabling more accurate reconstruction of microbial genomes in microbiome. Meanwhile, the combination of single‐cell sequencing [833] and spatial transcriptomics [834] will achieve collaborative analysis of the functional heterogeneity and spatial distribution of the plant microbiome. Second, the deeper integration of multi‐omics datasets will promote research to move from component and metabolic descriptions to the inference and dynamic modeling of cross‐interaction networks, and enable the prediction of key plant phenotypes. Third, with the improvement of sequencing resolution, research will further focus on the dynamic changes of non‐coding RNAs regulation [835], epigenetic modifications [836], and mobile genetic elements [837], thereby deepening the understanding of the mechanisms of plant‐microbe interactions and co‐evolution. Based on these advances, future researches will rely more on the collaborative application of SynComs and gene editing technologies to analyze key interaction mechanisms and guide microbial community engineering practices. At the same time, standardized sampling, multi‐omics quality control, and the establishment of open data platforms will provide important support for cross‐study integration and result reproducibility, accelerating their application in sustainable agriculture.

## TRANSLATIONAL APPLICATIONS AND FUTURE PERSPECTIVES OF THE PLANT MICROBIOME

### Translational applications

The agricultural application of microbial preparations dates back to the 1980s, when effective microorganisms (EM) developed in Japan emerged as one of the world's earliest commercial composite microbial products. Consisting of more than 80 mixed‐cultured microbial strains, EM was promoted in over 60 countries for applications such as soil improvement. Owing to the technological level at that time, its mechanism of action was not systematically elucidated, and it suffered from inherent defects including poor stability and high production cost, which led to its gradual withdrawal from the mainstream market in the early 21st century [838]. This early industrial practice provided valuable experience and reference for the subsequent development of the microbial fertilizer industry. In recent years, the advancement of microbiome sequencing technologies has driven plant microbiome research into a period of rapid development, with single‐strain microbial fertilizers gradually reaching commercialization. Since 2010, technological advances represented by synthetic biology have propelled the industry into the era of synthetic microbial communities. Second‐generation multi‐strain compatible microbial agents launched in the United States and Europe have demonstrated over 40% improvement in field stability compared to single‐strain products, driving the market share of microbial fertilizers to approximately 20% in local agricultural production systems.

China's microbial fertilizer industry began in the 1990s and has now formed a complete industrial system. According to statistics from the Supervision, Inspection and Testing Center for Quality of Microbial Fertilizers and Edible Fungi Strains of the Ministry of Agriculture and Rural Affairs of China, as of 2025, there were more than 12,700 validly registered microbial fertilizer products, over 2380 production enterprises, with an annual output value exceeding 41 billion RMB [839]. The products fall into three major categories, with an annual application area exceeding 33 million hectares, enabling a 15%–20% reduction in chemical fertilizer application and a 20%–30% reduction in pesticide application, representing an important component of China's fertilizer industry. However, there remains a significant gap compared to international advanced levels: first, the industrial structure is relatively low, with microbial fertilizers accounting for only 7.2% of China's fertilizer market, far below the international average of approximately 20%; second, product homogeneity is severe, with over 80% of products belonging to first‐generation microbial agents, and the strain compatibility remains insufficient and field performance is unstable, constituting the core bottlenecks, with about 40% of products exhibiting a coefficient of variation in efficacy exceeding 50%; third, innovation capacity is inadequate, with industrial application of cutting‐edge technologies such as synthetic microbial communities and precise regulation lagging behind, and there is a lack of internationally competitive leading enterprises [839].

Currently, basic research on plant microbiomes is in an explosive growth phase, with a large number of research achievements on synthetic microbial communities in different niches, including the rhizosphere, phyllosphere, endosphere, and mycorrhizosphere continuously emerging [34]. However, industrial application still faces both institutional and technical constraints: at the regulatory level, China's current microbial fertilizer registration system is mainly designed for single‐strain or simply compounded products, and registration standards and evaluation systems suitable for synthetic microbiome products have not yet been established, resulting in difficulties for numerous laboratory‐stage research achievements to enter commercial application; at the technical level, the standardization system for the design, production, and application of synthetic microbial communities has not been established, and the problems of unstable product function and uncontrollable quality have not been fundamentally resolved.

Similarly, mycorrhizal fungi, particularly AM fungi, provide substantial benefits to plant growth and health, establishing them as key tools for sustainable agriculture under the climate change and the urgent need to reduce chemical inputs [55, 59, 840]. ECM fungi have long been used in forestry, where inoculating tree seedlings is a well‑established practice that improves reforestation success, especially on degraded or post‑mining sites [55, 841]. They are also used to cultivate high‑value edible mushrooms such as truffles, with *Tuber melanosporum* inoculation representing a multi‑million dollar industry [55, 842]. However, the greatest agricultural potential lies with AM fungi, which colonize the majority of crop plants, including cereals, legumes, and vegetables [55, 84]. AM fungal inoculation enhances crop yield and quality, improves nutrient use efficiency (particularly P and N), and reduces fertilizer requirements [55, 279, 843].

Despite this promise, several major challenges impede the widespread application of AM fungi. The first is large‑scale inoculum production. As obligate biotrophs, AM fungi cannot be propagated on simple media, making conventional industrial fermentation unfeasible [55]. A recent breakthrough has overcome this century‑old bottleneck: based on the discovery that AM fungi are fatty acid auxotrophs dependent on host lipids, researchers developed a host‑free, asymbiotic culture system [844, 845]. For example, supplementation of a defined medium with myristate (C14:0), strigolactone (GR24), and jasmonate (MeJA) enables axenic cultivation of *Rhizophagus clarus*, yielding abundant, infection‑competent spores. This method provides a foundation for the pure, affordable, and scalable production of AM fungal inoculum [320].

A second challenge is the inconsistent field performance of AM inoculants, as their benefits are context‑dependent and can be constrained by high soil fertility (particularly P), intensive tillage, and crop varieties that have been bred for fertilized conditions and are suboptimal AM hosts [55, 279]. Addressing this challenge will require integrated strategies, including the adoption of “mycorrhiza‑friendly” agronomic practices and the breeding of crops with improved symbiotic compatibility [55, 846]. Synthetic Myc factors, such as short‑chain chito‑oligosaccharides, can further prime AM fungal colonization under field conditions [847]. Ultimately, an integrative approach centered on the mycorrhizosphere is critical. Co‑inoculation with complementary hyphosphere bacteria or the use of tripartite plant‐mycorrhizal fungus‐bacterium consortia can yield synergistic effects that surpass those of single inoculants [59, 381]. By integrating these molecular, microbial, and agronomic advances, we can fully realize the potential of mycorrhizal symbiosis to build a more resilient and sustainable agricultural future.

**FIGURE 18:**
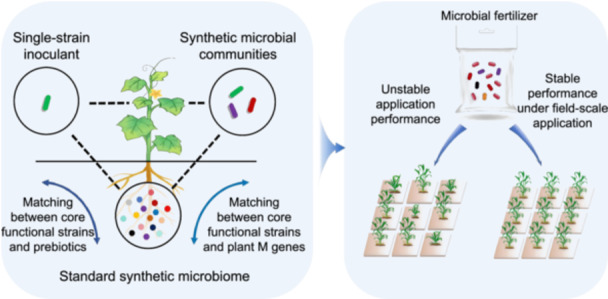
Develop standardized synthetic microbiome products for crops to achieve stable growth promotion, yield increase, and disease resistance at the field scale. The left panel illustrates the developmental trajectory of plant microbiome applications, which has progressed through three distinct phases: initial deployment of single‐strain microbial agents, followed by utilization of simple SynComs, and is poised to ultimately advance toward standardized synthetic microbiome as the next‐generation paradigm. These standardized products are defined by two core technological features: (1) Their formulation consists of rationally designed SynComs paired with tailored prebiotics, where the complex synthetic prebiotics function to stabilize the community structure of SynComs, promote rhizosphere/phyllosphere colonization of the microbial consortium, and enhance expression of their desired biological functions; (2) The core microbial members of the SynComs are genetically matched to the host crop's microbiome regulatory genes (*M* genes), enabling establishment of a bidirectional “crop active recruitment‐functional microbiota targeted colonization” interaction system. The right panel depicts the agricultural outcome following large‐scale application of these standardized products: stable field‐scale performance of microbial inoculants is achieved, whereby a single application of functional microbes to the crop rhizosphere or phyllosphere supports persistent, beneficial interactions with the host plant throughout its entire growth cycle.

Therefore, the development of “standardized synthetic microbiome (SSM)” products with “well‐defined composition, controllable functions, and stable efficacy” represents the core pathway to break through current industrial bottlenecks. The core characteristics of standardized microbiome products should include the following five aspects (Figure 18).

### Streamlined and controllable microbial community composition

The microbial composition of products is limited to 3–10 strains, achieving the functional synergistic effect of “1 + 1 > 2” through rational design based on complementary metabolic pathways and niche coordination among strains. When compatible with mixed fermentation technology for multiple strains, production and application costs are significantly reduced, and the stability of viable cell count reaches the level of single‐strain products.

### Precise host‐microbiome matching

Core effective strains should be screened from the heritable core microbiome of target crops and be highly compatible with crop microbiome regulatory genes (*M* genes; *i.e*., key genes that regulate host recruitment of functional heritable microorganisms). This establishes a two‐way interaction system featuring “active recruitment by crops and targeted colonization by functional bacteria”, which substantially improves strain colonization efficiency in the rhizosphere/phyllosphere, thus breaking through the technical bottleneck of “applied but not colonized” faced by traditional microbial agents [848].

### Clear and defined functional mechanisms

The taxonomic status, functional traits, metabolic characteristics, and interactions of all strains in the product have been systematically characterized, with clear metabolic pathways and regulatory mechanisms underlying their core functions. Dynamic tracking of products in the environment can be achieved using marker genes, ensuring the traceability and biosafety of product application.

### Construction of synbiotic technology system

Drawing on mature experience from gut microbiome research, synbiotic products comprising “synthetic microbial communities + prebiotics” are being developed. By adding prebiotic components such as carbon sources and signal molecules preferred by the microbial community, the structure of indigenous microbiomes is regulated, thereby promoting the colonization and functional expression of exogenous functional microbial communities and further improving the field stability of products [586, 849].

### Alternative pathway of functional metabolites

For products whose mechanisms of action and core functions mediated by specific metabolites, cell factories can be constructed through synthetic biology to enable low‐cost large‐scale production of core metabolites, allowing the development of “microbiome‐derived biostimulant” products. This approach circumvents the problem of microbial community colonization being affected by the environment and achieves stable and controllable functions. Such products can serve as an important supplement to synthetic microbiome products, being suitable for field application scenarios with high environmental heterogeneity. Liu et al. systematically deciphered the “chemical language” between plants and microorganisms, revealing the molecular mechanism by which rice directionally recruits beneficial microorganisms and enhances disease resistance by secreting phenylpropanoid compounds such as ferulic acid. The microbial inducer developed based on this mechanism reduces the incidence of rice blast and sheath blight by more than 40%, and reduces the use of chemical pesticides by 35% [850].

### Future challenges for microbiome applications

The translational application of plant microbiomes is at a critical turning point from quantitative to qualitative change. With the 14th 5‐Year Plan for Bioeconomic Development listing microbiome research as a key direction of cutting‐edge biotechnology, along with continuous breakthroughs in core technologies and gradual improvement of policy frameworks, the plant microbiome industry has entered a golden period of rapid development. Entering the 15th 5‐Year Plan period (2026–2030), the national plan has further designated biomanufacturing as a key future industry and positioned agricultural microorganisms as a new engine for green agricultural transformation, injecting fresh momentum into the industrialization of plant microbiome technologies. In the next 5–10 years, China is expected to establish an internationally competitive industrial highland in this field and lead the direction of global agricultural green transformation.

### Future perspectives: A 5–10 year blueprint

Here, we outline below the key scientific questions, technological bottlenecks, and translational roadmaps that will define the frontier of plant microbiome research in the coming decade.

### Key scientific questions

Several fundamental questions remain unresolved and should be prioritized in the next 5–10 years. (1) How do crops discriminate between beneficial and pathogenic microbes at the molecular level, and what specific receptor‐ligand interactions govern this discrimination [429]? (2) How do plants elicit differential immune responses to beneficial microbes versus pathogens? A deeper understanding of the molecular mechanisms by which beneficial microbes overcome plant immunity, and of how these mechanisms differ from pathogen‐triggered immune responses, could enable better exploitation of their probiotic functions of beneficial microbes. (3) What are the molecular identities and mechanisms of the “gatekeeper” organisms that maintain microbiome homeostasis in the phyllosphere and the aerial root mucilagephere [88, 109]? (4) How do cross‐kingdom signals, including small RNAs [317, 318, 319], effector proteins [268, 269], and volatile organic compounds, coordinate community‐level behaviors within the pathobiome [37]? (5) What is the spatiotemporal architecture of microbiome assembly at single‐cell resolution, and how do individual microbial cells communicate within the complex tissue environments of roots and leaves [324, 831]? (6) Can we establish causal links in the host gene‐host metabolite‐microbiome‐phenotype chain across diverse crop species, thereby moving beyond correlative multi‐omics associations [670, 677]?

### Technological bottlenecks

The translation of microbiome science into practice faces several critical technological hurdles. (1) Host DNA contamination continues to impede microbiome profiling in low‐biomass tissues such as the phyllosphere and endosphere; more efficient depletion methods, including programmable CRISPR‐based systems and improved nucleic acid capture probes, are urgently needed [593, 770]. (2) The vast majority of plant‐associated microorganisms remain uncultured, limiting functional validation; high‐throughput culturomics integrated with microfluidic platforms and FACS must be further developed to close this “cultivation gap” [832, 851]. (3) Stable genetic transformation of AM fungi remains elusive, restricting functional analysis of key symbiosis genes; breakthroughs in delivery systems (e.g., nanoparticle‐mediated or viral vectors) and an understanding of AM fungal DNA replication machinery are prerequisites for tractable genetic manipulation [320, 337]. (4) Integrating high‐dimensional multi‐omics (e.g., metagenomics, metatranscriptomics, metabolomics) and host phenomics data into predictive models of field performance remains a formidable computational challenge that requires standardized data frameworks and community‐wide collaborative platforms [5, 153].

### Translational roadmaps

We envision four synergistic translational pathways that will mature over the next decade. First, the development of “third‐generation” microbiome products, including the SSM products with well‐defined composition, validated mechanisms, and host‐genotype‐matched consortia, will move from proof‐of‐concept to commercial deployment, supported by updated regulatory frameworks that accommodate multi‐strain and genetically engineered inoculants [838, 839]. Second, endo‐microbiome engineering will become a core strategy for sustainable crop improvement. A deep understanding of the functional mechanisms of plant endo‐microbiomes presents a transformative opportunity for sustainable agriculture. The core approach involves screening core functional strains from endo‐microbiomes to construct stable and efficient artificial SynComs [605, 852]. For example, a SynCom derived from the endophytic microbiota of desert plants has been applied to enhance wheat drought tolerance, with *Pseudomonas* identified as the core functional taxon [627]. Harnessing cross‐kingdom (fungal‐bacterial) endophytic SynComs will boost the efficacy of single‐species inoculants, unlocking greater potential for composite endophyte applications [853]. Alternatively, endosymbiotic gene expression can be directly activated in crops, for example, through cyclic nucleotide‐gated channel genes (e.g., *CNGC15*), thereby offering a promising strategy for future self‐sustained crop production [454, 854]. In addition, applying microbial encapsulants (e.g., a polymeric hydrogel consisting of carboxymethyl chitosan, sodium alginate, and calcium chloride, [855]) that preserve the activity of endophytes over time will improve their growth‐promoting efficiency. Crucially, deploying these endo‐microbiome strategies in agricultural contexts requires comprehensive consideration of environmental factors, such as soil nitrogen level and nitrogen form [616, 856, 857], to maximize their functional effects. In the future, integrating multi‐omics technologies, synthetic biology, and microbial coating approaches to precisely manipulate plant and endo‐microbiome interactions will provide important technological pathways for reducing chemical fertilizer use and advancing sustainable agriculture. Third, microbiome‐informed breeding will become an integral component of crop improvement programs. By systematically mapping *M* genes across major crop species and wild relatives, breeders will be able to select for varieties with heritably enhanced capacities to recruit beneficial microbiota [19, 22, 680]. Fourth, precision microbiome management, enabled by AI‐driven predictive models [35, 36], real‐time in‐field microbiome sensors, and site‐specific delivery systems (*e.g*., seed coatings, nanocarriers), will transition microbiome applications from uniform field treatments to customized, environmentally responsive interventions. These four pathways, operating in concert, hold the potential to transform sustainable agriculture by reducing chemical inputs by 30%–50% while maintaining or increasing crop yields under climate stress [39, 850].

In summary, the next decade of plant microbiome research will be defined by the convergence of mechanistic discovery, technological innovation, and translational implementation. This comprehensive review aims to both document and catalyze this trajectory.

## AUTHOR CONTRIBUTIONS

**Mi Wei:** Conceptualization; funding acquisition, writing—original draft, formal analysis. **Xianan Xie:** Conceptualization; funding acquisition; writing—original draft; writing—review and editing; visualization; supervision. **Tengxiang Lian:** Funding acquisition; writing—original draft; visualization. **Liying Chen and Lanxiang Wang:** Funding acquisition; writing—original draft; visualization; writing—review and editing. **Junjie Ye, Xiaofang Yao, and Shilong Duan:** Writing—original draft; writing—review and editing; visualization. **Zhiqiang Pang, Xin Zhou, Zhihui Xu, and Lin Zhang:** Funding acquisition; writing—original draft; visualization. **Mengcen Wang:** Funding acquisition; writing—original draft; writing—review and editing. **Xiaolin Wang and Chang‐Fu Tian:** Funding acquisition; writing—original draft; visualization. **Yong‐Xin Liu:** Funding acquisition; writing—review and editing; writing—original draft. **Kai Sun:** Funding acquisition; writing—original draft; writing—review and editing; visualization. **Ertao Wang:** Funding acquisition; writing—original draft; writing—review and editing; supervision. **Junliang He:** Visualization, software. **Ziyu Lu, Jinji Tu, Hongfu Li, Xin‐Yue Xu, Jie Zhou, and Feiying Zhu:** Formal analysis. **Lam‐Son Phan Tran and Paola Bonfante:** Supervision; writing—review and editing.

## CONFLICT OF INTEREST STATEMENT

The authors declare no conflicts of interest.

## ETHICS STATEMENT

No animals or humans were involved in this study.

## Data Availability

Data sharing is not applicable to this article as no new data were created or analyzed in this review. Supplementary materials (graphical abstract, slides, videos, Chinese translated version, and updated materials) may be found in the online DOI or iMeta Science http://www.imeta.science/.
